# Discovery of new STAT3 inhibitors as anticancer agents using ligand-receptor contact fingerprints and docking-augmented machine learning†

**DOI:** 10.1039/d2ra07007c

**Published:** 2023-02-03

**Authors:** Nour Jamal Jaradat, Walhan Alshaer, Mamon Hatmal, Mutasem Omar Taha

**Affiliations:** a Department of Pharmaceutical Sciences, Faculty of Pharmacy, University of Jordan Amman 11492 Jordan mutasem@ju.edu.jo +962 6 5339649 +962 6 5355000 ext. 23305; b Cell Therapy Center, The University of Jordan Amman 11942 Jordan; c Department of Medical Laboratory Sciences, Faculty of Applied Medical Sciences, The Hashemite University P.O. Box 330127 Zarqa 13133 Jordan

## Abstract

STAT3 belongs to a family of seven vital transcription factors. High levels of STAT3 are detected in several types of cancer. Hence, STAT3 inhibition is considered a promising therapeutic anti-cancer strategy. In this work, we used multiple docked poses of STAT3 inhibitors to augment training data for machine learning QSAR modeling. Ligand–Receptor Contact Fingerprints and scoring values were implemented as descriptor variables. Escalating docking-scoring consensus levels were scanned against orthogonal machine learners, and the best learners (Random Forests and XGBoost) were coupled with genetic algorithm and Shapley additive explanations (SHAP) to identify critical descriptors that determine anti-STAT3 bioactivity to be translated into pharmacophore model(s). Two successful pharmacophores were deduced and subsequently used for *in silico* screening against the National Cancer Institute (NCI) database. A total of 26 hits were evaluated *in vitro* for their anti-STAT3 bioactivities. Out of which, three hits of novel chemotypes, showed cytotoxic IC_50_ values in the nanomolar range (35 nM to 6.7 μM). However, two are potent dihydrofolate reductase (DHFR) inhibitors and therefore should have significant indirect STAT3 inhibitory effects. The third hit (cytotoxic IC_50_ = 0.44 μM) is purely direct STAT3 inhibitor (devoid of DHFR activity) and caused, at its cytotoxic IC_50_, more than two-fold reduction in the expression of STAT3 downstream genes (c-Myc and Bcl-xL). The presented work indicates that the concept of data augmentation using multiple docked poses is a promising strategy for generating valid machine learning models capable of discriminating active from inactive compounds.

## Abstract 2

Novel STAT3 inhibitory lead of potent anti-STAT3 IC50 and novel chemotype was discovered using a data augmentation algorithm based on a computational sequence of docking, scoring, ligand-receptor contact fingerprints.

## Graphical abstract

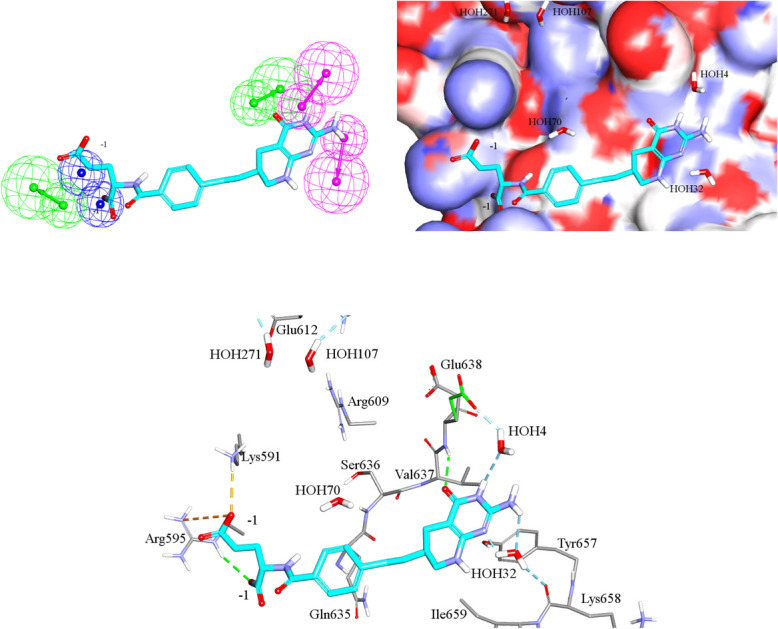

## 1. Introduction

**Fig. 1:**
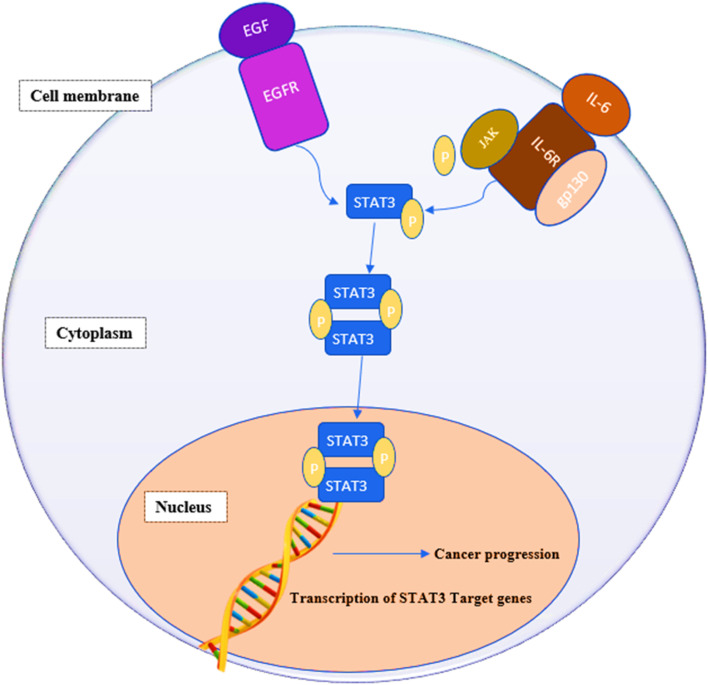
IL-6/STAT3 signaling pathway in cancer cells. IL-6 binds to membrane-bound IL-6 receptors α (IL-6R) and β (also known as gp130). The IL-6/IL-6R/gp130 complex activates phosphorylation of JAKs, followed by STAT3 phosphorylation and activation. Growth factors, such as FGF, IGF and EGF, can also phosphorylate STAT3 by binding to their membrane receptors. Phosphorylated STAT3 dimerizes and translocates into the nucleus where it binds to the promotor region of target genes and activates their transcription.

STAT3 is a member of the “signal transducers and activators of transcription STATs” family of oncogenic transcription factors. This family also includes STAT 1, 2, 3, 4, 5a, 5b and 6. These transcription factors remain latent in the cytoplasm until being activated by cytokines, *e.g.*, interleukin-6 (IL-6) and growth factors (FGF, IGF and EGF) at which point they get phosphorylated, dimerize and move to the nucleus, where they begin activating the transcription of various genes involved in a variety of cellular processes.^1,2^ STAT3 affects genes associated with proliferation (*e.g.*, Bcl-2, Bcl-xL, survivin, cyclin D1, c-Myc and Mcl-1), angiogenesis (*e.g.*, Hif1 and VEGF) and epithelial–mesenchymal transition (*e.g.*, vimentin, TWIST, MMP-9 and MMP-7).^3,4^Fig. 1 summarizes the signaling pathway of STAT3.^5^

STAT3 is extensively expressed in a variety of cancers such as human solid tumors.^5,6^ Blocking constitutively active STAT3 signaling causes tumor cells to die but has little effect on healthy cells. Additionally, STAT3 inhibition attenuates resistance to anticancer chemo- and radiotherapy.^7^ Furthermore, STAT3 inhibition prevents the transition of normal cells into tumor cells making this oncogenic protein an attractive target for cancer drug discovery.^8–10^

**Fig. 2:**
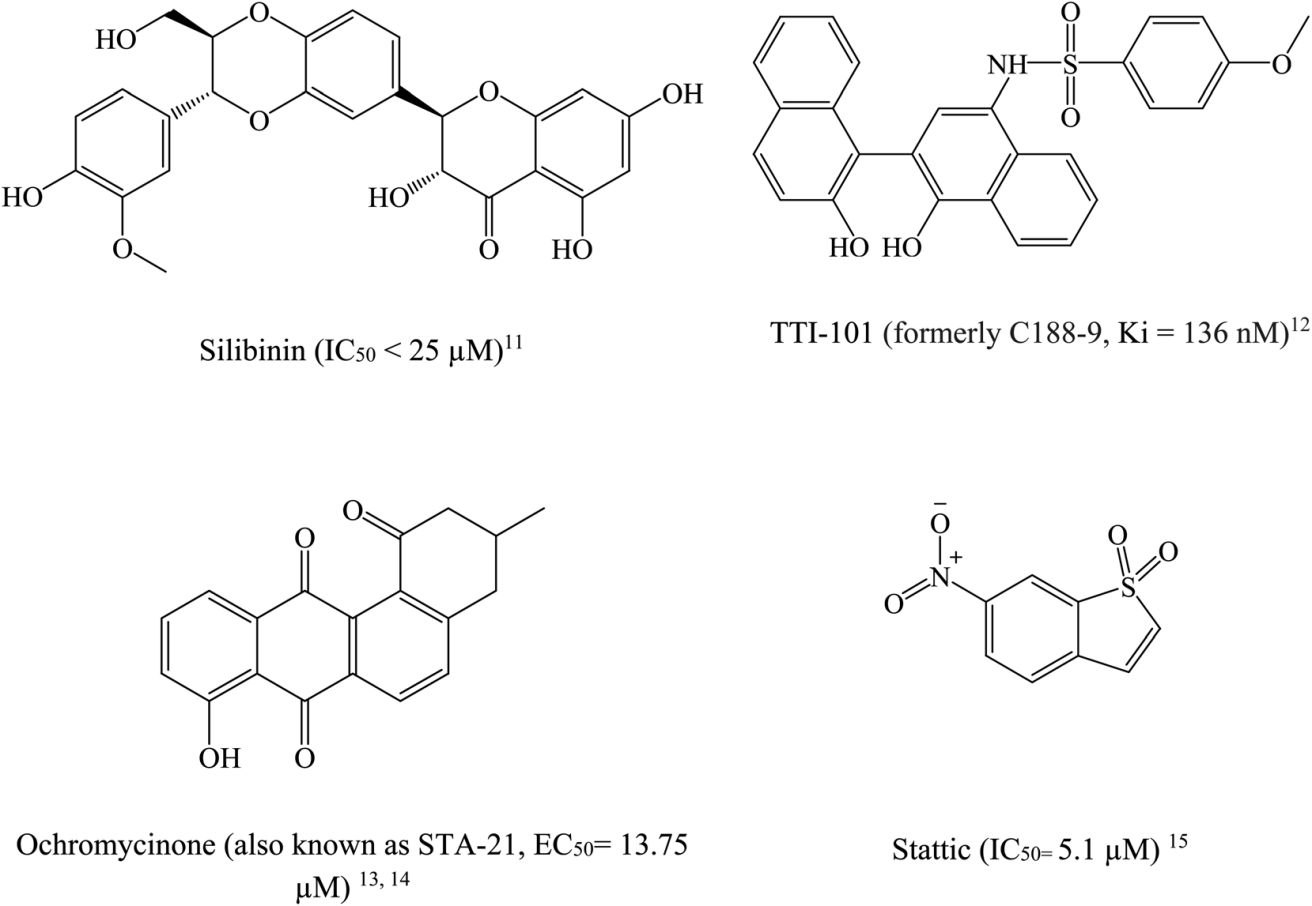
Chemical structures of some reported direct STAT3 inhibitors.

STAT3 inhibitors can be classified based on their mode of action into direct or indirect blockers. Direct inhibitors bind STAT3 domains, while indirect inhibitors affect STAT3 through cellular networks.^9^Fig. 2 shows examples on potent STAT3 direct inhibitors.^11–15^

Ligand–Receptor Contacts Fingerprint (LRCF) is a binary vector made up of bins filled with “ones” or “zeroes” corresponding to binding site atoms in the target protein that either engage or avoid a docked ligand pose.^16–19^

Machine learning (ML) in molecular modelling is the application of statistical approaches to learn and predict molecular properties.^20,21^ Some of the most often applied machine learning algorithms in drug design and discovery applications include: eXtreme Gradient Boosting (XGBoost);^22^ Random Forest (RF);^21^ Naive Bayesian (NB);^23,24^*k*-nearest neighbors (*k*NN);^17^ Probabilistic Neural Networks (PNN)^25,26^ and multilayer perceptron MLP^27^(see ESI section SM2†).

The term data augmentation refers to methods to create additional training samples that will ultimately enhance machine learning model performance and reduce overfitting.^28^

In the current project we used numerous docked poses, generated by multiple docking engines and scoring functions for a list of active and inactive STAT3 ligands, to augment bioactivity ML classifiers. LRCFs and scoring function values were implemented as descriptors in ML models to classify STAT3 ligands into “active” or “inactive” categories. Since docking algorithms are usually successful in achieving enthalpically reasonable docked poses, especially for potent ligands, it can be reasonably assumed that ML-based agreement on a specific set of contact atoms inside the binding site (*i.e.*, LRCFs) underlines their ability (*i.e.*, the particular set of contact atoms) to classify docked virtual hits as being active or inactive.^19^

**Fig. 3:**
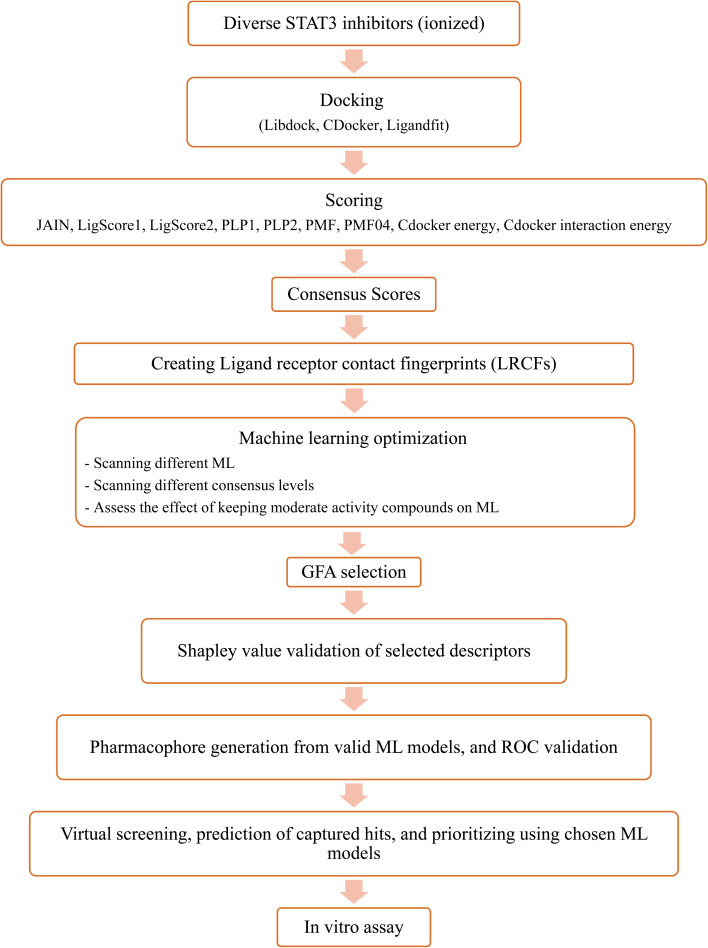
Summary of the workflow implemented in the current project.

Upon testing many orthogonal MLs, the best performing MLs were paired with genetic function algorithm (GFA) to pinpoint particular descriptors (ligand–receptor contacts and/or scoring functions) that best explain bioactivity variation among training and testing compounds. The relative contribution of each descriptor in bioactivity class predictions was explained using Shapley values (SHAP).^29,30^ Subsequently, pharmacophore models were built based on GA-selected descriptors of consistent SHAP probabilities. Valid models were utilized as 3D search queries to look for novel STAT3 inhibitors from the NCI's database. Fig. 3 summarizes the workflow implemented in this study. High ranking hits were tested *in vitro*.

## 2. Materials and methods

The following software packages were used in this project:

• BIOVIA DiscoveryStudio (Version 4.5), Biovia Inc. (https://www.3dsbiovia.com/), USA.

• *In house* built package to generate ligand–receptor contacts fingerprints written in Fortran.

• KNIME Analytics Platform (Version 4.3.3), https://www.knime.com/.

• CS ChemDraw Ultra (Version 7.0.1) Cambridge Soft Corp. (http://www.cambridgesoft.com), USA.

• Marvin View (ChemAxon Ltd., USA).

### 2.1 Data collection

STAT3 inhibitors were mined from the European Bioinformatics Institute database (ChEMBL) (https://www.ebi.ac.uk/chembl/).^59–61^ The collected compounds (935 antagonists) were carefully checked for errors, duplicate structures, and chirality. Erroneous structures, duplicates and racemic compounds were excluded, and only ligands that bind specifically to the SH2 domain were kept leaving 314 remaining inhibitors. These were divided into 116 actives (IC_50_ ≤ 5000 nM), 92 moderates (5000 nM < IC_50_ < 20 000 nM), and 106 inactives (IC_50_ ≥ 20 000 nM). Ligands were ionized as guided by Marvin View (ChemAxon Ltd., USA) at pH of 7.4. Accordingly, amine groups were protonated and assigned formal positive charge, while carboxylic acids, phosphoric acids, and sulfonamides were deprotonated and assigned formal negative charges. ESI Table S1† lists the chemical structures of modeled compounds in SMILE formats together with their reported bioactivities.

### 2.2 Molecular modeling

#### 2.2.1 Docking

The Protein Databank (PDB) was mined for STAT3 crystal structures. The search identified 32 protein structures^31–38^ that were downloaded and visualized in Discovery Studio 4.5. Nine are STAT3 protein structures (listed in ESI Table S2†), the others are STAT3-related cellular signaling cascade proteins and were therefore neglected. Two of the nine STAT3 structures are short fragments and unsuitable for docking purposes (namely, 4ZIA and 5U5S, respectively). However, only two of the remaining seven structures included co-crystalized ligands, namely, 6NJS and 6NUQ, and were therefore amenable for docking. We opted to select 6NJS over 6NUQ based on its superior resolution (2.70 *vs.* 3.15 Å). Moreover, 6NJS is free from mutations in the SH2 domain and has the fewest gaps (discontinuities) in its sequence. Hydrogen atoms were introduced to the protein using Discovery Studio 4.5 templates for protein residues, hydration water molecules were kept, and the protein structure was used in docking experiments without energy minimization. The binding pocket was defined as the cavity volume occupied by the co-crystallized ligand (PDB code KQV). Three docking engines were employed, namely, LibDock,^39,40^ LigandFit,^41^ and CDOCKER^42^ to dock the collected compounds (314 compounds, ESI Table S1†) into the binding pocket of STAT3. Details about the docking experiments and related parameters are provided in ESI Section SM1.†

#### 2.2.2 Scoring of docked poses

The docked poses were scored using 9 generally orthogonal scoring functions (see ESI Table S3† for cross-correlation matrix), namely, LigScore1, LigScore2,^43^ Jain,^44^ PLP1, PLP2,^45^ PMF, PMF04,^46^ CDOCKER energy and CDOCKER interaction energy.^47^ Each docked pose was further scored by consensus among the same 9 scoring functions. The implemented consensus function assigns a value 1 for any molecular pose ranked within the highest 20% of certain scoring function; otherwise, it assigns the docked pose a zero value (*i.e.*, ranked within the lowest 80%). Subsequently, the consensus function sums up the scores for the particular molecular pose/conformer for ranking.^48^

#### 2.2.3 RMSD filtering

The RMSD filter of Discovery Studio 4.5 was employed. It calculates the Root Mean Square Deviation (RMSD) of docked poses (in Å). Heavy atoms were included for RMSD calculation (*i.e.*, without hydrogen atoms). Docked poses of a particular compound of RMSD <2.0 Å were considered duplicates and the one having highest consensus score was kept for subsequent processing while others were discarded.

#### 2.2.4 Generation of ligand-receptor contacts fingerprints (LRCFs)

Contact atoms in the binding site were determined by evaluating poses/conformers of docked compounds: a binding site atom that is within 2.5 Å of any atom in the docked ligand pose/conformer is given an intermolecular contact value of “one” otherwise it is given a contact value of “zero”. Automatic distance computations were performed utilizing an *in house* designed FORTRAN software.^19^ Eventually, a 2D binary matrix of zeros and ones is created, with each row representing specific docked ligand pose and each column representing a distinct binding site atom. For each docked pose, rows are referred to as LRCFs, so each docked pose has its own LRCF.

### 2.3 Machine learning

All elements of machine learning (ML), such as scanning different learners, selecting descriptors using a genetic algorithm (GA), and evaluating models using accuracy, Cohen's kappa values, and Shapley values (SHAP), were done using graphical programming within the KNIME analytics platform (Version 4.3.3).

#### 2.3.1 Scanning for optimal machine learner and docking-scoring consensus levels

The collected compounds (Table S1, under ESI†) were split into training and testing sets. Splitting was performed randomly by ranking the docked poses according to their bioactivity classes (actives, intermediates and inactives), then selecting every fifth compound, including all corresponding docked conformers/poses, for the testing sets. The remaining compounds (including all their docked poses) were used as training sets. However, the count of training and testing compounds gradually decreased in response to escalating docking-scoring consensus levels (see Section 3.1) because the docked poses of some ligands failed to achieve higher scoring consensus levels (*i.e.*, they failed to collect enough scoring votes to achieve the required consensus) and thus were excluded from the lists (*i.e.*, training and/or testing). Still, a training-to-testing ratio of *ca.* 80-to-20% is generally maintained. Table 2 shows the effect of consensus docking score levels on docked poses counts.

To evaluate different MLs, LRCFs and scoring functions values (LigScore1, LigScore2, PLP1, PLP2, PMF, PMF04, JAIN, CDocker-energy, and CDocker-interaction energy) were considered as the independent variables (descriptors), while the corresponding activity classes (active, inactive and intermediate) were considered as the response. Six orthogonal MLs were scanned, namely, Random Forests (RF),^21^ eXtreme Gradient Boosting (XGBoost),^22^ Naive Bayes (NB),^49^ Probabilistic Neural Network (PNN),^25,26^*k*-Nearest Neighbors (*k*NN),^17^ and Multilayer Perceptron (MLP).^27^

The classification power of each ML was judged based on its ability to correctly classify the docked poses of training and testing ligands into actives, inactives and intermediates. Two ML success criteria were considered, namely, accuracy (eqn (1))^50^ and Cohen's Kappa values (eqn (2)).^51^1
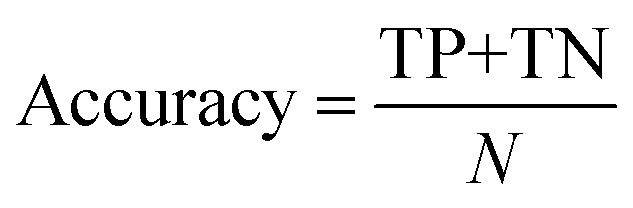
where, TP is the true positive (correctly classified actives), TN true negatives (truly classified inactives), and *N* is the total number of evaluated compounds.2
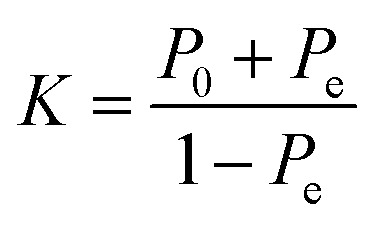
where *P*_0_ is the relative observed agreement among raters (*i.e.*, accuracy), and *P*_e_ is the hypothetical probability of chance agreement. This is done by using the observed data to calculate the probabilities of each observer randomly seeing each category. If the raters are in complete agreement, then kappa = 1. If there is no agreement among the raters other than what would be expected by chance (as given by *P*_e_), kappa = 0. Negative Cohen's Kappa value implies the agreement is worse than random.^51^

Evaluation against the training set involve removing 20% (*i.e.*, leave-20%-out or 5-fold cross-validation) of the data points (*i.e.*, docked poses), then building the particular ML-QSAR model from the remaining 80% data. The model is then used for classifying the removed 20% compounds. The process is repeated until all training data points are removed from the training list and predicted at least once. Accuracy and Cohen's Kappa values were calculated based on comparing classification results with actual bioactivity classes. Evaluation against the testing set involved calculating the accuracy or Cohen's Kappa values of the particular ML-QSAR model by comparing its predicted classification results with the actual bioactivity classes of the external testing set.^52,53^ Details about MLs are provided in ESI Section SM2.†

#### 2.3.2 Genetic function algorithm-based ML-QSAR modelling

Genetic function algorithm (GFA) was coupled to best-performing MLs (either RF or XGBoost) to search for the best possible combination of descriptors (LRCFs and docking scoring values) capable of explaining bioactivity classes of training and testing docked poses/conformers. Only docked conformers/poses of active and inactive ligands in ESI Table S1† (intermediates were excluded) of at least a consensus score level of 1 were included (represents the best performing docking/scoring consensus level, see Section 3.3). GFA operates through a cycle of four stages:^54^ (i) encoding mechanism: a gene-based encoding system is implemented herein, whereby the presence or absence of a certain descriptor(s) in a suggested model is encoded by chromosome format. That is, each potential ML model is represented as vector (chromosome) composed of string of bins (genes), whereby each bin (gene) represents a particular independent variable (descriptor), such that if a particular bin is filled with “0” then the corresponding descriptor is absent from the corresponding model under evaluation, while if the bin is filled with “1” then the corresponding descriptor is present in the model. (ii) Definition of a fitness function: each chromosome is associated with a fitness value that reflects how good it is compared to other solutions. Cohen's Kappa (eqn (2))^51^ was used in the current project as fitness function. (iii) Creating a population of chromosomes. (iv) Genetic manipulation of chromosomes through mating and mutation to yield new generations of chromosomes.^54^

Two subsequent GA-phases were implemented in the current project: An initial preliminary simplistic phase was performed with population size and genetic iterations of 50 and 100, respectively, to narrow down the number of descriptors from 471 to 50. A subsequent more thorough GFA phase was performed on this list of descriptors with population size and genetic iterations of 500 and 5000, respectively, to refine the descriptors into a range of 10 to 20 variables.

#### 2.3.3 Assessment of descriptor contributions in GA-ML models using shapley values

Shapley additive explanation (SHAP) value of a particular feature for certain observation, *e.g.*, docked conformer/pose, indicates the degree this feature has contributed to the deviation from base-line prediction (the mean prediction over the full sampling data) for that particular observation.^29,55^ This technique evaluates the effect of any particular feature in an ML model by removing the influence of that feature from the corresponding model and building coalition from the remaining features. SHAP then evaluates the deviation in prediction probability associated with removed feature. Feature exclusion proceeds by summarizing the validation set using *k*-means to create feature sampling table to be used when creating coalitions. The number of k-means was set to 100.

### 2.4 Pharmacophore generation from docked poses

The Receptor–Ligand Pharmacophore Generation Protocol of Discovery Studio 4.5 was used to extract a maximum of 10 pharmacophore models from a docked pose of 115 (most potent within the testing list, IC_50_ = 136 nM, ESI Table S1†) selected because it has the highest probability contributions towards “Active” label by GFA-selected descriptors as determined by SHAP analysis (within the respective optimal ML model, see Section 3.4). This protocol (*i.e.*, Receptor–Ligand Pharmacophore Generation Protocol) selects certain subsets from ligand–receptor binding interactions and translates them into pharmacophore models. The following settings were implemented in this protocol: binding site hydration water molecules were kept, range of allowed number of features = 4 to 6. The generated pharmacophores were ranked according to rules-based selectivity scoring function.^56^ Maximum charge–charge interaction distance = 8.0 Å (if the distance between a charged feature in the ligand and its nearest protein counterpart is longer than this value the electrostatic features will be removed). Maximum hydrogen bond distance = 4.0 Å (if the distance between two hydrogen-bonded heavy atoms is larger than this value then no hydrogen bonding feature will be added). Maximum hydrophobic distance = 5.5 (this is the maximum distance in Å between the center of a hydrophobic feature in a ligand and the nearest hydrophobic residue to permit adding hydrophobic feature). Maximum exclusion volume distance = 4.0 Å (this setting generates pharmacophore models without exclusion volumes). Minimum interfeature distance = 1.0 Å (this is the minimum distance between features in Å). The generated pharmacophores were validated using receiver operating characteristic (ROC)^50,57,58^ against a list of active (116) and inactive (106) STAT3 inhibitors extracted from ChEMBL^59–61^ with a maximum of 100 conformers per ligand.

### 2.5 *In silico* screening for new STAT3 inhibitors and bioactivity prediction using ML models

Optimal pharmacophore models were employed as 3D search queries to screen the national cancer institute (NCI) list of compounds. Screening was performed employing the ‘‘Search 3D Database’’ protocol implemented within Discovery Studio (Version 4.5). Top 100 hits of highest fit values against each pharmacophore were docked into STAT3 protein (PDB code: 6njs) using docking-scoring settings that were used with the corresponding models (mentioned in section 2.2.1 to 2.2.2). Subsequently, the docked poses were filtered according to docking scoring consensus level (*i.e.*, ≥1) and RMSD filter (2.0 Å) (see Section 2.2.3). Corresponding LRCFs and scoring values were substituted in the respective ML models (RF or XGBoost) to predict the activity label of each docked pose/conformer. This resulted in a situation where each screened compound yielded a set of poses that are assigned either “active” or “inactive” labels. Therefore, a threshold was defined to consider certain hit molecule as being promising or not. It was decided to define such a threshold based on predicted active/inactive poses ratios within the corresponding testing set. The least active-to-inactive ratio of unequivocally documented active inhibitor was used as threshold for prioritizing hits.^19^Table 6 shows the percentages of active poses of testing compounds as predicted by the top two ML (*i.e.*, XGboost and RF). Hits predicted to exceed the proposed active/inactive ratio threshold were requested from the NCI. However, only subset of the requested compounds were readily available from the NCI.

### 2.6 Bioassay of NCI hits

Acquired hits from the NCI were bioassayed by two methods: (i) MTT cytotoxicity test^62^ against a panel of cell lines, and (ii) polymerase chain reaction (PCR) to detect the expression of STAT3-downstream genes: c-Myc, and Bcl-xl (see ESI Section SM3†).

#### 2.6.1 Cell viability assay using MTT bioassay

A panel of 10 cell lines (Fibroblasts, HEK293, 3T3, PANC1, DU145, U87, MDA-MB-231, A549, doxorubicin resistant and sensitive MCF7) was screened against the selective STAT3 inhibitor pyrimethamine, at 10 μM, to identify cells that rely on STAT3 expression for their livelihood and proliferation.^63–65^ The cytotoxicity of pyrimethamine against the selected cell lines was assessed using MTT procedure (see ESI Section SM3† for details). HEK-239, MCF-7, U87, MDA-MB-231 and Fibroblasts were found to be good indicators of STAT3 significance/redundancy (see results Section 3.7, Table 7). Thereafter, the acquired hits were evaluated, at 10 μM, against HEK-239, MCF-7, U87, MDA-MB-231 and Fibroblasts, to identify inhibitors exhibiting similar cytotoxic patterns to pyrimethamine. Potent hits of percent inhibition against HEK293 cells ≳ 50% were further evaluated at 12 escalating concentrations (0, 0.006, 0.012, 0.023, 0.047, 0.095, 0.190, 0.375, 0.750, 1.500, 15.000, and 30.000 μM) to construct their corresponding dose/viability curves and to determine their IC_50_ values. Stattic and pyrimethamine were used as standard positive controls with IC_50_ values 1.57 μM and 5.12 μM, respectively. IC_50_ values were calculated using nonlinear regression of the log(concentration) *vs.* viability percentage values using GraphPad Prism 7.0.

#### 2.6.2 Quantitative polymerase chain reaction (qPCR)

qPCR was performed to determine the expression of c-Myc and Bcl-xl genes (both are downstream of STAT3, ESI Table S5†) at mRNA level. The housekeeping genes 18srRNA and actin-β were used as reference to normalize the expression levels of the measured genes. Each sample was examined in triplicates, and the mean PCR cycle number (*C*_t_) value was calculated. Expression data were analyzed according to ΔΔ*C*_t_ method^66^ using CFX Maestro™ Software – Bio-Rad. A change is considered significant at *α* = 0.05 (see ESI Section SM3† for detailed qPCR protocol).

## 3. Results and discussion

Mining ChEMBL database for STAT3 inhibitors identified 930 inhibitors. Following data curation (deleting duplicates and indirect inhibitors) furnished 314 direct STAT3 inhibitors, out of which 116 ligands had IC_50_ values ≤ 5000 nM were labeled as “active”, 92 compounds of IC_50_ values ranging from 5000 to 20 000 nM were labeled as “intermediate”, and 106 ligands of IC_50_ values ≥ 20 000 nM were allocated “inactive” labels.

### 3.1 Scanning different docking-scoring consensus levels and machine learners

The collected compounds were docked into the binding pocket of STAT3 (PDB code: 6njs) using three docking engines (LibDock, LigandFit and CDocker). Docked poses were pooled and scored by 9 docking scoring functions (generally orthogonal, see ESI Table S3† for cross correlation matrix). Consensus scoring was performed in such a way that if a docked pose scored within the top 20% of a particular scoring function it receives the vote of this scoring function. Summing up votes of different scoring functions yields consensus scoring of the particular docked pose.^19^ However, we opted to remove closely similar docked poses/conformers to avoid noise leading to machine learning over-fitting errors.^67^ Accordingly, any cluster of docked poses within RMSD ≤ 2.0 Å was represented by a single pose (of highest consensus score) in subsequent steps. Table 1 details the counts of docked poses before and after RMSD filtrations for the ionized docked ligands.

**Table 1:** Counts of docked poses before and after RMSD-based filtrations

	Count of poses			
	Actives	Intermediate	Inactive	Total
Docked poses before RMSD filtering	18 143	14 381	16 022	48 546
Docked poses after RMSD filtering	13 408	10 993	10 794	35 195

Clearly from Table 1, although the RMSD filtering reduced the number of docked poses, still, the remaining poses are significant augmentation of the collected modelled compounds (314 collected ligands were augmented to 35 195 docked poses).

We propose that the convergence of high-quality docked poses (*i.e.*, reasonable docking scoring consensus) of active ligands on specific, distinct binding site contacts, while the same contacts are avoided by docked poses of inactive or intermediately-active ligands, underlines the significance of these contact points as discriminators of bioactivity classes. However, it is necessary to identify the best possible docked poses that (i) augment training and testing data and (ii) define significant discriminatory binding site contacts. Moreover, it is necessary to identify optimal machine learner(s) for this purpose.

**Fig. 4:**
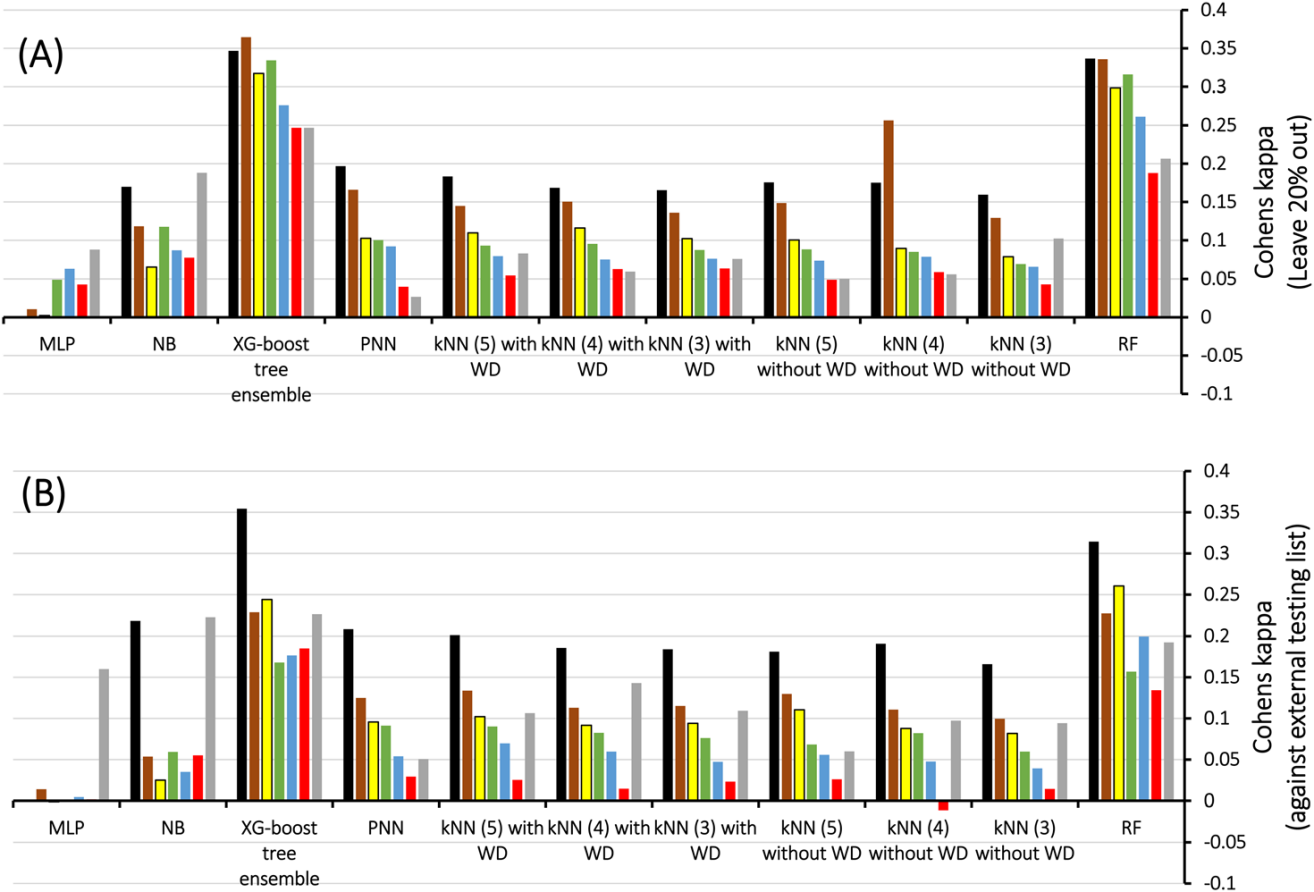
Scanning Cohen's Kappa values against different MLs using LRCFs and scoring functions generated for docked poses of: (A) Training compounds, (B) Testing compounds, WD encode for *k*NN weighted distances, numbers in brackets correspond to count of nearest neighbors. Consensus scoring levels are color coded as follows: 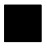 at least 1, 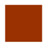 at least 2, 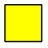 at least 3, 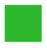 at least 4, 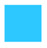 at least 5, 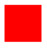 at least 6 and 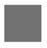 at least 7 consensus level.

Therefore, we scanned the effects of escalating docking-scoring consensus levels and orthogonal machine learners on the classification capacities of corresponding ML models as reflected by their Cohen's Kappa values (Fig. 4).^51^ However, it must be mentioned that escalating the level docking-scoring consensus reduces the counts of docked training and testing compounds and corresponding counts of docked poses, which might undermine our intended data augmentation leverage *via* multiple docked poses.

Fig. 4 shows Cohen's Kappa values for several machine learners (RF, *k*NN XGBoost, PNN, NB, and MLP) at different docking-scoring consensus levels for training and testing sets. Clearly, RF and XGBoost were the best performing machine learners. Additionally, the results of the training and testing data highlight a consensus level of “at least one” (≥1) docking-scoring function to achieve best results among training and testing data. Interestingly, the corresponding counts of docked poses (Table 2) suggest significant data augmentation at this scoring consensus level (*i.e.*, ≥1). That is, 249 training compounds were augmented to 16 317 docked poses (Table 2), while 61 testing compounds were augmented to 4017 docked poses.

**Table 2:** Summarizes the effects of docking-score consensus levels on docked poses counts. Obviously, consensus levels of at least 8 and 9 diminished (rather than augmented) the count of docked poses below the original count of training and testing compounds (*i.e.*, 314 ligands). Therefore, we decided to exclude these two levels of consensus scoring from subsequent machine learning studies

Count of docked poses (corresponding count of compounds in brackets)								
Level of consensus score	Training				Testing			
	Actives	Inter-mediate	Inactives	Total	Actives	Inter-mediate	Inactives	Total
≥1	7637(91)	4303(73)	4377(85)	16 317(249)	1792(22)	1134 (18)	1091(21)	4017(61)
≥2	5854(88)	2967(72)	3243(81)	12 064 (241)	1347(21)	742(18)	720(21)	2809(60)
≥3	4453(88)	2335(72)	2240(76)	9028(236)	1065(21)	563 (17)	740 (20)	2368 (58)
≥4	3815(88)	1896(70)	1970(75)	7681(233)	918(21)	485(18)	472(19)	1875(58)
≥5	3114(88)	1460(71)	1364(68)	5938(227)	740(21)	336(17)	418(18)	1494(56)
≥6	2236(87)	910(64)	782(56)	3928(207)	519(21)	195(16)	243(14)	957(51)
≥7	831(86)	313(49)	284(46)	1428(181)	206(22)	78(13)	57(11)	341(46)
≥8	59(37)	32(10)	25(9)	116(56)	15 (9)	7(3)	3(2)	25 (14)
≥9	12(12)	4(3)	3(3)	19(18)	3(2)	1(1)	1(1)	5 (4)

Table 3 shows the influence of incorporating/deleting intermediately-active compounds on resulting ML models. Unsurprisingly, deleting this class enhanced the corresponding ML models. This is not unexpected since moderate compounds create noise in ML models and hence should be hard to classify. Accordingly, it was decided to exclude moderate compounds from subsequent ML modeling.

**Table 3:** Influence of incorporating intermediate-activity ligands on the success criteria of the resulting ML models

Learner	Intermediate activity class	Accuracy		Cohen's Kappa	
		L20% out^a^	Testing^b^	L20% out^a^	Testing^b^
XGboost	With	0.60	0.60	0.35	0.35
	Without	0.77	0.76	0.49	0.46
RF	With	0.60	0.58	0.34	0.31
	Without	0.77	0.76	0.47	0.45

a L20% out: leave 20% out cross-validation for accuracy and Cohen's Kappa.

b Testing: accuracy and Cohen's Kappa determined against the testing set (marked with a in Table S1).

### 3.2 Veracity of the selected docking scoring settings

**Fig. 5:**
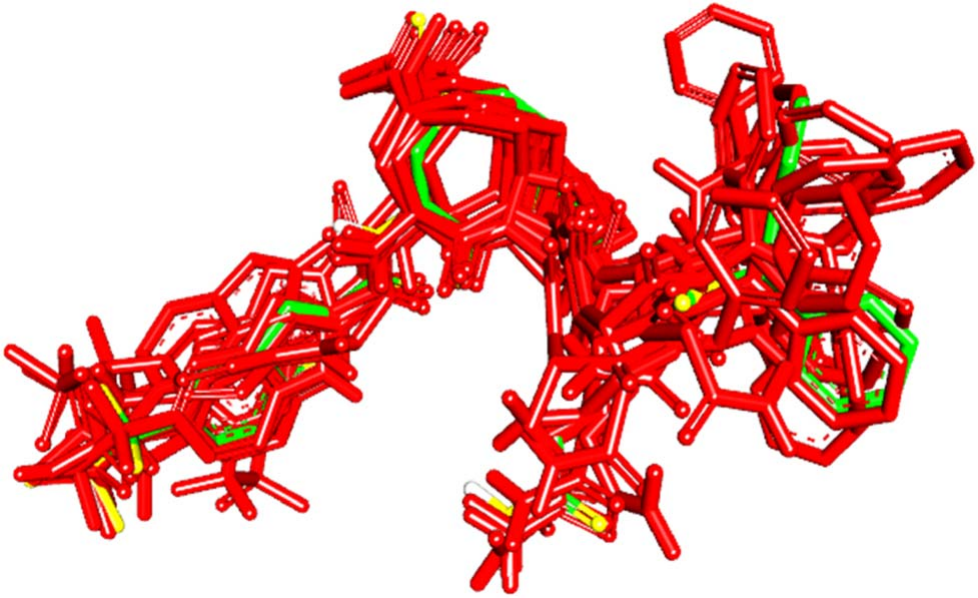
High-ranking docked poses (red) compared to crystallographic bound pose (green) of KQV (PDB code: 6NJS).

To evaluate the veracity of the docking-scoring consensus level ≥1, we compared the docked poses of a co-crystallized ligand (PDB code: KQV), at scoring consensus ≥1, with crystallographic pose of the same ligand. Interestingly, out of 48 docked poses of scoring consensus ≥1, 5 were of RMSD ≤ 2.00 Å from the experimental bound pose, while 10 were of RMSD ≤ 2.50 Å. Fig. 5 shows the best docked poses compared to the crystallographic bound pose of KQV. These results highlight the ability of docking-scoring consensus level ≥ 1 to reproduce the crystallographic pose among its solutions.^19,68–70^

### 3.3 Building genetic algorithm–machine learning models

In order to identify important descriptors that control bioactivity category within training and testing docked poses (at docking-scoring consensus ≥1), we felt it was necessary to couple optimal MLs with genetic function algorithm (GFA). GFA-ML modeling commenced by pooling the values of nine docking-scoring functions (see experimental part) with 471 ligand/binding site contact points as descriptors. The bioactivity class (*i.e.*, active or inactive) was enlisted as dependent response variable. Descriptors were allowed to compete within the context of GFA tournaments using Cohen's Kappa of the resulting models as GFA fitness criteria.^54^ The GFA-ML models were validated by external testing as well as internal leave-20%-out cross validation. Table 4 shows the selected descriptors and statistical results of the two top classifiers.

**Table 4:** Accuracy and Cohen's Kappa values for ML models developed using different ML learners combined with LRCFs and scoring function values as descriptors

Learner	Features selector^a^	Descriptors^b^	Accuracy		Cohen's Kappa	
			L20% out^c^	Testing^d^	L20% out^c^	Testing^d^
Xgboost	GFA	Ligscore2, PLP2, PMF, PMF04, Cdocker energy, GLU 594 HG2, SER 613 HN, TRP 623 CZ2, VAL 637 HA, GLN 643 HG1, GLY 656C, TYR 657 HE1, LYS 658 HG1, MET 660 HB1, MET 660 HE2, MET 660 O, PRO 669 CB, HOH 32 OH2, HOH 60H2, HOH 107 OH2	0.744	0.734	0.414	0.404
	GFA + SHAP	PLP2, PMF, Cdocker energy, SER 613 HN	0.714	0.719	0.348	0.368
RF	GFA	PMF04, Cdocker energy, Cdocker interaction energy, SER 613 HG, GLN 633 HE22, PRO 639 CD, TYR 640 HE1, TYR 657 OH, LYS 658 CG, ILE 659 HG11, ALA 662 HA, HOH 32 OH2, HOH 37H2, HOH 70 OH2, HOH 107 OH2, HOH 107H2, HOH 170 OH2, HOH 255H1, HOH 255H2, HOH 269H2	0.749	0.735	0.404	0.392
	GFA + SHAP	Cdocker energy, Cdocker interaction energy	0.634	0.635	0.169	0.181

a GFA: genetic function algorithm, SHAP: the SHapley Additive exPlanations.

b Amino acid and water heavy atom contacts are coded according to the protein databank, while hydrogen atoms are coded according to Discovery Studio 4.5. LigScore2, PMF, PMF04, Cdocker energy, Cdocker interaction energy, represent scoring values.

c L20% out: leave 20% out cross-validation for accuracy and Cohen's Kappa.

d Testing: accuracy and Cohen's Kappa determined against the testing set (Table S1 under ESI).

**Fig. 6:**
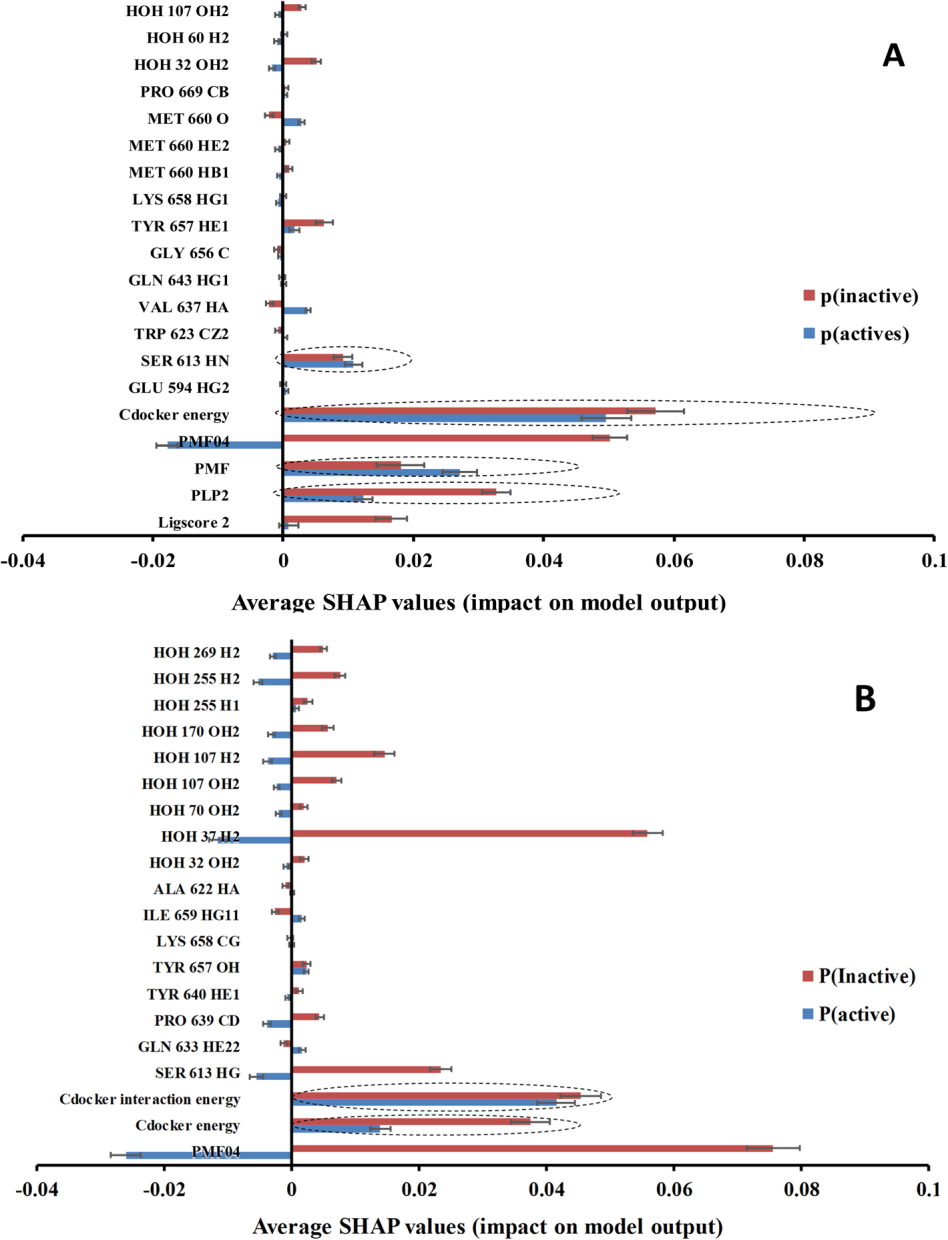
SHAP probability contributions of descriptors emerging in optimal (A): GA-XGBoost and (B): GA-RF models within the testing set compounds. Average probability contribution for “inactive” prediction among inactive compounds are represented with red (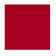) bar, average probability contribution for “active” prediction among active compounds are represented with (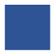) bar. Error bars represent the standard error of the average. SHAP-consistent features are encircled with blue dotted lines.

Clearly from Table 4 and comparison with Table 3, it was possible to successfully reduce the number of features from *ca.* 471 to *ca.* 20 using genetic selection with negligible loss in models' accuracies and Cohen's kappa values highlighting the significance of the shortened lists of descriptors. Nevertheless, it is still hard to infer the role of each descriptor in predicting the bioactivity class of a particular compound based on GFA-ML models in Table 4. For example, it is hard to tell how certain LRCF (*e.g.*, *e.g.*, TYR 640 HE1) contributes to the bioactivity class within the context of GFA-RF ML model (in Table 4). Therefore, we decided to implement Shapley additive explanations (SHAP) to explain the relative contributions of individual descriptors in bioactivity class predictions for each GFA-ML model,^29,55^ as in Fig. 6.

In the context of cheminformatics machine learning, SHAP values enable the identification and prioritization of features that control bioactivity prediction regardless to ML model.^29^ SHAP value of a particular feature for certain compound indicates how much this feature has contributed to the deviation of the prediction of that compound from mean prediction. Each average SHAP value in Fig. 6 was calculated as the mean of SHAP values of the particular descriptor across active or inactive testing compounds.

Interestingly, Fig. 6 shows that only few GFA-selected descriptors have average SHAP probability contributions consistent with corresponding bioactivity categories, *i.e.*, they yielded positive probabilities towards the “active” label classification within active testing compounds and likewise showed positive probability contributions towards “inactive” label within the inactive testing category. These are encircled in Fig. 6. Remarkably, deleting inconsistent descriptors from GA-ML models in Table 4 caused only moderate detrimental effects on the corresponding predictive qualities (see GFA + SHAP selectors in Table 4).

### 3.4 Building pharmacophore models

GFA-selected descriptors of consistent SHAP probabilities were used to select a single docked pose for the most potent inhibitor in the testing list (115, IC_50_ = 136 nM, ESI Table S1†) to be used as template for pharmacophore building. The selected pose is characterized with the highest probability contributions towards the “active” label among other docked poses of 115 based on SHAP-consistent descriptors. It can be reasonably argued that this pose represents the most probable way by which 115 binds within STAT3 binding site according to the considered ML model. Corresponding pharmacophore models are then generated using the Ligand–Receptor Pharmacophore Generation protocol within Discovery Studio (see Section 2.4).

**Fig. 7:**
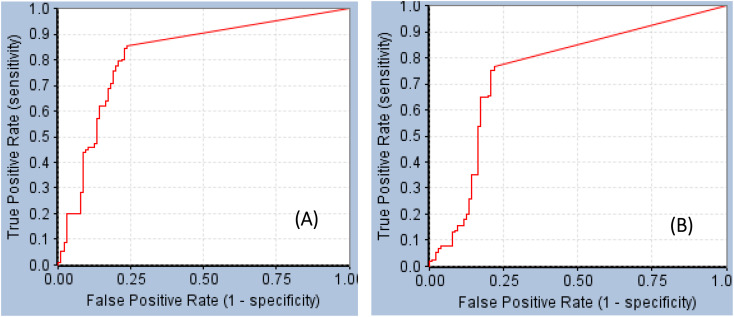
ROC curves of (A) Hypo-1 (AUC = 0.82, sensitivity: 0.85, specificity: 0.76), (B) Hypo-2 (AUC = 0.75, sensitivity: 0.77, specificity: 0.78).^71^

**Fig. 8:**
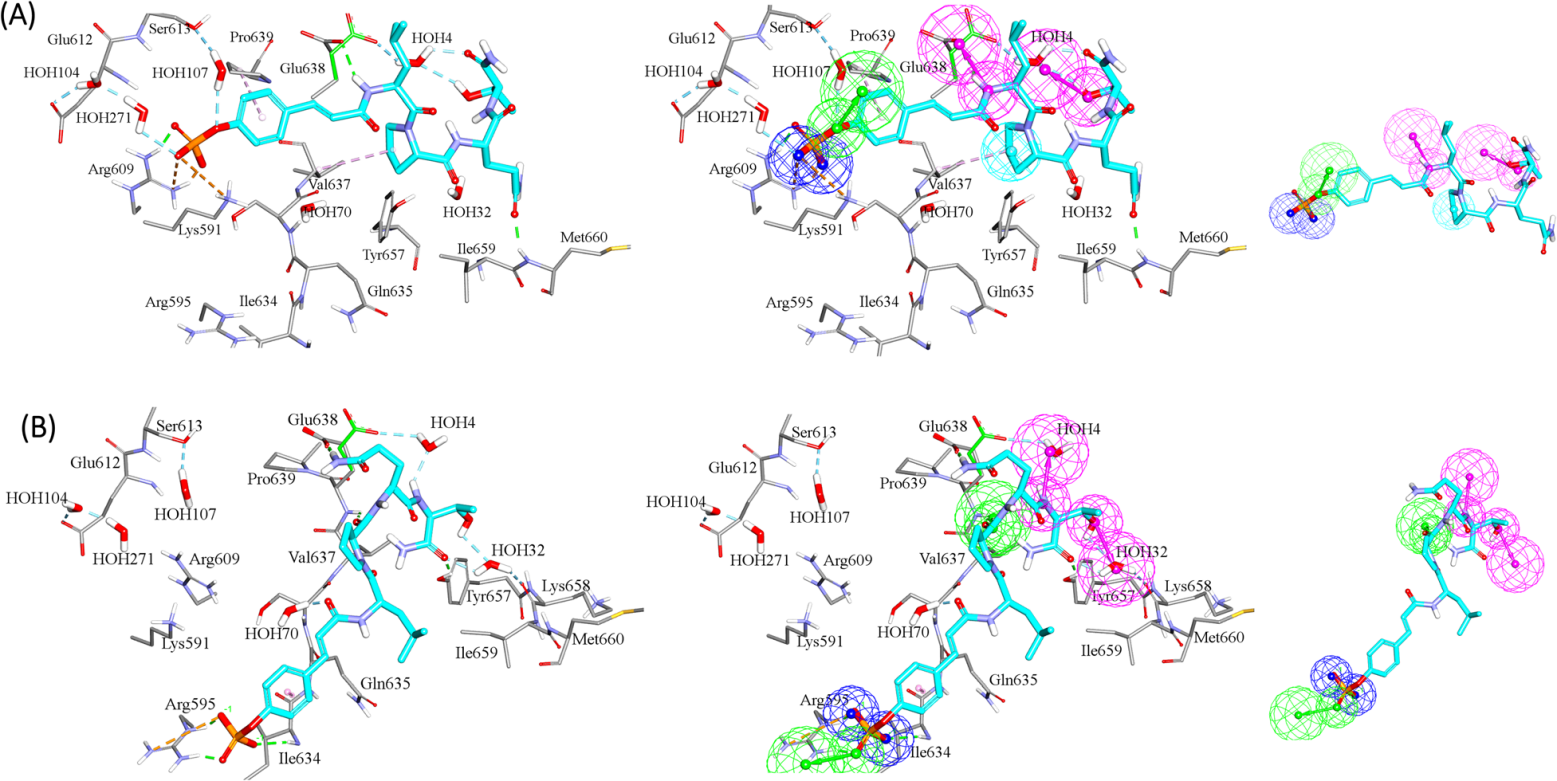
Steps to build pharmacophore models (A) Hypo-1 and (B) Hypo-2 based on SHAP-consistent features identified among GA/XGBoost (for Hypo-1) and GA/RF (for Hypo-2) selected-descriptors. The left images show the docked pose of 115 having the highest “Active” label probability (*i.e.*, among other poses) as contributed by SHAP-consistent features. Hydrogen bonds are shown as green and light blue dotted lines, while hydrophobic interactions are shown as pink dotted lines. The middle images show the pharmacophore hypothesis fitted onto the docked pose. Images to the right show the resulting pharmacophore models. Hydrogen-bond donor (HBD) features are shown as vectored pink spheres, hydrogen bond acceptor (HBA) features are shown as vectored green spheres, hydrophobic (Hbic) features are shown as blue spheres, negative ionizable features are shown as dark blue spheres.

For example, the docked pose in Fig. 8A is of the highest probability contributions by GFA-selected, SHAP-consistent, descriptors within the XGBoost model (Fig. 6A and Table 4, namely, PLP2, PMF, Cdocker energy and SER 613 HN). Hypo-1 pharmacophore (Fig. 8A) was extracted based from this pose *via* the Ligand-Receptor Pharmacophore Generation protocol. Fig. 7A shows its corresponding ROC curve.

Clearly from Fig. 8A, the electrostatic interaction anchoring the docked pose's phosphate group and the guanidine of Arg609 is represented by two overlapping negative ionizable features (NegIon) in Hypo-1. Similarly, the hydrogen bond connecting the terminal hydroxyl of Ser613 to the phosphate ester oxygen atom of 115*via* a bridging water molecule (H_2_O107) is represented in Hypo-1 by hydrogen bond acceptor (HBA) feature. Likewise, the close proximity between the central pyrrolidine ring in the docked ligand pose to the hydrophobic side chain of Val637 indicates mutual hydrophobic attraction that was represented by hydrophobic (Hbic) feature in Hypo-1. Interestingly, although the carboxylic acid side chain of Glu638 is rather flexible and assumes two distinct conformational states in the crystallographic structure of STAT3, it seems that both conformers play critical role in ligand binding: The central amide group of docked 115 (Fig. 8A) is hydrogen-bonded to the carboxylic acid of one of Glu638 conformers, while the other major conformer of this amino acid is hydrogen bonded to the terminal hydroxyl of docked 115*via* bridging water molecule (H_2_O_4_). The two interactions involving Glu638 conformers are represented in Hypo-1 by two hydrogen-bond donor features. This is a rare example where a flexible amino acid residue plays critical role in ligand binding *via* more-than-one conformer.

Fig. 8B shows Hypo-2, which corresponds to the docked pose of 115 having the highest probability contributions by GA-selected and SHAP-consistent descriptors within the RF model (Fig. 6B and Table 4, namely, Cdocker energy and Cdocker interaction energy). These descriptors were used in the same way as in Hypo-1 case to generate Hypo-2 (Fig. 8B). Fig. 7 shows its corresponding ROC curve. Clearly from Fig. 8B, the electrostatic and hydrogen bonding interactions tying the terminal phosphate of 115 with the guanidine side chain of Arg595 are represented in Hypo-2 by two overlapping NegIon and a single HBA features. Meanwhile, the hydrogen-bonding connecting the terminal hydroxyl of docked 115 to the peptidic NH of Lys658 *via* bridging water molecule (H_2_O32) is represented by HBD feature in Hypo-2. Likewise, the hydrogen bonding interactions anchoring the NH and carbonyl oxygen atoms of the ligand's central amides to the carboxylic acid side chain (one of the conformers *via* bridging H_2_O_4_) and peptidic NH of Glu638 are represented by HBD and HBA, respectively, in Hypo-2.

Clearly from the figure the two pharmacophores, Hypo-1 and Hypo-2, represent significantly discrete binding modes.

### 3.5 Comparison with pharmacophores extracted from crystallographic complexes

To further validate our ML-generated pharmacophores we decided to compare their performances with naïve counterparts derived from crystallographic complexes. Towards this end, we implemented the Ligand–Receptor Pharmacophore Generation protocol of Discovery Studio (Version 4.5) to extract pharmacophore models from available STAT3 crystallographic structures complexed with SH2 ligands (PDB codes: 6njs and 6nuq). The resulting models were validated by ROC analysis against the same set of actives and inactives used for validating our ML-generated models Hypo-1 and Hypo-2. Only crystallographic pharmacophores of ROC-AUC exceeding 0.70 were kept for comparison with our ML-based pharmacophores.

**Fig. 9:**
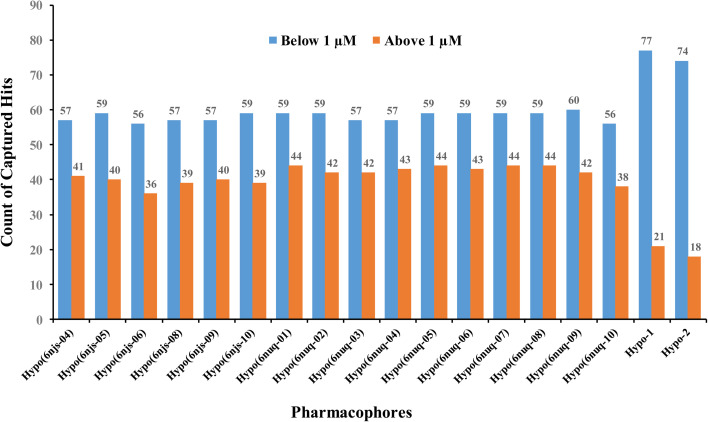
Counts of STAT3 inhibitors of IC_50_ ≥ or < 1.0 μM captured by successful crystallographic pharmacophores (of ROC-AUC > 0.70) compared to our ML-generated pharmacophores.

ESI Fig. S1 and S2† show the ROC curves and pharmacophoric features of successful crystallographic pharmacophores. Although the crystallographic pharmacophores were on par with their ML-based counterparts vis-à-vis ROC performances (ESI Fig. S1†), our ML pharmacophores exhibited much better abilities to classify active STAT3 ligands into potent (IC_50_ < 1.0 μM) and less potent (IC_50_ ≥ 1.0 μM) inhibitors, as in Fig. 9.

This behavior is not unexpected, since the GA-selected and SHAP consistent features used for building pharmacophores Hypo-1 and Hypo-2 were selected by supervised modelling, whereby the bioactivity category dictates which descriptors are significant enough to be selected. These should be able to discriminate highly potent category (IC_50_ < 1.0 μM) from less potent category (IC_50_ ≥ 1.0 μM) within the “active” group. To test this theory, we evaluated the significance of difference between the two categories vis-à-vis descriptors used for building Hypo-1 and Hypo-2. ESI Table S4† summarizes the results. As expected, three out of 5 descriptors used for building Hypo-1 and Hypo-2, namely, PLP2, PMF and Cdocker interaction energy, were significantly different between the two groups (of IC_50_ < 1.0 μM and IC_50_ ≥ 1.0 μM). Moreover, these same descriptors provided the highest SHAP probability contributions for selecting template poses for building Hypo-1 and Hypo-2.

### 3.6 *In silico* screening of the NCI database for new STAT3 inhibitors

The primary use of pharmacophores and related ML models is scaffold hopping, *i.e.*, the identification of new chemotypes with similar biological profiles. Thus, Hypo-1, and Hypo-2 were employed as 3D search queries to screen the NCI list for new STAT3 inhibitors. High ranking hits (according to their fit values^58,72^) were docked, scored (consensus score of at least 1) and RMSD-filtered utilizing the same settings implemented for the training and testing sets. The resulting docked poses were then used to generate corresponding LRCFs in exactly the same manner as in the training and testing sets. Subsequently, the resulting LRCFs and corresponding scoring values were substituted in the best ML models, namely, GFA-Xgboost, GFA-RF, however, using features selected by the combination of SHAP and GFA (GFA + SHAP selector in Table 4) to predict the activity label of each docked pose/conformer. As a result, each screened compound produced a collection of poses that were either labelled as “active” or “inactive”, as in Table 5. The ratio of docked poses/conformers anticipated to be “active” compared to those predicted to be “inactive” forced us to propose a threshold by which to regard a specific screened molecule as being promising or not. Examining the active/inactive ratios within the active compounds within the testing set is the most logical approach to create such a threshold.^19^ It is reasonable to presume that an acceptable threshold for discovering potentially new active hits is the least active-to-inactive ratio among well documented active inhibitors. Table 6 shows the percentages of docked poses of active testing set molecules that were correctly labelled as “active” by the two ML models. Clearly, compound 250 (IC_50_ = 5 μM, Table S1 under ESI†) fulfils the threshold requirements: It exhibits the least predicted active-to-inactive ratio of docked poses among other inhibitors in the testing set for both learners, and therefore, can be used to discriminate actives among screened compounds for the respective ML models. Incidentally, compound 312 failed to achieve sufficient data augmentation as it only produced one docked pose for ML modelling thus it was neglected in the decision related to activity threshold.

**Table 5:** Hit compounds and count of their “active”/“inactive” docked poses as predicted based on GA/SHAP selected features (Table 4)

Hits^a^	NCI Code	Captured By^b^	Predicted number of active and inactive docked poses					
			Xgboost			RF		
			Active poses	Inactive poses	Percent active poses^c^	Active poses	Inactive poses	Percent active poses^c^
317	3590	1,2	183	31	85.5	78	30	72.2
318	20 261	1	40	77	34.2	ND	ND	ND
319	59 407	1	205	34	85.8	ND	ND	ND
320	65 832	1	65	56	53.7	ND	ND	ND
321	745 104	1,2	3	3	50.0	5	1	33.3
322	72 868	1	56	45	55.4	ND	ND	ND
323	77 028	1	52	64	44.8	ND	ND	ND
324	77 029	1	47	64	42.3	ND	ND	ND
325	82 523	1	152	20	88.4	ND	ND	ND
326	98 711	1	48	71	40.3	ND	ND	ND
327	100 791	1	70	38	64.8	ND	ND	ND
328	107 137	1	102	41	71.3	ND	ND	ND
329	107 139	2	ND	ND	ND	45	11	80.4
330	267 431	1,2	40	48	45.5	48	37	56.5
331	289 523	1	76	28	73.1	ND	ND	ND
332	338 310	2	ND	ND	ND	129	34	79.1
333	341 076	1,2	217	39	84.8	189	29	86.7
334	341 077	1,2	209	41	83.6	180	37	82.9
335	363 007	2	ND	ND	ND	119	42	73.9
336	372 667	1,2	158	26	85.9	131	40	76.6
337	373 233	1	53	55	49.1	ND	ND	ND
338	380 962	1	45	33	57.7	ND	ND	ND
339	645 793	2	ND	ND	ND	40	23	63.5
340	651 016	2	ND	ND	ND	52	33	61.2
341	669 269	1,2	135	54	71.4	124	33	79.0
342	722 969	2	ND	ND	ND	51	20	71.8

a Chemical structures are shown in Fig. S3.

b 1 represents XGboost-GFA model or Hypo-1, 2 represents RF-GFA model or Hypo-2.

c Determined by dividing the number of active poses by the total number of poses (active + inactive). ND: not determined.

**Table 6:** Predicted active and inactive docked poses for testing set active compounds based on GA/SHAP-selected features

Compounds^a^	Predicted number of active and inactive docked poses					
	Xgboost			RF		
	Active poses	Inactive poses	% Active poses^b^	Active poses	Inactive poses	% Active poses^b^
10	105	14	88.2	98	21	82.4
22	113	21	84.3	99	35	73.9
34	86	22	79.6	88	20	81.5
49	76	20	79.2	73	23	76.0
54	60	29	67.4	57	32	64.0
66	76	20	79.2	75	21	78.1
72	98	23	81.0	87	34	71.9
78	77	2	97.5	65	14	82.3
107	54	17	76.1	58	13	81.7
113	57	6	90.5	56	7	88.9
115	90	2	97.9	73	19	79.3
116	67	22	75.3	65	24	73.0
126	97	2	98.0	80	19	80.8
127	66	1	98.5	55	12	82.1
133	51	2	96.2	41	12	77.4
135	65	0	100	56	9	86.2
146	82	5	94.3	73	14	83.9
148	54	3	94.7	45	12	78.9
156	90	7	92.8	85	12	87.6
162	62	5	92.5	59	8	88.1
250^c^	10	32	23.8	24	18	57.1
312	0	1	0	1	0	100

a Compounds' numbers and bioactivities are as in Table S1.

b Determined by dividing the number of poses labeled as “active” by the total number of poses (labeled as “active” and “inactive”).

c The percent active poses of this compound (IC_50_ = 5000 nM) was used as threshold to classify screened compounds into potential active and inactive STAT3 inhibitors in both GA-RF and GA-XGboost models.

Being above the proposed activity threshold of either ML models, it can be argued that hits 317–342 (Table 5) have promising potential as active STAT3 inhibitors. Accordingly, they were acquired from the national cancer institute (NCI) for *in vitro* evaluation. ESI Fig. S3† shows the chemical structures of the evaluated hits.

### 3.7 *In vitro* bioassay of captured hits

To study the anti-STAT3 inhibitory effects of captured hits we decided to use pyrimethamine as role model. Pyrimethamine, a well-known antimicrobial and antimalarial agent,^73^ is reported to be indirect selective STAT3 inhibitor^65^ that acts by blocking dihydrofolate reductase (DHFR) enzyme.^63^ Pyrimethamine is currently investigated as potential clinically useful STAT3 inhibitor.^74^ Therefore, we decided to assess the cytotoxic profiles of pyrimethamine (at 10 μM) against 10 cell lines (available within our stock) to identify cells that rely on STAT3 for their survival. The scanned cell lines were normal fibroblasts, HEK-293, 3T3, PANC1, DU145, U87, MDA-MB-231, A549, doxorubicin resistant and sensitive MCF7. Eventually, five cell lines were selected: HEK-239, MCF-7, U87, MDA-MB-231 and Fibroblasts. Of them, normal fibroblasts were found to be least susceptible to pyrimethamine with viability exceeding 90% (Table 7), while HEK293 cells were rather sensitive with viability ≲ 50%. On the other hand, MDA-MB231, U87 and MCF-7 cells were found to exhibit moderate sensitivities to pyrimethamine with viability range from 70–80% (Table 7).

**Table 7:** Inhibition percentages of NCI hits against 5 different cell lines at 10 μM as determined by MTT assay. Each measurement represents average of 4 trials

Compound	HEK-293	MCF-7	U87	MDA-MB-231	Fibroblasts
317	0	7	3	0	0
318	8	14	4	0	0
319	**60**	**46**	**21**	**36**	**7**
320	26	8	23	10	0
321	27	9	3	0	4
322	11	22	7	23	9
323	2	13	13	2	0
324	0	13	7	6	0
325	38	13	38	8	30
326	0	3	1	15	10
327	3	3	0	0	11
328	0	16	0	0	11
329	10	15	0	0	12
330	6	17	0	0	14
331	0	24	0	2	25
332	14	20	10	3	0
333	**53**	**24**	**3**	**24**	**1**
334	39	37	0	1	14
335	3	21	9	9	18
336	11	25	0	0	2
337	37	0	0	21	11
338	0	12	0	0	13
339	0	0	16	0	7
340	31	3	5	0	2
341	42	44	32	21	7
342	**53**	**39**	**18**	**20**	**23**
Pyrimethamine^a^	53	31	19	20	7
Stattic^a^	88	92	90	87	81

a Standard STAT3 inhibitors.

**Fig. 10:**
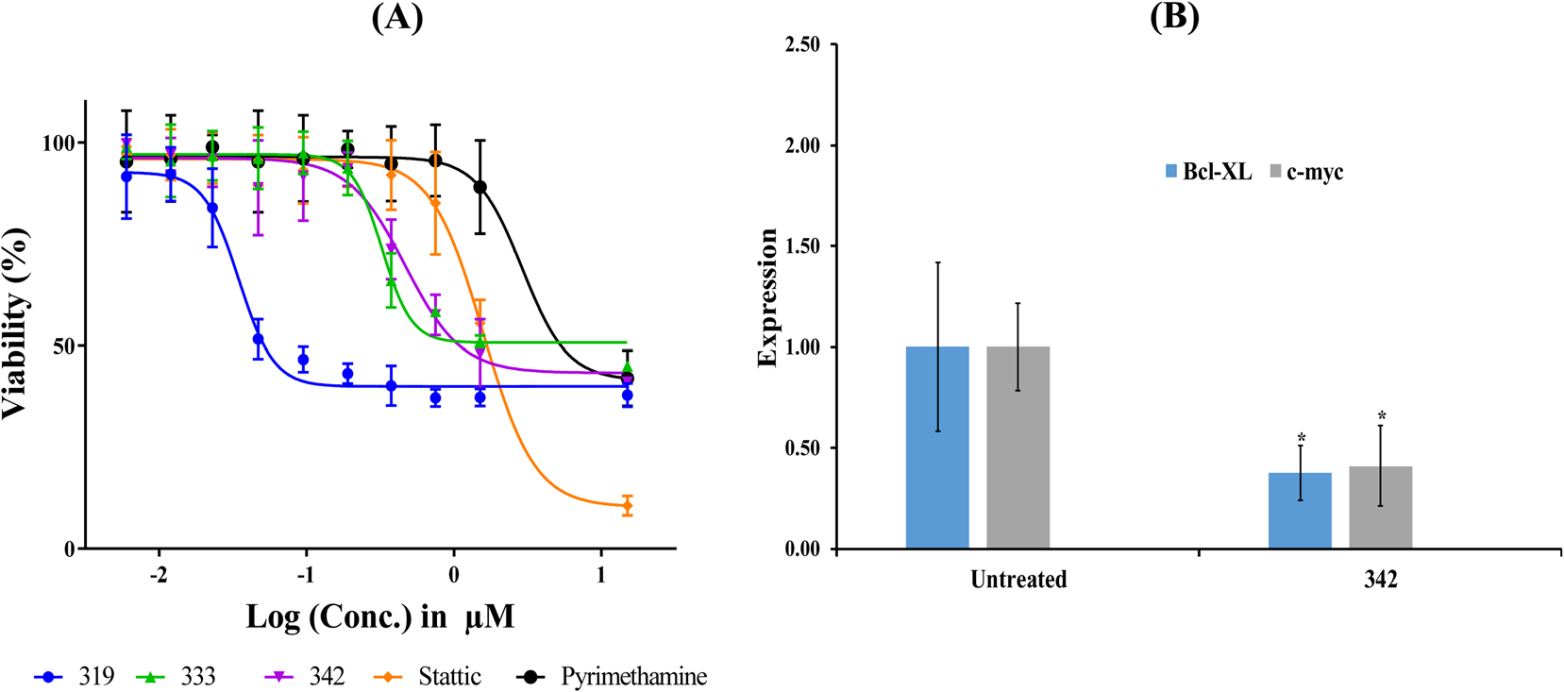
Bioactivity profiles of hit 342. (A) Dose-cellular viability curves of hit 342 compared to stattic and pyrimethamine against HEK 293 cells. (B) Expression of Bcl-xl and c-Myc genes following exposure to 342 at concentration corresponding to anticancer cytotoxic IC_50_ (see Table 8). Gene expression values were calculated using ΔΔ*C*_t_ method against housekeeping genes (actin-β and 18srRNA). *p < 0.05.

Table 7 shows the cytotoxic profiles of captured hits compared to the standard STAT3 inhibitors pyrimethamine and stattic.^14^ Clearly, hits 319, 333 and 342 (structures in ESI Fig. S3†) mimicked the cytotoxic profile of pyrimethamine prompting us to further pursue their IC_50_ values against the HEK293 (selected because it is the most STAT3-sensitive cell line). Table 8 and Fig. 10A show their IC_50_ values, Hill Slopes and dose–response correlation *r*^2^ values against HEK293 cells. Interestingly, stattic caused lower cellular viabilities at higher concentrations (≥15 μM) compared to all three hits and pyrimethamine, which had their curves plateaued at approximately 40% viability regardless to their escalating concentrations. We propose this behaviour to be due to the fact that stattic exerts nonselective inhibitory profiles against a plethora of targets beside STAT3.^75,76^

**Table 8:** IC_50_ values (μM) for the most potent NCI hits on HEK 293 cell line

Hit	IC_50_^a^ (μM)	Hill lope	*r* ^2^ b
319	3.50 × 10^−2^ (±0.004)	3.7	0.93
333	6.74 (±3.55)	0.8	0.95
342	0.44 (±0.10)	2.8	0.91
Stattic	1.57 (±0.17)	2.5	0.96
Pyrimethamine	5.12 (±1.19)	1.8	0.86

a Each value represents the average of 7 trials, values in brackets represent standard deviation of measurements.

b The goodness of fit correlation coefficient of the dose–response curve.

Due to the fact that both 319 and 333 were reported to potently inhibit DHFR enzyme (IC_50_ values in nanomolar range)^77,78^ their STAT3 inhibition should be at least partially indirect,^63,79^ forcing us to exclude them from further evaluation. On the other hand, 342 has been reported to be totally devoid of DHFR inhibitory effects,^80^ and therefore, its bioactivity is attributable solely to direct binding to STAT3 SH2 domain.

The fact that cytotoxicity cannot be considered as unequivocal evidence of STAT3 inhibition; we opted for additional investigation using quantitative polymerase chain reaction (qPCR) to monitor the effect of 342 on the expression of c-Myc and Bcl-xl genes, both of which are downstream of STAT3. c-Myc and Bcl-xl are key regulators in cellular proliferation^81^ and evasion of apoptosis,^82^ respectively.

Fig. 10 shows the effect of 342, at its anti-HEK293 IC_50_ concentration (Table 8), on the expression of c-Myc and Bcl-xl. Clearly, 342 caused significant suppression of c-Myc and Bcl-xl. These results provide unequivocal evidence on the potent and statistically significant inhibitory effect of 342 against STAT3 at submicromolar levels (*i.e.*, 440 nM).

**Fig. 11:**
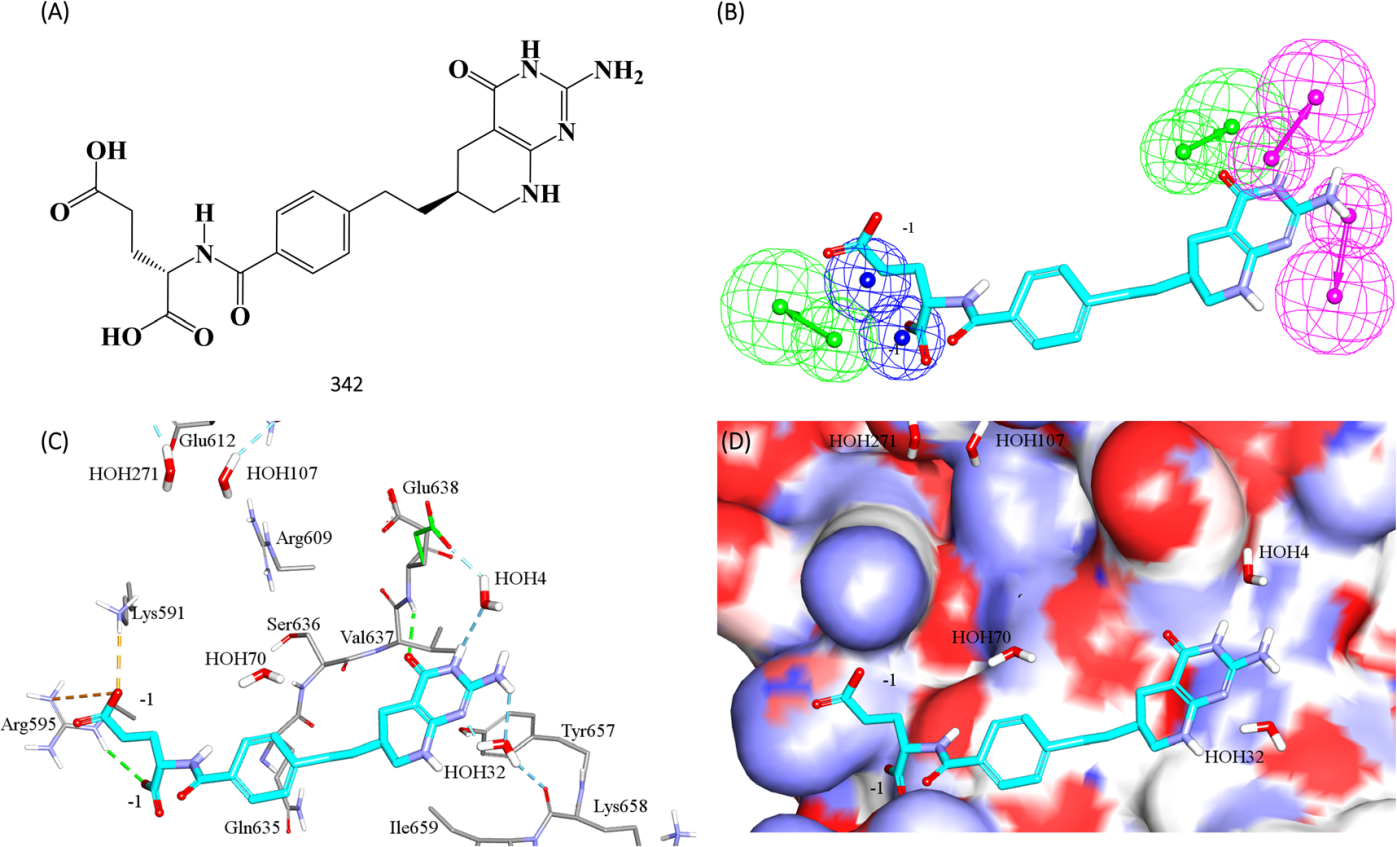
Hit 342 mapped against its capturing pharmacophore (A) structure of 342 (B) 342 fitted against Hypo-2, (C) 342 docked into STAT3 (PDB code: 6njs), (D) 342 docked into STAT3 with binding site covered with Connolly's surface.

Fig. 11 shows 342 and how it fits its corresponding capturing pharmacophore (Hypo-2) and how it docks into the binding pocket of STAT3 SH2 domain.

**Fig. 12:**
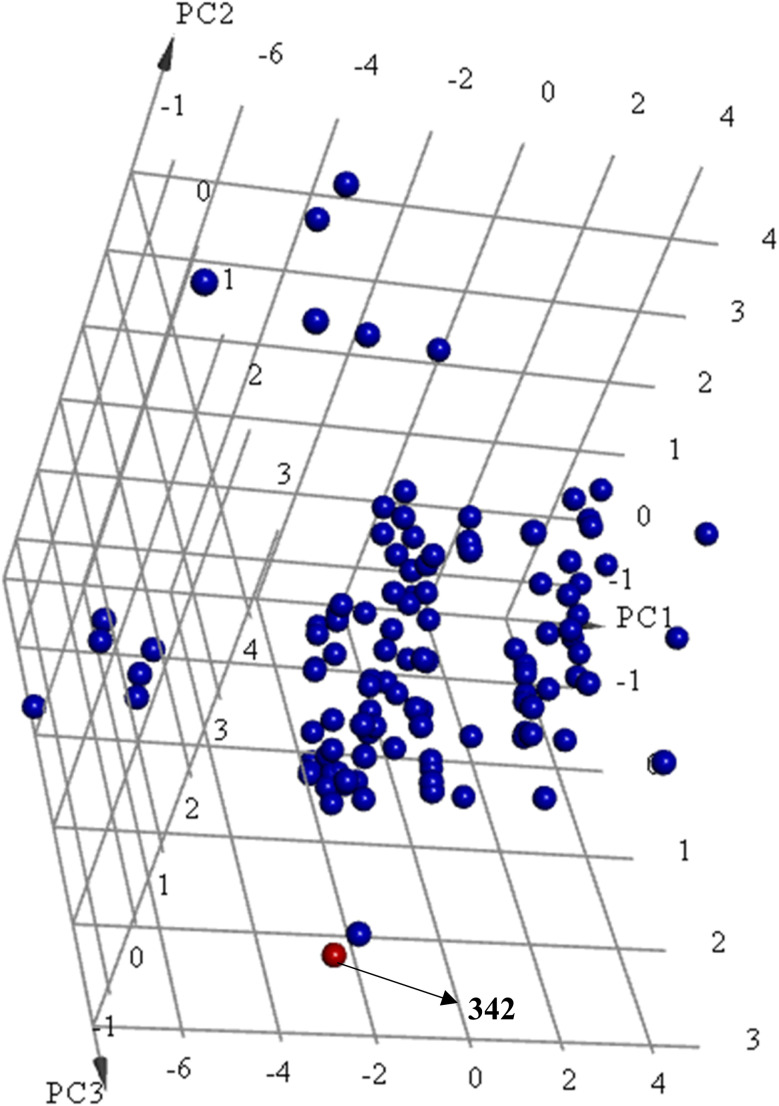
Principal component analysis showing the relative distribution of captured active hit 342 (structure in Fig. 11 and bioactivity in Table 8, red spheres 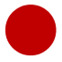) compared to modeled active compounds (IC_50_ ≤ 5.0 μM, ESI Table S1,† blue spheres 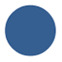). The top three principal components calculated for modeled compounds and captured hits are based on 11 descriptors (*i.e.*, log(*P*), molecular weight, hydrogen bond donors and acceptors, rotatable bonds, number of atoms, number of rings, number of aromatic rings, molecular surface area, molecular polar surface area and molecular fractional polar surface area). Active hits are indicated in the figure with arrows.

Principal component analysis (PCA) (Fig. 12) shows 342 to be significantly different chemotype compared to known potent STAT3 SH2 blockers (IC_50_ ≤ 5 μM) albeit drug-like and satisfies Lipinski's^83^ and Veber's^84^ rules.

## 4. Conclusion

In conclusion, new STAT3 inhibitory lead of potent anti-STAT3 IC_50_ and novel chemotype was discovered using data augmentation algorithm based on computational sequence of docking, scoring, ligand-receptor contacts fingerprints. Optimal ML models and associated descriptors were translated into pharmacophore models. The resulting pharmacophores were validated by receiver operating characteristic (ROC) curve analysis and used as virtual search queries to screen the NCI database for promising STAT3 inhibitors.

## Conflicts of interest

There are no conflicts of interest to declare.

## Supplementary Material

RA-013-D2RA07007C-s001
