# Supplementary material for: Discovery of new STAT3 inhibitors as anticancer agents using ligand-receptor contact fingerprints and docking-augmented machine learning
Source: RSC Adv. 2023 Feb 3;13(7):4623–40. doi: 10.1039/d2ra07007c (PMC9896621; doi:10.1039/d2ra07007c)
Supplement: RA-013-D2RA07007C-s001 [file RA-013-D2RA07007C-s001.pdf]

Electronic Supplementary Material (ESI) for RSC Advances.
This journal is © The Royal Society of Chemistry 2023

Supporting Information

Discovery of New STAT3 Inhibitors as Anticancer Agents Using Docking-Based Ligand-Receptor Contacts Fingerprints and Bioactivity - Classifying Machine Learning

Nour Jamal Jaradat[1], Walhan Alshaer [2], Mamon Hatmal[3], Mutasem Omar Taha[1]*

[1]Department of Pharmaceutical Sciences, Faculty of Pharmacy, University of Jordan, Amman 11492, Jordan.

[2]Cell Therapy Center, the University of Jordan, Amman 11942, Jordan.

[3]Department of Medical Laboratory Sciences, Faculty of Applied Medical Sciences, The Hashemite University, P.O. Box 330127, Zarqa 13133, Jordan

*Corresponding Author.,

Telephone: 00962 6 5355000 ext. 23305.

Fax: 00962 6 5339649.

Email: mutasem@ju.edu.jo

Table of Contents

**Pages 2-8**	**SM1-SM3**
**Pages 9- 28**	**Table S1**
**Page 28**	**Table S2**
**Page 29**	**Table S3**
**Page 30**	**Table S4**
**Page 30**	**Table S5**
**Page 31**	**Figure S1**
**Page 32**	**Figure S2**
**Pages 33-37**	**Figure S3**

SM1. Docking

LibDock

The site-feature docking algorithm (LibDock) docks ligands, after the removal of their hydrogen atoms, into the active site guided by the binding hotspots. The ligands' conformations are aligned to polar and nonpolar receptor interaction sites, i.e., hotspots[31, 32]. The following LibDock parameters were implemented in the current study: CAT-CONFIRM module of Discovery Studio 4.5 was utilized to produce a maximum of 255 conformers (not exceeding an energy threshold of 20 kcal/mol from the most stable conformer) for each ligand employing "CAESAR" conformation generation option. A binding site sphere of 12.5 Å radius surrounding the centre of the copied co-crystallized ligand (PDB code: KQV) from the STAT3 crystallographic structure (PDB code: 6NJS) was utilized to locate the binding site. The number of binding site hotspots (polar and apolar) was set to 100. The ligand-to-hotspots matching RMSD tolerance value was set to 0.25 Å. The maximum number of poses saved for each ligand during hotspots matching before final pose minimization = 100. Maximum number of poses to be saved for each ligand in the binding pocket = 100. Minimum LibDock score (poses below this score are not reported) = 100. Maximum number of rigid body minimization steps during the final pose optimization (using BFGS method) = 50. Maximum number of steric clashes allowed before the pose-hotspot alignment is terminated (specified as a fraction of the heavy atom count) = 0.1. Maximum value for nonpolar solvent accessible surface area for a particular pose to be reported as successful = 15.0 $Å^2$. Maximum value for polar solvent accessible solvent area for a particular pose to be reported as successful = 5.0 $Å^2$. Docked poses/conformers were finally energy-minimized using the SMART minimizer option in LibDock. This option performs two minimization steps: Initial 1000 steps of Steepest Descent with an RMS gradient tolerance of three (kcal/mol/Å), followed by a maximum of 1000 steps of

Conjugate Gradient minimization with an RMS gradient tolerance of 0.001 (kcal/mol/Å) and minimization energy change of 0.0 kcal.

CDOCKER

CDOCKER is a CHARMm-based simulated annealing/molecular dynamics methodology that applies rigid receptor for docking [34]. The following CDOCKER parameters were implemented in the current project. A binding site sphere of 12.5 Å radius surrounding the centre of the co-crystallized ligand (PDB code: 6NJS; PDB code of ligand: KQV) was implemented. Starting ligands' conformers were energy-minimized then heated to 1000 K over 1000 molecular dynamics steps to generate 10 starting random conformations for each ligand. Each random conformer was rotated 10 times within the binding pocket for consequent energy refinement. The Van der Waals energies of the resulting conformers/poses were evaluated and those of ≥ 300 kcal/mol were discarded. Surviving conformers/poses were exposed to a cycle of simulated annealing over 2000 heating steps to targeted temperature of 700 K followed by 5000 cooling steps to targeted temperature of 300 K. The docked poses were energy minimized to a final minimization gradient tolerance zero Kcal/mol/Å. Top 100 poses were saved for subsequent scoring.

LigandFit

LigandFit employs Monte-Carlo techniques to create ligand conformations and docks them into the active site utilizing shape-based initial docking[33]. The following LigandFit settings were implemented in the current research. A binding site composed of 8434 grid points (grid spacing of 0.5Å in the X., Y and Z dimensions) surrounding the co-crystallized ligand within STAT3 (PDB code: 6NJS., PDB code of ligand: KQV) was used. Monte Carlo search parameters are as follows:

Number of trials = 30000 and search step for torsions with polar hydrogens = 30.0. The RMS threshold for ligand-to-binding site shape match was set to 2.5 Å., assuming a maximum of three binding site partitions. Interaction energy parameters: The interaction energies were assessed using CFF force field (version 1.02) with a non-bonded cutoff distance of 10.0 Å and distance dependent dielectric. An energy grid extending 3.0 Å from the binding site was implemented. The interaction energy was estimated by a trilinear interpolation value using soft potential energy approximations. Rigid body ligand minimization parameters: 40 steepest descend followed by 80 BFGS minimization iterations were applied to every orientation of the docked ligand. The maximum number of retained poses is 100. Each docked pose/conformer was energy-minimized using the SMART minimizer option in LigandFit., which executes two minimization steps: Initial 1000 steps of Steepest Descent with an RMS gradient tolerance of 3 (kcal/mol/Å)., followed by a maximum of 1000 steps of Conjugate Gradient minimization with an RMS gradient tolerance of 0.001 (kcal/mol/Å) and minimization energy change of 0.0 kcal.

SM2. Machine Learning

Random Forests (RF)

RF is a multipurpose ML strategy for classification based on ensemble of Decision Trees (DTs). Each tree predicts a classification independently and "votes" for the related class. Most of the votes decide the overall RF predictions[21]. We implemented RF Learner node within KNIME Analytics Platform (Version 4.3.3) with the following settings: Splitting criterion is Information Gain Ratio (which normalizes the standard information gain by the split entropy to overcome any unfair preference for nominal splits with many child nodes)., Number of trees = 100. No limitations were imposed on the number of levels or minimum node size.

eXtreme Gradient Boosting (XGBoost)

eXtreme Gradient Boosting (XGBoost., or XGB) relies on the ensemble of weak decision tree (DT) type models to create boosted., DT-type models. This system includes a novel tree learning algorithm., a theoretically justified weighted quantile sketch process with parallel and distributed computing[22]. We implemented the XGBoost Learner node within KNIME Analytics Platform (Version 4.3.3) with the following settings: Tree booster was implemented with depthwise grow policy., boosting rounds = 100., Eta = 0.3., Gamma = 0., maximum depth = 6., minimum child weight = 1., maximum delta step = 0., subsampling rate = 1., column sampling rate by tree = 1., column sampling rate by level = 1., lambda = 1., Alpha = 0., sketch epsilon =0.03., scaled positive weight = 1. Maximum number of bins = 256., Sample type (uniform)., Normalize type (tree)., and Dropout rate = 0.

Naïve Bayes

Naïve Bayes (NB) is a simple classifier whereby class labels are predicted and assigned to external observations based on vectors of descriptors for some limited set of training observations. NB classifier presumes each descriptor to contribute independently to the probability that certain observation (i.e., docked pose) belongs to a particular class (i.e., active, intermediate, or inactive)[23]. The probability of certain observation to belong to certain class is the multiplication of the individual probabilities of that class within each individual descriptor[24].

Probabilistic Neural Network PNN

It is a feed forward neural network used for classification and pattern recognition. PNN is formed by replacing the sigmoid activation function with an exponential function that can compute nonlinear decision boundaries approaching the Bayes optimal. A PNN classifier, having four layers of neural network, can be used to map any input pattern to any number of classification[25, 26]. PNN models were built using the PNN learner node default settings within KNIME Analytics Platform (Version 4.3.3).

k-Nearest Neighbors (kNN)

The kNN classifier depends on a distance learning methodology that calculates the activity value of an unknown member based on the bioactivities of a certain number (k) of nearest neighbors (kNNs) in the training set. In this classifier., the similarity is measured by a distance metric[17]. We implemented kNN Learner node within KNIME Analytics Platform (Version 4.3.3). The value of k was scanned from 3 to 5. The runs were repeated to include all possible combinations (i.e., of k) resulting from allowing the influence of neighbors to be either distance-dependent or distance-independent.

Multilayer perceptron (MLP)

MLP which is a modification of the standard linear perceptron, has a neural network architecture consisting of a layer with several nodes., where each node connects to a subsequent node in another layer. The concept of NN is that each input into the neuron has its own weight., that is adjusted to train the network. In between the inputs and the output layer., there may be several hidden layers[27]. We implemented the MLP learner node in its default settings within the KNIME Analytics Platform (Version 4.3.3).

SM3. Biological evaluation of the captured hits

Cell viability assay (MTT) and IC_{50} determination

Rapid colorimetric test based on mitochondrial dehydrogenase enzymes' capacity to transform 3,-4,5 dimethylthiazol -2,5 diphenyl tetrazolium bromide (MTT) into a purple formazan precipitate. A multi-well plate reader is used to measure the optical density after the formazan crystals have been dissolved. Due to its simplicity and suitability for automation, MTT has thus emerged as the preferred technique for first drug screening in cell lines to assess cell viability[54].

To determine the IC_{50} of NCI hits for cells under study., first; an MTT assay was performed. Approximately 8×10^3 cells per well were seeded into a 96 well plate (Corning., USA) and treated with different concentrations of NCI ligands., Both treated and control cells were incubated at 37 °C in a 5% CO_2 incubator for 72 h after which the old media was aspirated and the MTT assay salt (Bioworld., USA) in 100 µl of fresh media was added to each well. Following that., plates were incubated at 37 °C for 3 h., and then 50 µl of solubilization solution (DMSO) was added to each well. The absorbance of the solution was measured at 570 nm using Glomax plate reader (Promega., USA) to determine cells' viability.

Quantitative Polymerase Chain Reaction (qPCR) analysis

qPCR analysis was performed to determine the expression intensities of target genes (c-Myc and Bcl-xl) at mRNA levels. Cells were lysed by Trizol-hybrid method for RNA extraction using miniRNeasy kit (Qiagen, USA). The extracted RNA was quantified by Nanodrop (Thermofisher, USA). To synthesize cDNA, 0.5 µg total RNA was reverse transcribed using PrimeScript RT Master Mix (Cat No. RR036A, Takara, China) using T100™ Thermal cycler PCR instrument (BioRad, USA). Primers were designed using Primer-BLAST (RRID:SCR_003095) and obtained from IDT (USA) (Table S2).

The qPCR reaction mix was prepared by mixing 25 ng of cDNA with 200 nM of gene-specific forward and reverse primers (IDT), 7.2 µL of nuclease-free water, and 10 µL of SYBR Premix Ex Taq II (Tli RNaseH Plus) (Cat No. RR820L, Takara, China). The amplification was performed on CFX96 C1000 Touch thermal cycler (BioRad, USA) with the following temperature setting: (i) 95 ° C for 3 minutes, (ii) 40 cycles 95 ° C for 5 seconds and 61 ° C for 30 seconds. 18srRNA and actin-β were used as housekeeping reference genes. Each sample was examined in triplicate, and

a mean value was calculated. Data were analyzed according to ΔΔCt method using CFX Maestro™ Software - Bio-Rad.

Table S1. Chemical structures of modeled compounds in SMILE formats and their reported bioactivities

Compound	Chembl ID	SMILES	IC_{50} (nM)
**1**	CHEMBL156448	CC(=O)N[C@@H](Cc1ccc(OP(=O)(O)O)cc1)C(=O)N[C@H](C(=O)N[C@@H](CC(C)C)C(=O)N[C@@H](CCC(N)=O)C(=O)NCC(=O)N[C@@H](CC(=O)O)C(N)=O)C(C)C	8000
**2**	CHEMBL405322	CC(=O)N[C@@H](Cc1ccc(C(=O)O)cc1)C(=O)N[C@@H](CC(C)C)C(=O)N1CCC[C@@H]1C(=O)N[C@@H](CCC(N)=O)C(=O)N[C@H](C(=O)N[C@H](C(N)=O)C(C)C)[C@@H](C)O	6000
**3**	CHEMBL160849	CC(=O)N[C@@H](Cc1ccc(CP(=O)(O)O)cc1)C(=O)N[C@@H](CC(C)C)C(=O)N1CCC[C@@H]1C(=O)N[C@@H](CCC(N)=O)C(=O)N[C@H](C(=O)N[C@H](C(N)=O)C(C)C)[C@@H](C)O	8000
**4**	CHEMBL346370	CC(=O)N[C@@H](Cc1ccc(OP(=O)(O)O)cc1)C(=O)N[C@@H](C)C(=O)N1CCC[C@@H]1C(=O)N[C@@H](CCC(N)=O)C(=O)N[C@H](C(=O)N[C@H](C(N)=O)C(C)C)[C@@H](C)O	1600
**5**	CHEMBL160730	CC(=O)N[C@@H](Cc1ccc(OP(=O)(O)O)cc1)C(=O)N[C@@H](CCC(N)=O)C(=O)N1CCC[C@@H]1C(=O)N[C@@H](CCC(N)=O)C(=O)N[C@@H](C)C(=O)N[C@@H](CCCCN)C(N)=O	6000
**6**[a]	CHEMBL156168	CC[C@H](C)[C@H](NC(=O)[C@H](Cc1ccc(OP(=O)(O)O)cc1)NC(C)=O)C(=O)N[C@@H](CC(N)=O)C(=O)N[C@@H](CCC(N)=O)C(=O)N[C@@H](CO)C(=O)N[C@H](C(N)=O)C(C)C	30000
**7**	CHEMBL157314	CC(=O)N[C@H](Cc1ccc(OP(=O)(O)O)cc1)C(=O)N[C@H](CC(C)C)C(=O)N1CCC[C@H]1C(=O)N[C@@H](CCC(=O)O)C(=O)N[C@H](C(=O)N[C@H](C(N)=O)C(C)C)[C@@H](C)O	6000
**8**	CHEMBL346706	CC(=O)N[C@@H](Cc1ccc(OP(=O)(O)O)cc1)C(=O)N[C@@H](CO)C(=O)N[C@@H](CC(=O)O)C(=O)NCC(=O)N[C@@H](CC(N)=O)C(=O)N[C@@H](Cc1ccccc1)C(N)=O	150000
**9**	CHEMBL156407	CC(=O)N[C@H](Cc1ccc(OP(=O)(O)O)cc1)C(=O)N[C@H](CC(C)C)C(=O)N1CCC[C@H]1C(=O)N[C@@H](CCC(N)=O)C(=O)N[C@@H](C)C(=O)N[C@H](C(N)=O)C(C)C	800
**10**[a]	CHEMBL160658	CC(=O)N[C@@H](Cc1ccc(OP(=O)(O)O)cc1)C(=O)N[C@@H](CC(C)C)C(=O)N1CCC[C@H]1C(=O)N[C@H](CCC(N)=O)C(N)=O	1000
**11**	CHEMBL156046	CC(=O)N[C@@H](Cc1ccc(OP(=O)(O)O)cc1)C(=O)N[C@@H](CC(C)C)C(=O)NC(C)C(=O)N[C@@H](CCC(N)=O)C(=O)N[C@H](C(=O)N[C@H](C(N)=O)C(C)C)[C@@H](C)O	4000

**12**	CHEMBL348612	CC(=O)N[C@@H](Cc1ccc(OP(=O)(O)O)cc1)C(=O)N[C@@H](CC(C)C)C(=O)N[C@@H](CCCN=C(N)N)C(=O)N[C@H](CS)C(=O)N[C@H](CN[C@@H](CO)C(N)=O)CC(=O)O	80000
**13**	CHEMBL2371210	CC(=O)N[C@@H](Cc1ccc(OP(=O)(O)O)cc1)C(=O)N[C@@H](CO)C(=O)N[C@H](C(=O)N[C@H](C(=O)N[C@H](C(=O)N[C@@H](Cc1c[nH]cn1)C(N)=O)C(C)C)C(C)C)[C@@H](C)O	>150000
**15**	CHEMBL348880	CC(=O)N[C@@H](Cc1ccc(OP(=O)(O)O)cc1)C(=O)N[C@@H](CC(C)C)C(=O)N1CCC[C@@H]1C(N)=O	6000
**16**	CHEMBL2373425	CC(=O)N[C@@H](Cc1ccc(OP(=O)(O)O)cc1)C(=O)N[C@@H](CCCN=C(N)N)C(=O)N[C@@H](Cc1c[nH]cn1)C(=O)N[C@@H](CCC(N)=O)C(=O)N[C@H](C(=O)N1CCC[C@H]1C(N)=O)C(C)C	6000
**17**	CHEMBL423775	CC(=O)N[C@@H](Cc1ccc(OP(=O)(O)O)cc1)C(=O)N[C@@H](Cc1ccccc1)C(=O)N[C@@H](CCCCN)C(=O)N[C@@H](CCC(N)=O)C(=O)N[C@@H](CC(N)=O)C(=O)N[C@@H](CS)C(N)=O	60000
**18[a]**	CHEMBL348181	CC(=O)N[C@@H](Cc1ccc(OP(=O)(O)O)cc1)C(=O)N[C@@H](CCC(N)=O)C(=O)N[C@@H](CCCCN)C(=O)N[C@@H](CCC(N)=O)C(=O)N[C@H](C(=O)N[C@@H](CCCN=C(N)N)C(N)=O)[C@@H](C)O	8000
**19[a]**	CHEMBL156465	CC(=O)N[C@H](Cc1ccc(OP(=O)(O)O)cc1)C(=O)N[C@H](CC(C)C)C(=O)N1CCC[C@H]1C(=O)N[C@@H](C)C(=O)N[C@H](C(=O)N[C@H](C(N)=O)C(C)C)[C@@H](C)O	6000
**20**	CHEMBL348182	CC(=O)N[C@@H](Cc1ccc(OP(=O)(O)O)cc1)C(=O)N[C@@H](Cc1c[nH]cn1)C(=O)N[C@@H](CC(N)=O)C(=O)N[C@@H](CCC(N)=O)C(=O)N1CCC[C@@H]1C(=O)N[C@@H](CC(C)C)C(N)=O	150000
**21**	CHEMBL158446	CC(=O)N[C@@H](Cc1ccc(-c2nn[nH]n2)cc1)C(=O)N[C@@H](CC(C)C)C(=O)N1CCC[C@@H]1C(=O)N[C@@H](CCC(N)=O)C(=O)N[C@H](C(=O)N[C@H](C(N)=O)C(C)C)[C@@H](C)O	6000
**22[a]**	CHEMBL345419	CSCC[C@H](NC(=O)[C@H](C)NC(=O)[C@H](CCC(N)=O)NC(=O)[C@@H]1CCCN1C(=O)[C@@H](CCCN=C(N)N)NC(=O)[C@@H](Cc1ccc(OP(=O)(O)O)cc1)NC(C)=O)C(N)=O	1000
**23**	CHEMBL160353	CC(=O)N[C@@H](Cc1ccc(OP(=O)(O)O)cc1)C(=O)N[C@@H](CC(C)C)C(=O)N1CCC[C@H]1C(=O)N[C@H](CCC(N)=O)C(=O)N[C@@H](C(N)=O)[C@H](C)O	600
**24**	CHEMBL433511	CC(=O)N[C@H](Cc1ccc(OP(=O)(O)O)cc1)C(=O)N[C@H](CC(C)C)C(=O)N1CCC[C@H]1C(=O)N[C@@H](CC(N)=O)C(=O)N[C@H](C(=O)N[C@H](C(N)=O)C(C)C)[C@@H](C)O	2000

**25**	CHEMBL157727	CC(=O)N[C@@H](Cc1ccc(OP(=O)(O)O)cc1)C(=O)N[C@@H](CC(C)C)C(=O)N1CCC[C@@H]1C(=O)N[C@H](CCC(N)=O)C(=O)N[C@@H](C(=O)N[C@H](C)C(=O)O)[C@@H](C)O	400
**26**	CHEMBL347438	CC(=O)N[C@@H](Cc1ccc(O)cc1)C(=O)N[C@@H](CC(C)C)C(=O)N1CCC[C@@H]1C(=O)N[C@@H](CCC(N)=O)C(=O)N[C@H](C(=O)N[C@H](C(N)=O)C(C)C)[C@@H](C)O	6000
**27**	CHEMBL351757	CSCC[C@H](NC(=O)[C@H](Cc1ccc(OP(=O)(O)O)cc1)NC(C)=O)C(=O)N1CCC[C@@H]1C(=O)N[C@@H](CCC(N)=O)C(N)=O	1500
**28[a]**	CHEMBL2371211	CC(=O)N[C@@H](Cc1ccc(OP(=O)(O)O)cc1)C(=O)N[C@@H](CC(C)C)C(=O)N[C@@H](CCCCN)C(=O)N[C@H](C(=O)N[C@@H](CCCCN)C(=O)N[C@@H](Cc1ccccc1)C(N)=O)[C@@H](C)O	20000
**29**	CHEMBL436427	CC(=O)N[C@@H](Cc1ccc(OP(=O)(O)O)cc1)C(=O)N[C@@H](CC(C)C)C(=O)N[C@@H](CCCCN)C(=O)N[C@@H](CCC(N)=O)C(=O)N[C@@H](CCC(=O)O)C(=O)N[C@@H](CO)C(N)=O	6000
**30**	CHEMBL160733	CSCC[C@H](NC(=O)[C@H](CCC(N)=O)NC(=O)[C@H]1CCCN1C(=O)[C@H](CCCCN)NC(=O)[C@H](Cc1ccc(OP(=O)(O)O)cc1)NC(C)=O)C(=O)N[C@@H](Cc1c[nH]cn1)C(N)=O	400
**31**	CHEMBL197719	CC(=O)N[C@@H](Cc1ccc(OP(=O)(O)O)cc1)C(=O)N[C@@H](CC(C)C)C(=O)N1CCN(CC(=O)N[C@@H](CCC(N)=O)C(=O)N[C@H](C(N)=O)[C@@H](C)O)CC1	57100
**32**	CHEMBL370000	CC(=O)N[C@@H](Cc1ccc(OP(=O)(O)O)cc1)C(=O)N[C@@H](CC(C)C)C(=O)N1CCC[C@H]1C(=O)N[C@@H](CCC(N)=O)C(=O)N1CCCCC1	7000
**33**	CHEMBL371452	CC(=O)N[C@@H](Cc1ccc(OP(=O)(O)O)cc1)C(=O)N[C@@H](CC(C)C)C(=O)N1CCC[C@H]1C(=O)N[C@@H](CC[S+](C)[O-])C(=O)N[C@H](C(N)=O)[C@@H](C)O	9870
**34[a]**	CHEMBL196561	CC(=O)N[C@@H](Cc1ccc(OP(=O)(O)O)cc1)C(=O)N[C@@H](CC(C)C)C(=O)N1CCC[C@H]1C(=O)NCCCC(N)=O	1780
**35**	CHEMBL382978	CC(=O)N[C@@H](Cc1ccc(OP(=O)(O)O)cc1)C(=O)N[C@@H](CC(C)C)C(=O)N1CCC[C@H]1C(=O)N[C@@H](CCC(N)=O)C(=O)N[C@H](C(N)=O)[C@@H](C)O	600
**36**	CHEMBL194446	CC(=O)N[C@@H](Cc1ccc(OP(=O)(O)O)cc1)C(=O)N[C@@H](CC(C)C)C(=O)N1CCC[C@H]1C(=O)NC1CCCC(C(N)=O)C1	9810
**37**	CHEMBL436319	CC(=O)N[C@@H](Cc1ccc(OP(=O)(O)O)cc1)C(=O)N[C@@H](CC(C)C)C(=O)N1C[C@H]2C[C@H]2[C@H]1C(=O)N[C@@H](CCC(N)=O)C(=O)N[C@H](C(N)=O)[C@@H](C)O	3110

**38**	CHEMBL197513	CCCC[C@H](NC(=O)[C@H](Cc1ccc(OP(=O)(O)O)cc1)NC(C)=O)C(=O)N1CCC[C@H]1C(=O)N[C@@H](CCC(N)=O)C(=O)N[C@H](C(N)=O)[C@@H](C)O	717
**39**	CHEMBL197580	CCC(=O)N[C@@H](Cc1ccc(OP(=O)(O)O)cc1)C(=O)N[C@@H](CC(C)C)C(=O)N1CCC[C@H]1C(=O)N[C@@H](CCC(N)=O)C(=O)N[C@H](C(N)=O)[C@@H](C)O	497
**40**	CHEMBL366244	CC(=O)N[C@@H](Cc1ccc(OP(=O)(O)O)cc1)C(=O)N[C@@H](CC(C)C)C(=O)N1CCC[C@H]1C(=O)N1CCCC(C(N)=O)C1	11800
**41**	CHEMBL370095	CC(=O)N[C@@H](Cc1ccc(OP(=O)(O)O)cc1)C(=O)N[C@@H](CC(C)C)C(=O)N1CCC[C@H]1C(=O)N1CCCC(C(N)=O)C1	882
**42**	CHEMBL382709	CC(=O)N[C@@H](Cc1ccc(OP(=O)(O)O)cc1)C(=O)N[C@@H](CC(C)C)C(=O)N1CCC[C@H]1C(=O)N[C@@H](CCC(N)=O)C(=O)N(C)C	1850
**43**	CHEMBL372758	CC(=O)N[C@@H](Cc1ccc(OP(=O)(O)O)cc1)C(=O)N[C@@H](CC(C)C)C(=O)N1CC[C@H]1C(=O)N[C@@H](CCC(N)=O)C(=O)N[C@H](C(N)=O)[C@@H](C)O	1660
**44**[a]	CHEMBL370547	CC(=O)N[C@@H](Cc1ccc(OP(=O)(O)O)cc1)C(=O)N[C@@H](CC(C)C)C(=O)N1CC[C@H]1C(=O)N[C@@H](CCC(N)=O)C(=O)N[C@H](C(N)=O)[C@@H](C)O	13400
**45**	CHEMBL194453	CC(=O)N[C@@H](Cc1ccc(OP(=O)(O)O)cc1)C(=O)N[C@@H](CC(C)C)C(=O)N1CCC[C@H]1C(=O)N[C@@H](CCC(=O)N(C)C)C(=O)N[C@H](C(N)=O)[C@@H](C)O	80000
**46**	CHEMBL193433	CC(=O)N[C@@H](Cc1ccc(OP(=O)(O)O)cc1)C(=O)N[C@@H](CC(C)C)C(=O)N1CCC[C@H]1C(=O)N[C@@H](CCC(N)=O)C(=O)N[C@H](C)c1ccccc1	801
**47**[a]	CHEMBL425747	CC(=O)N[C@@H](Cc1ccc(OP(=O)(O)O)cc1)C(=O)N[C@@H](CC(C)C)C(=O)N1CCCC(C(=O)N[C@@H](CCC(N)=O)C(=O)N[C@H](C(N)=O)[C@@H](C)O)C1	38600
**48**	CHEMBL371537	CC[C@H](C)[C@H](NC(=O)[C@H](Cc1ccc(OP(=O)(O)O)cc1)NC(C)=O)C(=O)N1CCC[C@H]1C(=O)N[C@@H](CCC(N)=O)C(=O)N[C@H](C(N)=O)[C@@H](C)O	1070
**49**[a]	CHEMBL371871	CC(=O)N[C@@H](Cc1ccc(OP(=O)(O)O)cc1)C(=O)N[C@@H](CC1CCCCC1)C(=O)N1CCC[C@H]1C(=O)N[C@@H](CCC(N)=O)C(=O)N[C@H](C(N)=O)[C@@H](C)O	910
**50**	CHEMBL372118	CC(=O)N[C@@H](Cc1ccc(OP(=O)(O)O)cc1)C(=O)N[C@@H](CC(C)C)C(=O)N1CCC[C@@]1(C)C(=O)N[C@@H](CCC(N)=O)C(=O)N[C@H](C(N)=O)[C@@H](C)O	1870

**51**	CHEMBL371705	CC(=O)N[C@@H](Cc1ccc(OP(=O)(O)O)cc1)C(=O)N[C@@H](CC(C)C)C(=O)N1CCCC[C@H]1C(=O)N[C@@H](CCC(N)=O)C(=O)N[C@H](C(N)=O)[C@@H](C)O	1240
**52**	CHEMBL196884	CC(=O)N[C@@H](Cc1ccc(OP(=O)(O)O)cc1)C(=O)N[C@@H](CC(C)C)C(=O)N1CCC[C@H]1C(=O)N1CCN(CC(N)=O)CC1	1400
**53**	CHEMBL382544	CC(=O)N[C@@H](Cc1ccc(OP(=O)(O)O)cc1)C(=O)N[C@@H](CC(C)C)C(=O)N1CCC[C@H]1C(=O)N[C@@H](CCC(N)=O)C(=O)O	748
**54**[a]	CHEMBL436479	CC(=O)N[C@@H](Cc1ccc(OP(=O)(O)O)cc1)C(=O)N[C@@H](CC(C)C)C(=O)N1CCC[C@H]1C(=O)N1CCC(CC(N)=O)C1	1690
**55**	CHEMBL197658	CC(=O)N[C@@H](Cc1ccc(OP(=O)(O)O)cc1)C(=O)N[C@H](C(=O)N1CCC[C@H]1C(=O)N[C@@H](CCC(N)=O)C(=O)N[C@H](C(N)=O)[C@@H](C)O)C(c1ccccc1)c1ccccc1	>100000
**56**	CHEMBL372993	CC(=O)N[C@@H](Cc1ccc(OP(=O)(O)O)cc1)C(=O)N[C@@H](CC(C)C)C(=O)N1CCC[C@H]1C(=O)N[C@@H](CCC(N)=O)C(=O)N1CCCC1	2110
**57**	CHEMBL372429	CC(=O)N[C@@H](Cc1ccc(OP(=O)(O)O)cc1)C(=O)N[C@@H](CC(C)C)C(=O)N1C[C@H](O)C[C@H]1C(=O)N[C@@H](CCC(N)=O)C(=O)N[C@H](C(N)=O)[C@@H](C)O	484
**58**	CHEMBL371056	CC(=O)N[C@@H](Cc1ccc(OP(=O)(O)O)cc1)C(=O)N[C@@H](CC(C)C)C(=O)N1CCC(C(=O)N[C@@H](CCC(N)=O)C(=O)N[C@H](C(N)=O)[C@@H](C)O)CC1	34600
**59**	CHEMBL365749	CC(=O)N[C@@H](Cc1ccc(OP(=O)(O)O)cc1)C(=O)N[C@@H](CC(C)C)C(=O)N1CCC[C@H]1C(=O)NCCCCCC(N)=O	9780
**60**	CHEMBL196664	CC(=O)N[C@@H](Cc1ccc(OP(=O)(O)O)cc1)C(=O)N[C@@H](CC(C)C)C(=O)N1CCC[C@H]1C(=O)N[C@@H](CCC(N)=O)C(=O)N(C)[C@H](C(N)=O)[C@@H](C)O	1220
**61**	CHEMBL370763	CC(C)C[C@H](NC(=O)[C@H](Cc1ccc(OP(=O)(O)O)cc1)NC(=O)CCc1ccccc1)C(=O)N1C[C@H]2C[C@H]2[C@H]1C(=O)N[C@@H](CCC(N)=O)C(=O)NCc1ccccc1	125
**62**[a]	CHEMBL370649	CC(=O)N[C@@H](Cc1ccc(OP(=O)(O)O)cc1)C(=O)N[C@@H](CC(C)C)C(=O)N1CCC[C@@H]1C(=O)N[C@@H](CCC(N)=O)C(=O)N[C@H](C(N)=O)[C@@H](C)O	>100000
**63**	CHEMBL196122	CC(=O)N[C@@H](Cc1ccc(OP(=O)(O)O)cc1)C(=O)N[C@@H](CC(C)C)C(=O)N1CCC[C@H]1C(=O)N[C@@H](CCS(C)(=O)=O)C(=O)N[C@H](C(N)=O)[C@@H](C)O	13900

**64**	CHEMBL425571	CC(C)C[C@H](NC(=O)[C@H](Cc1ccc(OP(=O)(O)O)cc1)NC(=O)c1ccccc1)C(=O)N1CCC[C@H]1C(=O)N[C@@H](CCC(N)=O)C(=O)N[C@H](C(N)=O)[C@@H](C)O	814
**65**	CHEMBL435121	CC(C)C[C@H](NC(=O)[C@H](Cc1ccc(OP(=O)(O)O)cc1)NC(=O)OCc1ccccc1)C(=O)N1CCC[C@H]1C(=O)N[C@@H](CCC(N)=O)C(=O)N[C@H](C(N)=O)[C@@H](C)O	750
**66**[a]	CHEMBL373124	CC(=O)N[C@@H](Cc1ccc(OP(=O)(O)O)cc1)C(=O)N[C@@H](CC(C)C)C(=O)N1CCC[C@H]1C(=O)N1CCC(C(N)=O)CC1	3020
**67**	CHEMBL370208	CC(=O)N[C@@H](Cc1ccc(OP(=O)(O)O)cc1)C(=O)N[C@@H](CC(C)C)C(=O)N1CCC[C@H]1C(=O)N[C@@H](CCC(N)=O)C(=O)N[C@@H](C)c1ccccc1	596
**68**	CHEMBL194855	CC(=O)N[C@@H](Cc1ccc(OP(=O)(O)O)cc1)C(=O)N[C@@H](CC(C)C)C(=O)N1CCC[C@H]1C(=O)N[C@@H](CCC(N)=O)C(=O)NCc1ccccc1	409
**69**	CHEMBL198548	CC(=O)N[C@@H](Cc1ccc(OP(=O)(O)O)cc1)C(=O)N[C@H](C(=O)N1CCC[C@H]1C(=O)N[C@@H](CCC(N)=O)C(=O)N[C@H](C(N)=O)[C@@H](C)O)C(C)C	1540
**70**	CHEMBL442785	CC(=O)N[C@@H](Cc1ccc(OP(=O)(O)O)cc1)C(=O)N[C@@H](Cc1ccccc1)C(=O)N1CCC[C@H]1C(=O)N[C@@H](CCC(N)=O)C(=O)N[C@H](C(N)=O)[C@@H](C)O	1890
**71**	CHEMBL194427	CC(=O)N[C@@H](Cc1ccc(OP(=O)(O)O)cc1)C(=O)N(C)[C@@H](CC(C)C)C(=O)N1CCC[C@H]1C(=O)N[C@@H](CCC(N)=O)C(=O)N[C@H](C(N)=O)[C@@H](C)O	>100000
**72**[a]	CHEMBL197844	CC(C)C[C@H](NC(=O)[C@H](Cc1ccc(OP(=O)(O)O)cc1)NC(=O)CCc1ccccc1)C(=O)N1CCC[C@H]1C(=O)N[C@@H](CCC(N)=O)C(=O)N[C@H](C(N)=O)[C@@H](C)O	276
**73**	CHEMBL373136	CC(=O)N[C@@H](Cc1ccc(OP(=O)(O)O)cc1)C(=O)N[C@@H](CC(C)C)C(=O)N1CCC[C@H]1C(=O)Nc1cccc(C(N)=O)c1	36600
**74**	CHEMBL436504	CNC(=O)CC[C@H](NC(=O)[C@@H]1CCCN1C(=O)[C@H](CC(C)C)NC(=O)[C@H](Cc1ccc(OP(=O)(O)O)cc1)NC(C)=O)C(=O)N[C@H](C(N)=O)[C@@H](C)O	18100
**75**	CHEMBL193800	CCN(CC)C(=O)[C@H](CCC(N)=O)NC(=O)[C@@H]1CCCN1C(=O)[C@H](CC(C)C)NC(=O)[C@H](Cc1ccc(OP(=O)(O)O)cc1)NC(C)=O	4220
**76**[a]	CHEMBL508923	O=C(Cc1ccc2c(c1)OCO2)NCCc1ccc(O)c(O)c1	357000
**77**	CHEMBL524275	Cc1oc(C)c2c(=O)cc(-c3ccc(O)c(O)c3)cc(O)c12	106000

**78**[a]	CHEMBL414034	CC(C)C[C@H](NC(=O)/C=C/c1ccc(OP(=O)(O)O)cc1)C(=O)N1CCC[C@H]1C(=O)N[C@@H](COC(N)=O)C(=O)NCc1ccccc1	379
**79**	CHEMBL267246	CC(C)C[C@H](NC(=O)/C=C/c1ccc(OP(=O)(O)O)cc1)C(=O)N1CCC[C@H]1C(=O)N[C@@H](CCC(N)=O)C(=O)NCc1ccccc1	135
**80**	CHEMBL376795	CC(C)C[C@H](NC(=O)/C=C/c1ccc(OP(=O)(O)O)cc1)C(=O)N1CCC[C@H]1C(=O)N[C@H](C(=O)NCc1ccccc1)[C@@H](C)OC(N)=O	850
**81**	CHEMBL398067	CC(C)C[C@H](NC(=O)[C@H](Cc1ccc(OP(=O)(O)O)cc1)NC(=O)c1ccc(C#N)cc1)C(=O)NCc1ccccc1	800000
**82**	CHEMBL412549	COc1cccc(NC(=O)[C@H](CC(C)C)NC(=O)[C@H](Cc2ccc(OP(=O)(O)O)cc2)NC(=O)c2ccc(C#N)cc2)c1	31000
**83**	CHEMBL393817	CC(C)C[C@H](NC(=O)[C@H](Cc1ccc(OP(=O)(O)O)cc1)NC(=O)c1ccc(C#N)cc1)C(=O)NC1CCCCC1	58000
**84**	CHEMBL234465	CC(C)CNC(=O)[C@H](CC(C)C)NC(=O)[C@H](Cc1ccc(OP(=O)(O)O)cc1)NC(=O)c1ccc(C#N)cc1	74000
**85**[a]	CHEMBL234817	CC(C)C[C@H](NC(=O)[C@H](Cc1ccc(OP(=O)(O)O)cc1)NC(=O)c1ccc(C#N)cc1)C(=O)NCc1ccncc1	123000
**86**	CHEMBL234466	CC(C)C[C@H](NC(=O)[C@H](Cc1ccc(OP(=O)(O)O)cc1)NC(=O)c1ccc(C#N)cc1)C(=O)NCCCO	620000
**87**	CHEMBL392556	CC(C)C[C@H](NC(=O)[C@H](Cc1ccc(OP(=O)(O)O)cc1)NC(=O)c1ccc(C#N)cc1)C(=O)O	310000
**88**	CHEMBL438852	CC(C)C[C@H](NC(=O)[C@H](Cc1ccc(OP(=O)(O)O)cc1)NC(=O)c1ccc(C#N)cc1)C(=O)Nc1ccccc1	310000
**89**	CHEMBL439390	CC(=O)N[C@@H](Cc1ccc(-c2nn[nH]n2)cc1)C(=O)N[C@@H](CC(C)C)C(=O)N1CCC[C@H]1C(=O)N[C@@H](CCC(N)=O)C(=O)N[C@H](C(=O)N[C@H](C(=O)O)C(C)C)[C@@H](C)O	800000
**90**	CHEMBL414897	CC(=O)N[C@@H](Cc1ccc(OCC(=O)O)cc1)C(=O)N[C@@H](CC(C)C)C(=O)N1CCC[C@H]1C(=O)N[C@@H](CCC(N)=O)C(=O)N[C@H](C(=O)N[C@H](C(=O)O)C(C)C)[C@@H](C)O	1000
**91**	CHEMBL231705	CC(=O)N[C@@H](Cc1ccc(OCC(=O)O)cc1)C(=O)N[C@@H](CC(C)C)C(=O)N1CCC[C@H]1C(=O)N[C@@H](CCC(N)=O)C(=O)N[C@H](C(=O)N[C@H](C(=O)O)C(C)C)[C@@H](C)O	108000
**92**	CHEMBL395165	CC(=O)N[C@@H](Cc1ccc(OP(=O)(O)O)cc1)C(=O)N[C@@H](CC(C)C)C(=O)N1CCC[C@H]1C(=O)N[C@@H](CCC(N)=O)C(=O)N[C@H](C(=O)N[C@H](C(=O)O)C(C)C)[C@@H](C)O	9000

**93**[a]	CHEMBL396939	CC(=O)N[C@@H](Cc1ccc(CP(=O)(O)O)cc1)C(=O)N[C@@H](CC(C)C)C(=O)N1CCC[C@H]1C(=O)N[C@@H](CCC(N)=O)C(=O)N[C@H](C(=O)N[C@H](C(=O)O)C(C)C)[C@@H](C)O	138000
**94**	CHEMBL233355	CC(=O)N[C@@H](Cc1ccc(Cc2nn[nH]n2)cc1)C(=O)N[C@@H](CC(C)C)C(=O)N1CCC[C@H]1C(=O)N[C@@H](CCC(N)=O)C(=O)N[C@H](C(=O)N[C@H](C(=O)O)C(C)C)[C@@H](C)O	1000
**95**	CHEMBL443407	CC(C)C[C@H](NC(=O)c1ccc2cc(OP(=O)(O)O)ccc2c1)C(=O)N1CCC[C@H]1C(=O)N[C@@H](CCC(N)=O)C(=O)N[C@H](C(N)=O)[C@@H](C)O	>100000
**96**	CHEMBL471109	CC(C)C[C@H](NC(=O)Cc1c[nH]c2ccc(OP(=O)(O)O)cc12)C(=O)N1CCC[C@H]1C(=O)N[C@@H](CCC(N)=O)C(=O)N[C@H](C(N)=O)[C@@H](C)O	>100000
**97**	CHEMBL505076	CC(=O)N[C@@H](Cc1ccc(OP(=O)(O)O)cc1)C(=O)N[C@@H](CC(C)C)C(=O)N1CCC[C@H]1C(=O)N[C@@H](CCC(N)=O)C(=O)N[C@H](C(N)=O)[C@H](C)O	739
**98**	CHEMBL454365	CNC(=O)[C@H](CCC(N)=O)NC(=O)[C@@H]1Cc2cccc3c2N1C(=O)[C@@H](NC(=O)/C=C/c1ccc(OP(=O)(O)O)cc1)CC3	269
**99**	CHEMBL2372260	CC(=O)N1Cc2ccc(OP(=O)(O)O)cc2C[C@H]1C(=O)N[C@@H](CC(C)C)C(=O)N1CCC[C@H]1C(=O)N[C@@H](CCC(N)=O)C(=O)N[C@H](C(N)=O)[C@@H](C)O	>100000
**100**	CHEMBL503765	CC(=O)N[C@@H](Cc1ccc(OP(=O)(O)O)cc1)C(=O)N[C@H]1CCc2cccc3c2N(C1=O)[C@H](C(=O)N[C@@H](CCC(N)=O)C(=O)N[C@H](C(N)=O)[C@@H](C)O)C3	231
**101**	CHEMBL505207	NC(=O)CC[C@H](NC(=O)[C@@H]1CC[C@H]2CC[C@H](NC(=O)/C=C/c3ccc(OP(=O)(O)O)cc3)C(=O)N12)C(=O)NCc1ccccc1	45700
**102**	CHEMBL449547	NC(=O)CC[C@H](NC(=O)[C@@H]1Cc2cccc3c2N1C(=O)[C@@H](NC(=O)/C=C/c1ccc(OP(=O)(O)O)cc1)CC3)C(=O)NCc1ccccc1	162
**103**	CHEMBL515762	NC(=O)C1CCN(C(=O)[C@@H]2Cc3cccc4c3N2C(=O)[C@@H](NC(=O)/C=C/c2ccc(OP(=O)(O)O)cc2)CC4)C1	595
**104**	CHEMBL503946	C[S+]([O-])CC[C@H](NC(=O)[C@@H]1Cc2cccc3c2N1C(=O)[C@@H](NC(=O)/C=C/c1ccc(OP(=O)(O)O)cc1)CC3)C(N)=O	7600
**105**	CHEMBL500738	CC(C)C[C@H](NC(=O)/C=C/c1cccc(OP(=O)(O)O)c1)C(=O)N1CCC[C@H]1C(=O)N[C@@H](CCC(N)=O)C(=O)N[C@H](C(N)=O)[C@@H](C)O	>100000
**106**	CHEMBL504218	NC(=O)CC[C@H](NC(=O)[C@H]1CC[C@H]2CC[C@H](NC(=O)/C=C/c3ccc(OP(=O)(O)O)cc3)C(=O)N21)C(=O)NCc1ccccc1	59800

**107[a]**	CHEMBL508912	NC(=O)CC[C@H](NC(=O)[C@@H]1Cc2cccc3c2N1C(=O)[C@@H](NC(=O)/C=C/c1ccc(OP(=O)(O)O)cc1)C=C3)C(=O)NCc1ccccc1	195
**108**	CHEMBL501981	CC(=O)N[C@@H](Cc1ccc(OP(=O)(O)O)cc1)C(=O)N[C@@H]1CCc2cccc3c2N(C1=O)[C@H](C(=O)N[C@@H](CCC(N)=O)C(=O)N[C@H](C(N)=O)[C@@H](C)O)C3	1650
**109[a]**	CHEMBL486267	C[C@H](NC(=O)[C@@H]1Cc2cccc3c2N1C(=O)[C@@H](NC(=O)/C=C/c1ccc(OP(=O)(O)O)cc1)CC3)C(N)=O	25060
**110**	CHEMBL485921	CC(C)C[C@H](NC(=O)c1cc2cc(OP(=O)(O)O)ccc2[nH]1)C(=O)N1CCC[C@H]1C(=O)N[C@@H](CCC(N)=O)C(=O)N[C@H](C(N)=O)[C@@H](C)O	186
**111**	CHEMBL504633	CC(=O)N[C@@H](Cc1ccc(OP(=O)(O)O)cc1)C(=O)N[C@@H](CC(C)C)C(=O)N1c2ccccc2C[C@H]1C(=O)N[C@@H](CCC(N)=O)C(=O)N[C@H](C(N)=O)[C@@H](C)O	4640
**112**	CHEMBL475488	O=C(O)CCCNC(=O)[C@@H]1Cc2cccc3c2N1C(=O)[C@@H](NC(=O)/C=C/c1ccc(OP(=O)(O)O)cc1)CC3	815
**113[a]**	CHEMBL503365	COC(=O)[C@H](CCC(N)=O)NC(=O)[C@@H]1Cc2cccc3c2N1C(=O)[C@@H](NC(=O)/C=C/c1ccc(OP(=O)(O)O)cc1)CC3	141
**114**	CHEMBL447460	CC(C)NC(=O)[C@H](CCC(N)=O)NC(=O)[C@@H]1Cc2cccc3c2N1C(=O)[C@@H](NC(=O)/C=C/c1ccc(OP(=O)(O)O)cc1)CC3	815
**115[a]**	CHEMBL470227	CC(C)C[C@H](NC(=O)/C=C/c1ccc(OP(=O)(O)O)cc1)C(=O)N1CCC[C@H]1C(=O)N[C@@H](CCC(N)=O)C(=O)N[C@H](C(N)=O)[C@@H](C)O	136
**116[a]**	CHEMBL448296	CC(=O)N[C@@H](Cc1ccc(OP(=O)(O)O)cc1)C(=O)N[C@H]1CC[C@H]2CC[C@@H](C(=O)N[C@@H](CCC(N)=O)C(=O)N[C@H](C(N)=O)[C@@H](C)O)N2C1=O	141
**117**	CHEMBL502869	CC(=O)N[C@@H](Cc1ccc(OP(=O)(O)O)cc1)C(=O)N[C@H]1CC[C@@H]2CC[C@H](C(=O)N[C@@H](CCC(N)=O)C(=O)N[C@H](C(N)=O)[C@@H](C)O)N2C1=O	24700
**118**	CHEMBL510008	NC(=O)CC[C@H](NC(=O)[C@@H]1Cc2cccc3c2N1C(=O)[C@@H](NC(=O)/C=C/c1ccc(OP(=O)(O)O)cc1)CC3)C(=O)O	359
**119**	CHEMBL455671	CS(=O)(=O)CC[C@H](NC(=O)[C@@H]1Cc2cccc3c2N1C(=O)[C@@H](NC(=O)/C=C/c1ccc(OP(=O)(O)O)cc1)CC3)C(N)=O	7030
**120**	CHEMBL449511	NC(=O)CC[C@H](NC(=O)[C@@H]1Cc2cccc3c2N1C(=O)[C@@H](NC(=O)/C=C/c1ccc(OP(=O)(O)O)cc1)CC3)C(N)=O	233
**121**	CHEMBL513887	NC(=O)[C@@H]1Cc2cccc3c2N1C(=O)[C@@H](NC(=O)/C=C/c1ccc(OP(=O)(O)O)cc1)CC3	35600

**122**	CHEMBL508900	C[C@@H](O)[C@H](NC(=O)[C@H](CCC(N)=O)NC(=O)[C@@H]1Cc2cccc3c2N1C(=O)[C@@H](NC(=O)CCc1ccc(OP(=O)(O)O)cc1)CC3)C(N)=O	3240
**123**	CHEMBL445970	NC(=O)CC[C@H](NC(=O)[C@@H]1CC[C@@H]2CC[C@H](NC(=O)/C=C/c3ccc(OP(=O)(O)O)cc3)C(=O)N21)C(=O)NCc1ccccc1	604
**124**	CHEMBL452194	NC(=O)CC[C@H](NC(=O)[C@@H]1Cc2cccc3c2N1C(=O)[C@@H](NC(=O)c1cc2cc(OP(=O)(O)O)ccc2[nH]1)CC3)C(=O)NCc1ccccc1	190
**125[a]**	CHEMBL468082	CCCC[C@H](NC(=O)[C@H](C)NC(=O)[C@@H]1Cc2cccc3c2N1C(=O)[C@@H](NC(=O)/C=C/c1ccc(OP(=O)(O)O)cc1)CC3)C(N)=O	19200
**126[a]**	CHEMBL573901	CC(C)C[C@H](NC(=O)/C=C/c1ccc(OP(=O)(O)O)cc1)C(=O)N1CCC[C@H]1C(=O)N[C@@H](CC(N)=O)C(=O)NCc1ccccc1	874
**127[a]**	CHEMBL573930	CC(C)C[C@H](NC(=O)/C=C/c1ccc(OP(=O)(O)O)cc1)C(=O)N1C[C@H]2C[C@H]2[C@H]1C(=O)N[C@@H](CCC(N)=O)C(C)C	511
**128**	CHEMBL583726	CC(=O)OC[C@H](CCC(N)=O)NC(=O)[C@@H]1[C@@H]2C[C@@H]2CN1C(=O)[C@H](CC(C)C)NC(=O)/C=C/c1ccc(OP(=O)(O)O)cc1	290
**129**	CHEMBL573048	CC(C)C[C@H](NC(=O)/C=C/c1ccc(OP(=O)(O)O)cc1)C(=O)N1CCC[C@H]1C(=O)NCCCC(N)=O	574
**130**	CHEMBL575513	CC(C)C[C@H](NC(=O)/C=C/c1ccc(OP(=O)(O)O)cc1)C(=O)N1CCC[C@H]1C(=O)N[C@@H](CCC(N)=O)CN(Cc1ccccc1)Cc1ccccc1	1080
**131**	CHEMBL574844	CC(C)C[C@H](CCC(N)=O)NC(=O)[C@@H]1CCCN1C(=O)[C@H](CC(C)C)NC(=O)/C=C/c1ccc(OP(=O)(O)O)cc1	4900
**132**	CHEMBL573903	CC(C)C[C@H](NC(=O)/C=C/c1ccc(OP(=O)(O)O)cc1)C(=O)N1CCC[C@H]1C(=O)N[C@@H](CCC(N)=O)Cc1ccc(O)cc1	848
**133[a]**	CHEMBL575919	CC(C)C[C@H](NC(=O)/C=C/c1ccc(OP(=O)(O)O)cc1)C(=O)N1CCC[C@H]1C(=O)NCC#CC(N)=O	1140
**134**	CHEMBL574636	CC(C)C[C@H](NC(=O)/C=C/c1ccc(OP(=O)(O)O)cc1)C(=O)N1CCC[C@H]1C(=O)NCCNC(N)=O	131
**135[a]**	CHEMBL577143	CC(C)C[C@H](NC(=O)/C=C/c1ccc(OP(=O)(O)O)cc1)C(=O)N1C[C@H]2C[C@H]2[C@H]1C(=O)NCCCC(N)=O	303
**136**	CHEMBL573902	CC(C)C[C@H](NC(=O)/C=C/c1ccc(OP(=O)(O)O)cc1)C(=O)N1CCC[C@H]1C(=O)N[C@H](COCc1ccccc1)COC(N)=O	454
**137**	CHEMBL583116	CCCC[C@H](CCC(N)=O)NC(=O)[C@@H]1[C@@H]2C[C@@H]2CN1C(=O)[C@H](CC(C)C)NC(=O)/C=C/c1ccc(OP(=O)(O)O)cc1	499
**138**	CHEMBL573049	CC(C)C[C@H](NC(=O)/C=C/c1ccc(OP(=O)(O)O)cc1)C(=O)N1CCC[C@H]1C(=O)N[C@@H](C)CCC(N)=O	1180

**139**	CHEMBL583756	CC(C)C[C@H](NC(=O)/C=C/c1ccc(OP(=O)(O)O)cc1)C(=O)N1CCC[C@H]1C(=O)N[C@@H](COC(N)=O)[C@@H](C)OCc1ccccc1	6900
**140**	CHEMBL576590	CC(C)C[C@H](NC(=O)/C=C/c1ccc(OP(=O)(O)O)cc1)C(=O)N1CCC[C@H]1C(=O)NCCOC(N)=O	360
**141**	CHEMBL573905	CC(C)C[C@H](NC(=O)/C=C/c1ccc(OP(=O)(O)O)cc1)C(=O)N1CCC[C@H]1C(=O)N[C@@H](CCC(N)=O)CN1CCN(C)CC1	1310
**142**	CHEMBL577593	CC(C)C[C@H](NC(=O)/C=C/c1ccc(OP(=O)(O)O)cc1)C(=O)N1CCC[C@H]1C(=O)N[C@@H]1CN[C@H](C(N)=O)C1	3210
**143**	CHEMBL576785	CCCC[C@H](CCC(N)=O)NC(=O)[C@@H]1CCCN1C(=O)[C@H](CC(C)C)NC(=O)/C=C/c1ccc(OP(=O)(O)O)cc1	1350
**144**	CHEMBL583727	CC(C)C[C@H](NC(=O)/C=C/c1ccc(OP(=O)(O)O)cc1)C(=O)N1C[C@H]2C[C@H]2[C@H]1C(=O)N[C@@H](CCC(N)=O)COCc1ccccc1	94
**145**	CHEMBL575098	CC(C)C[C@H](NC(=O)/C=C/c1ccc(OP(=O)(O)O)cc1)C(=O)N1CCC[C@H]1C(=O)NCCCC(=O)NN	1830
**146[a]**	CHEMBL583758	CC(C)C[C@H](NC(=O)/C=C/c1ccc(OP(=O)(O)O)cc1)C(=O)N1CCC[C@H]1C(=O)N[C@@H](CCC(N)=O)[C@@H](C)OCc1ccccc1	272
**147**	CHEMBL576784	CC(C)C[C@H](NC(=O)/C=C/c1ccc(OP(=O)(O)O)cc1)C(=O)N1C[C@H]2C[C@H]2[C@H]1C(=O)N[C@H](C)CCC(N)=O	175
**148[a]**	CHEMBL573068	CC(C)C[C@H](NC(=O)/C=C/c1ccc(OP(=O)(O)O)cc1)C(=O)N1CCC[C@H]1C(=O)N[C@H]1CN[C@H](C(N)=O)C1	3600
**149**	CHEMBL584376	CC(C)C[C@H](NC(=O)/C=C/c1ccc(OP(=O)(O)O)cc1)C(=O)N1CCC[C@H]1C(=O)N[C@H](C)COC(N)=O	797
**150**	CHEMBL575497	CC(C)C[C@H](NC(=O)/C=C/c1ccc(OP(=O)(O)O)cc1)C(=O)N1C[C@H]2C[C@H]2[C@H]1C(=O)N[C@@H](CCC(N)=O)C(=O)NCc1ccccc1	68
**151**	CHEMBL583754	CC(C)C[C@H](NC(=O)/C=C/c1ccc(OP(=O)(O)O)cc1)C(=O)N1CCC[C@H]1C(=O)N[C@@H](CCCC(N)=O)C(=O)NCc1ccccc1	1400
**152**	CHEMBL578430	CC(C)C[C@H](NC(=O)/C=C/c1ccc(OP(=O)(O)O)cc1)C(=O)N1CCC[C@H]1C(=O)N1CCC[C@H]1CCC(N)=O	3310
**153**	CHEMBL502473	CC(=O)N[C@@H](Cc1ccc(OP(=O)(O)O)cc1)C(=O)N[C@@H](CC(C)C)C(=O)N1CCC[C@H]1C(=O)N[C@@H](CCC(N)=O)C(=O)N[C@H](C(=O)N[C@H](C(N)=O)C(C)C)[C@H](C)O	290
**154**	CHEMBL574202	CC(C)C[C@H](NC(=O)/C=C/c1ccc(OP(=O)(O)O)cc1)C(=O)N1CCC[C@H]1C(=O)NC/C=C\C(N)=O	1110
**155**	CHEMBL583725	CC(C)C[C@H](NC(=O)/C=C/c1ccc(OP(=O)(O)O)cc1)C(=O)N1C[C@H]2C[C@H]2[C@H]1C(=O)N[C@@H](CCC(N)=O)[C@@H](C)O	437

**156**[a]	CHEMBL583138	CC(C)C[C@H](NC(=O)/C=C/c1ccc(OP(=O)(O)O)cc1)C(=O)N1CCC[C@H]1C(=O)NCC(=O)NCc1ccccc1	4940
**157**	CHEMBL574654	CC(C)C[C@H](NC(=O)/C=C/c1ccc(OP(=O)(O)O)cc1)C(=O)N1CCC[C@H]1C(N)=O	11400
**158**	CHEMBL578233	CC(C)C[C@H](NC(=O)/C=C/c1ccc(OP(=O)(O)O)cc1)C(=O)N1CCC[C@H]1C(=O)N[C@@H](C)C(N)=O	7800
**159**	CHEMBL575920	CC(C)C[C@H](NC(=O)/C=C/c1ccc(OP(=O)(O)O)cc1)C(=O)N1CCC[C@H]1C(=O)N[C@@H](CCC(N)=O)C(C)C	1880
**160**	CHEMBL573904	CC(C)C[C@H](NC(=O)/C=C/c1ccc(OP(=O)(O)O)cc1)C(=O)N1CCC[C@H]1C(=O)N[C@@H](CCC(N)=O)COCc1ccccc1	294
**161**	CHEMBL583963	CC(C)C[C@H](NC(=O)/C=C/c1ccc(OP(=O)(O)O)cc1)C(=O)N1CCC[C@H]1C(=O)N[C@@H](CCC(N)=O)CN1CCCCC1	1210
**162**[a]	CHEMBL574875	CC(C)C[C@H](NC(=O)/C=C/c1ccc(OP(=O)(O)O)cc1)C(=O)N1CCC[C@H]1C(=O)NCCCC(=O)NO	2640
**163**	CHEMBL576736	CC(C)C[C@H](NC(=O)/C=C/c1ccc(OP(=O)(O)O)cc1)C(=O)N1CCC[C@H]1C(=O)NCCCS(N)(=O)=O	68200
**164**	CHEMBL573069	CC(C)C[C@H](NC(=O)/C=C/c1ccc(OP(=O)(O)O)cc1)C(=O)N1CCC[C@H]1C(=O)N[C@H](C)CNC(N)=O	1350
**165**	CHEMBL573643	CC(C)C[C@H](NC(=O)/C=C/c1ccc(OP(=O)(O)O)cc1)C(=O)N1CCC[C@H]1C(=O)N[C@@H](CCC(N)=O)COC(N)=O	615
**166**	CHEMBL584974	CC(C)C[C@H](NC(=O)/C=C/c1ccc(OP(=O)(O)O)cc1)C(=O)N1CCC[C@H]1C(=O)N[C@@H](CCC(N)=O)CN1CCOCC1	1530
**167**	CHEMBL573980	CC(C)C[C@H](NC(=O)/C=C/c1ccc(OP(=O)(O)O)cc1)C(=O)N1CCC[C@H]1C(=O)N[C@H](CN)CCC(N)=O	1240
**168**	CHEMBL575484	CC(C)C[C@H](CCC(N)=O)NC(=O)[C@@H]1[C@@H]2C[C@@H]2CN1C(=O)[C@H](CC(C)C)NC(=O)/C=C/c1ccc(OP(=O)(O)O)cc1	1630
**169**	CHEMBL573443	CC(C)C[C@H](NC(=O)/C=C/c1ccc(OP(=O)(O)O)cc1)C(=O)N1C[C@H]2C[C@H]2[C@H]1C(=O)N[C@@H](CCC(N)=O)[C@@H](C)OCc1ccccc1	69
**170**	CHEMBL573442	CC(=O)NC[C@H](CCC(N)=O)NC(=O)[C@@H]1CCCN1C(=O)[C@H](CC(C)C)NC(=O)/C=C/c1ccc(OP(=O)(O)O)cc1	589
**171**	CHEMBL578635	CC(C)C[C@H](NC(=O)/C=C/c1ccc(OP(=O)(O)O)cc1)C(=O)N1CCC[C@H]1C(=O)N[C@H](C)CCC(N)=O	110
**172**	CHEMBL1290746	Cc1ccc(C(=O)Nc2ccon2)cc1NC(=O)c1cnn(-c2ccccc2F)c1N	10000
**173**	CHEMBL1687978	O=C(Nc1nnc(-c2ccco2)o1)c1cc(-c2cccc3ccccc23)nc2ccccc12	55000
**174**	CHEMBL1687967	O=C(Nc1nnc(-c2ccc(Cl)cc2)o1)c1cc(-c2ccccc2)nc2ccccc12	61000

**175**	CHEMBL1687977	O=C(Nc1nnc(-c2ccco2)o1)c1cc(-c2ccco2)nc2ccccc12	66000
**176**	CHEMBL1687965	O=C(Nc1nnc(-c2ccoc2)o1)c1cc(-c2ccccc2)nc2ccccc12	72000
**177[a]**	CHEMBL1687969	O=C(Nc1nnc(-c2ccccc2)o1)c1cc(-c2ccccc2)nc2ccccc12	66000
**178**	CHEMBL1687966	O=C(Nc1nnc(Cc2ccccc2)o1)c1cc(-c2ccccc2)nc2ccccc12	75000
**179**	CHEMBL1562195	O=C(Nc1nnc(-c2cccs2)o1)c1cc(-c2ccccc2)nc2ccccc12	75000
**180**	CHEMBL1687979	O=C(Nc1nnc(-c2ccco2)o1)c1cc(-c2ccccc2)nc2ccccc12	74000
**181**	CHEMBL1687976	O=C(Nc1nnc(-c2ccco2)o1)c1cc(-c2cccs2)nc2ccccc12	63000
**182**	CHEMBL2172008	CCN1C(=O)/C(=C/c2ccc(-c3ccc(C(=O)O)cc3)o2)SC1=S	30000
**183[a]**	CHEMBL2031566	O=C(O)CSc1cc(NS(=O)(=O)c2ccc(C(=O)O)cc2)c2ccccc2c1O	73000
**184**	CHEMBL2171900	COC(=O)Cc1c(C)c2ccc(OCc3ccc(C(=O)OC)cc3)c(C)c2oc1=O	63000
**185**	CHEMBL2171898	O=C(O)c1ccc(/C=C/C(=O)c2ccc3c(c2)OCCO3)cc1	91000
**186**	CHEMBL2171899	CCOc1cc(C=C2C(=O)c3ccccc3C2=O)cc(Cl)c1OCc1cccc(C(=O)O)c1	144000
**187**	CHEMBL2172009	CCN1C(=O)C(=Cc2ccc(-c3cc(C(=O)O)ccc3Cl)o2)C(=O)N(CC)C1=S	114000
**188**	CHEMBL187460	C[C@H]1COC2=C1C(=O)C(=O)c1c2ccc2c1CCCC2(C)C	4600
**189**	CHEMBL207155	C1=Cc2nc1cc1ccc([nH]1)c(-c1ccccc1)c1nc(cc3ccc([nH]3)c2-c2ccccc2)C=C1	280
**190**	CHEMBL1337170	O=[N+]([O-])c1ccc2c(c1)S(=O)(=O)C=C2	5100
**192**	CHEMBL2347240	Oc1ccc(NCc2cc(Cl)ccc2OCc2ccc(Cl)cc2)cc1	26680
**193**	CHEMBL2367507	CC(C)C[C@H](NC(=O)[C@H](Cc1ccc(O)cc1)NC(=O)[C@H]1CCCN1C(=O)CN)C(=O)N1CCC[C@H]1C(=O)N[C@@H](CCC(N)=O)C(=O)N[C@H](C(=O)N[C@H](C(N)=O)C(C)C)[C@H](C)O	300
**194**	CHEMBL2431922	O=C(O)c1ccc(N(Cc2ccc(C3CCCCC3)cc2)C(=O)CN(Cc2ccc(F)cc2)S(=O)(=O)c2c(F)c(F)c(F)c(F)c2F)cc1O	17600
**195**	CHEMBL2431990	Cc1ccc(S(=O)(=O)N(CC(=O)N(Cc2ccc(C3CCCCC3)cc2)c2ccc(C(=O)O)c(O)c2)Cc2ccccc2C)cc1	8800
**196**	CHEMBL2431924	O=C(O)c1ccc(N(Cc2ccc(C3CCCCC3)cc2)C(=O)CN(Cc2cccc(Cl)c2)S(=O)(=O)c2c(F)c(F)c(F)c(F)c2F)cc1O	18200
**197**	CHEMBL2429889	Cc1ccc(S(=O)(=O)N(CC(=O)N(Cc2ccc(C3CCCCC3)cc2)c2ccc(C(=O)O)c(O)c2)Cc2cc(F)c(F)c(F)c2F)cc1	22200
**198**	CHEMBL2431991	Cc1ccc(S(=O)(=O)N(CC(=O)N(Cc2ccc(C3CCCCC3)cc2)c2ccc(C(=O)O)c(O)c2)Cc2cccc(C)c2)cc1	5200
**199**	CHEMBL2431959	O=C(O)c1ccc(N(Cc2ccc(C3CCCCC3)cc2)C(=O)CN(Cc2cccc(F)c2)S(=O)(=O)c2c(F)c(F)c(F)c(F)c2F)cc1O	17800
**200**	CHEMBL2431977	Cc1ccc(S(=O)(=O)N(CC(=O)N(Cc2ccc(C3CCCCC3)cc2)c2ccc(C(=O)O)c(O)c2)Cc2ccncc2)cc1	46600

**201**[a]	CHEMBL2431971	Cc1ccc(S(=O)(=O)N(CC(=O)N(Cc2ccc(C3CCCCC3)cc2)c2ccc(C(=O)O)c(O)c2)Cc2ccc([N+](=O)[O-])cc2)cc1	11000
**202**	CHEMBL2431993	Cc1ccc(S(=O)(=O)N(CC(=O)N(Cc2ccc(C3CCCCC3)cc2)c2ccc(C(=O)O)c(O)c2)Cc2ccccc2C(F)(F)F)cc1	2800
**203**	CHEMBL2431985	CCCN(CC(=O)N(Cc1ccc(C2CCCCC2)cc1)c1ccc(C(=O)O)c(O)c1)S(=O)(=O)c1ccc(C)cc1	17800
**204**	CHEMBL2431967	Cc1ccc(S(=O)(=O)N(CC(=O)N(Cc2ccc(C3CCCCC3)cc2)c2ccc(C(=O)O)c(O)c2)Cc2cccc(OC(F)(F)F)c2)cc1	8000
**205**[a]	CHEMBL2431975	Cc1ccc(S(=O)(=O)N(CC(=O)N(Cc2ccc(C3CCCCC3)cc2)c2ccc(C(=O)O)c(O)c2)Cc2ccccn2)cc1	45200
**206**[a]	CHEMBL2431995	Cc1ccc(S(=O)(=O)N(CC(=O)N(Cc2ccc(C3CCCCC3)cc2)c2ccc(C(=O)O)c(O)c2)Cc2ccc(C(F)(F)F)cc2)cc1	9200
**207**[a]	CHEMBL1829876	CN(CC(=O)N(Cc1ccc(C2CCCCC2)cc1)c1ccc(C(=O)O)c(O)c1)S(=O)(=O)c1c(F)c(F)c(F)c(F)c1F	25600
**208**[a]	CHEMBL2431992	Cc1ccc(CN(CC(=O)N(Cc2ccc(C3CCCCC3)cc2)c2ccc(C(=O)O)c(O)c2)S(=O)(=O)c2ccc(C)cc2)cc1	50000
**209**	CHEMBL2431932	O=C(O)c1ccc(N(Cc2ccc(C3CCCCC3)cc2)C(=O)CN(Cc2cc(F)c(F)c(F)c2F)S(=O)(=O)c2c(F)c(F)c(F)c(F)c2F)cc1O	12400
**210**	CHEMBL2431955	O=C(O)c1ccc(N(Cc2ccc(C3CCCCC3)cc2)C(=O)CN(Cc2ccccc2C(F)(F)F)S(=O)(=O)c2c(F)c(F)c(F)c(F)c2F)cc1O	12800
**211**	CHEMBL2431969	Cc1ccc(S(=O)(=O)N(CC(=O)N(Cc2ccc(C3CCCCC3)cc2)c2ccc(C(=O)O)c(O)c2)Cc2ccccc2[N+](=O)[O-])cc1	10000
**212**[a]	CHEMBL2431986	Cc1ccc(S(=O)(=O)N(CC(=O)N(Cc2ccc(C3CCCCC3)cc2)c2ccc(C(=O)O)c(O)c2)CC(C)C)cc1	17000
**213**	CHEMBL2431981	Cc1ccc(S(=O)(=O)NCC(=O)Nc2ccc(C(=O)O)c(O)c2)cc1	300000
**214**	CHEMBL2431961	Cc1ccc(S(=O)(=O)N(CC(=O)N(Cc2ccc(C3CCCCC3)cc2)c2ccc(C(=O)O)c(O)c2)Cc2cccc(F)c2)cc1	9000
**215**	CHEMBL2023989	Cc1ccc(S(=O)(=O)N(CC(=O)N(Cc2ccc(C3CCCCC3)cc2)c2ccc(C(=O)O)c(O)c2)C(=O)OC(C)(C)C)cc1	115000
**216**	CHEMBL2431974	Cc1ccc(S(=O)(=O)N(CC(=O)N(Cc2ccc(C3CCCCC3)cc2)c2ccc(C(=O)O)c(O)c2)Cc2ccc(N)cc2)cc1	20800
**217**	CHEMBL2431984	Cc1ccc(S(=O)(=O)NCC(=O)N(Cc2ccc(C3CCCCC3)cc2)c2ccc(C(=O)O)c(O)c2)cc1	50000
**218**	CHEMBL2431939	Cc1ccc(S(=O)(=O)N(CC(=O)N(Cc2ccc(C3CCCCC3)cc2)c2ccc(C(=O)O)c(O)c2)Cc2c(F)cc(F)c(F)c2F)cc1	15400

**219**	CHEMBL2431968	Cc1ccc(S(=O)(=O)N(CC(=O)N(Cc2ccc(C3CCCCC3)cc2)c2ccc(C(=O)O)c(O)c2)Cc2ccc(OC(F)(F)F)cc2)cc1	11800
**220**	CHEMBL2431988	Cc1ccc(S(=O)(=O)N(CC(N)=O)CC(=O)N(Cc2ccc(C3CCCCC3)cc2)c2ccc(C(=O)O)c(O)c2)cc1	50000
**221[a]**	CHEMBL2431936	O=C(O)c1ccc(N(Cc2ccc(C3CCCCC3)cc2)C(=O)CN(Cc2ccc3ccccc3c2)S(=O)(=O)c2c(F)c(F)c(F)c(F)c2F)cc1O	21800
**222**	CHEMBL2431949	CCCN(CC(=O)N(Cc1ccc(C2CCCCC2)cc1)c1ccc(C(=O)O)c(O)c1)S(=O)(=O)c1c(F)c(F)c(F)c(F)c1F	20600
**223**	CHEMBL2431923	O=C(O)c1ccc(N(Cc2ccc(C3CCCCC3)cc2)C(=O)CN(Cc2ccccc2Cl)S(=O)(=O)c2c(F)c(F)c(F)c(F)c2F)cc1O	11000
**224[a]**	CHEMBL2431925	O=C(O)c1ccc(N(Cc2ccc(C3CCCCC3)cc2)C(=O)CN(Cc2ccc(Cl)cc2)S(=O)(=O)c2c(F)c(F)c(F)c(F)c2F)cc1O	13800
**225**	CHEMBL2431958	O=C(O)c1ccc(N(Cc2ccc(C3CCCCC3)cc2)C(=O)CN(Cc2ccccc2F)S(=O)(=O)c2c(F)c(F)c(F)c(F)c2F)cc1O	28000
**226**	CHEMBL2431946	COC(=O)c1cccc(CN(CC(=O)N(Cc2ccc(C3CCCCC3)cc2)c2ccc(C(=O)O)c(O)c2)S(=O)(=O)c2ccc(C)cc2)c1	18400
**227[a]**	CHEMBL2431982	Cc1ccc(S(=O)(=O)N(C)CC(=O)Nc2ccc(C(=O)O)c(O)c2)cc1	300000
**228[a]**	CHEMBL2172013	Cc1ccc(S(=O)(=O)OCC(=O)N(Cc2ccc(C3CCCCC3)cc2)c2ccc(C(=O)O)c(O)c2)cc1	43000
**229**	CHEMBL2431928	O=C(O)c1ccc(N(Cc2ccc(C3CCCCC3)cc2)C(=O)CN(Cc2ccc(OC(F)(F)F)cc2)S(=O)(=O)c2c(F)c(F)c(F)c(F)c2F)cc1O	21600
**230**	CHEMBL2431979	Cc1ccc(S(=O)(=O)N(CC(=O)N(Cc2ccc(C3CCCCC3)cc2)c2ccc(C(=O)O)c(O)c2)Cc2cccc(C#N)c2)cc1	12400
**231**	CHEMBL2431972	Cc1ccc(S(=O)(=O)N(CC(=O)N(Cc2ccc(C3CCCCC3)cc2)c2ccc(C(=O)O)c(O)c2)Cc2ccccc2N)cc1	24000
**232**	CHEMBL2431989	Cc1ccc(S(=O)(=O)N(CC(=O)N(Cc2ccc(C3CCCCC3)cc2)c2ccc(C(=O)O)c(O)c2)Cc2ccccc2)cc1	14400
**233[a]**	CHEMBL2431950	CC(C)(C)OC(=O)N(CC(=O)N(Cc1ccc(C2CCCCC2)cc1)c1ccc(C(=O)O)c(O)c1)S(=O)(=O)c1c(F)c(F)c(F)c(F)c1F	27400
**234**	CHEMBL2431964	Cc1ccc(S(=O)(=O)N(CC(=O)N(Cc2ccc(C3CCCCC3)cc2)c2ccc(C(=O)O)c(O)c2)Cc2cccc(Cl)c2)cc1	6000
**235**	CHEMBL2431976	Cc1ccc(S(=O)(=O)N(CC(=O)N(Cc2ccc(C3CCCCC3)cc2)c2ccc(C(=O)O)c(O)c2)Cc2cccnc2)cc1	50000
**236**	CHEMBL2431941	Cc1ccc(S(=O)(=O)N(CC(=O)N(Cc2ccc(C3CCCCC3)cc2)c2ccc(C(=O)O)c(O)c2)Cc2c(F)c(F)c(F)c(F)c2F)cc1	8100

**237**	CHEMBL2431978	Cc1ccc(S(=O)(=O)N(CC(=O)N(Cc2ccc(C3CCCCC3)cc2)c2ccc(C(=O)O)c(O)c2)Cc2ccccc2C#N)cc1	22600
**238**	CHEMBL2431951	O=C(O)c1ccc(N(Cc2ccc(C3CCCCC3)cc2)C(=O)CN(Cc2ccccc2)S(=O)(=O)c2c(F)c(F)c(F)c(F)c2F)cc1O	14400
**239**	CHEMBL2431956	O=C(O)c1ccc(N(Cc2ccc(C3CCCCC3)cc2)C(=O)CN(Cc2cccc(C(F)(F)F)c2)S(=O)(=O)c2c(F)c(F)c(F)c(F)c2F)cc1O	9600
**240**	CHEMBL2431938	COC(=O)c1cccc(CN(CC(=O)N(Cc2ccc(C3CCCCC3)cc2)c2ccc(C(=O)O)c(O)c2)S(=O)(=O)c2c(F)c(F)c(F)c(F)c2F)c1	30800
**241[a]**	CHEMBL2431931	N#Cc1ccc(CN(CC(=O)N(Cc2ccc(C3CCCCC3)cc2)c2ccc(C(=O)O)c(O)c2)S(=O)(=O)c2c(F)c(F)c(F)c(F)c2F)cc1	9400
**242[a]**	CHEMBL2431953	Cc1cccc(CN(CC(=O)N(Cc2ccc(C3CCCCC3)cc2)c2ccc(C(=O)O)c(O)c2)S(=O)(=O)c2c(F)c(F)c(F)c(F)c2F)c1	14600
**243**	CHEMBL2431948	O=C(O)c1ccc(N(Cc2ccc(C3CCCCC3)cc2)C(=O)CNS(=O)(=O)c2c(F)c(F)c(F)c(F)c2F)cc1O	32400
**244**	CHEMBL2431957	O=C(O)c1ccc(N(Cc2ccc(C3CCCCC3)cc2)C(=O)CN(Cc2ccc(C(F)(F)F)cc2)S(=O)(=O)c2c(F)c(F)c(F)c(F)c2F)cc1O	22000
**245**	CHEMBL2431987	C=CCN(CC(=O)N(Cc1ccc(C2CCCCC2)cc1)c1ccc(C(=O)O)c(O)c1)S(=O)(=O)c1ccc(C)cc1	50000
**246[a]**	CHEMBL2431965	Cc1ccc(S(=O)(=O)N(CC(=O)N(Cc2ccc(C3CCCCC3)cc2)c2ccc(C(=O)O)c(O)c2)Cc2ccc(Cl)cc2)cc1	6200
**247**	CHEMBL2431927	O=C(O)c1ccc(N(Cc2ccc(C3CCCCC3)cc2)C(=O)CN(Cc2cccc(OC(F)(F)F)c2)S(=O)(=O)c2c(F)c(F)c(F)c(F)c2F)cc1O	17800
**248**	CHEMBL2431934	O=C(O)c1ccc(N(Cc2ccc(C3CCCCC3)cc2)C(=O)CN(Cc2c(F)c(F)cc(F)c2F)S(=O)(=O)c2c(F)c(F)c(F)c(F)c2F)cc1O	16600
**249[a]**	CHEMBL2431945	CCOC(=O)c1ccc(CN(CC(=O)N(Cc2ccc(C3CCCCC3)cc2)c2ccc(C(=O)O)c(O)c2)S(=O)(=O)c2ccc(C)cc2)cc1	7600
**250[a]**	CHEMBL2431942	Cc1ccc(S(=O)(=O)N(CC(=O)N(Cc2ccc(C3CCCCC3)cc2)c2ccc(C(=O)O)c(O)c2)Cc2ccc3ccccc3c2)cc1	5000
**251**	CHEMBL2431966	Cc1ccc(S(=O)(=O)N(CC(=O)N(Cc2ccc(C3CCCCC3)cc2)c2ccc(C(=O)O)c(O)c2)Cc2ccccc2OC(F)(F)F)cc1	7200
**252**	CHEMBL2431970	Cc1ccc(S(=O)(=O)N(CC(=O)N(Cc2ccc(C3CCCCC3)cc2)c2ccc(C(=O)O)c(O)c2)Cc2cccc([N+](=O)[O-])c2)cc1	5000
**253**	CHEMBL2431983	Cc1ccc(S(=O)(=O)N(CC(=O)Nc2ccc(C(=O)O)c(O)c2)C(=O)OC(C)(C)C)cc1	300000
**254**	CHEMBL2431943	Cc1ccc(S(=O)(=O)N(CC(=O)N(Cc2ccc(C3CCCCC3)cc2)c2ccc(C(=O)O)c(O)c2)Cc2ccc(F)cc2C(F)(F)F)cc1	11000

**255**	CHEMBL2431933	O=C(O)c1ccc(N(Cc2ccc(C3CCCCC3)cc2)C(=O)CN(Cc2c(F)cc(F)c(F)c2F)S(=O)(=O)c2c(F)c(F)c(F)c(F)c2F)cc1O	17600
**256**	CHEMBL2431963	Cc1ccc(S(=O)(=O)N(CC(=O)N(Cc2ccc(C3CCCCC3)cc2)c2ccc(C(=O)O)c(O)c2)Cc2ccccc2Cl)cc1	15800
**257**	CHEMBL2431935	O=C(O)c1ccc(N(Cc2ccc(C3CCCCC3)cc2)C(=O)CN(Cc2c(F)c(F)c(F)c(F)c2F)S(=O)(=O)c2c(F)c(F)c(F)c(F)c2F)cc1O	11000
**258**	CHEMBL2431947	COC(=O)c1ccc(CN(CC(=O)N(Cc2ccc(C3CCCCC3)cc2)c2ccc(C(=O)O)c(O)c2)S(=O)(=O)c2ccc(C)cc2)cc1	11600
**259**	CHEMBL2431954	Cc1ccc(CN(CC(=O)N(Cc2ccc(C3CCCCC3)cc2)c2ccc(C(=O)O)c(O)c2)S(=O)(=O)c2c(F)c(F)c(F)c(F)c2F)cc1	14400
**260**	CHEMBL2431994	Cc1ccc(S(=O)(=O)N(CC(=O)N(Cc2ccc(C3CCCCC3)cc2)c2ccc(C(=O)O)c(O)c2)Cc2cccc(C(F)(F)F)c2)cc1	10200
**261[a]**	CHEMBL2431937	COC(=O)c1ccccc1CN(CC(=O)N(Cc1ccc(C2CCCCC2)cc1)c1ccc(C(=O)O)c(O)c1)S(=O)(=O)c1c(F)c(F)c(F)c(F)c1F	17400
**262[a]**	CHEMBL1829786	Cc1ccc(S(=O)(=O)N(C)CC(=O)N(Cc2ccc(C3CCCCC3)cc2)c2ccc(C(=O)O)c(O)c2)cc1	31000
**263**	CHEMBL2431952	Cc1ccccc1CN(CC(=O)N(Cc1ccc(C2CCCCC2)cc1)c1ccc(C(=O)O)c(O)c1)S(=O)(=O)c1c(F)c(F)c(F)c(F)c1F	11800
**264**	CHEMBL2431926	O=C(O)c1ccc(N(Cc2ccc(C3CCCCC3)cc2)C(=O)CN(Cc2ccccc2OC(F)(F)F)S(=O)(=O)c2c(F)c(F)c(F)c(F)c2F)cc1O	10200
**265**	CHEMBL2431944	Cc1ccc(S(=O)(=O)N(CC(=O)N(Cc2ccc(C3CCCCC3)cc2)c2ccc(C(=O)O)c(O)c2)Cc2ccc(SC(F)(F)F)cc2)cc1	9600
**266**	CHEMBL2431940	Cc1ccc(S(=O)(=O)N(CC(=O)N(Cc2ccc(C3CCCCC3)cc2)c2ccc(C(=O)O)c(O)c2)Cc2c(F)c(F)cc(F)c2F)cc1	22800
**267**	CHEMBL2431929	N#Cc1ccccc1CN(CC(=O)N(Cc1ccc(C2CCCCC2)cc1)c1ccc(C(=O)O)c(O)c1)S(=O)(=O)c1c(F)c(F)c(F)c(F)c1F	11400
**268**	CHEMBL2431980	Cc1ccc(S(=O)(=O)N(CC(=O)N(Cc2ccc(C3CCCCC3)cc2)c2ccc(C(=O)O)c(O)c2)Cc2ccc(C#N)cc2)cc1	8600
**269**	CHEMBL2431973	Cc1ccc(S(=O)(=O)N(CC(=O)N(Cc2ccc(C3CCCCC3)cc2)c2ccc(C(=O)O)c(O)c2)Cc2cccc(N)c2)cc1	12400
**270**	CHEMBL2431962	Cc1ccc(S(=O)(=O)N(CC(=O)N(Cc2ccc(C3CCCCC3)cc2)c2ccc(C(=O)O)c(O)c2)Cc2ccc(F)cc2)cc1	7600
**271[a]**	CHEMBL2431960	Cc1ccc(S(=O)(=O)N(CC(=O)N(Cc2ccc(C3CCCCC3)cc2)c2ccc(C(=O)O)c(O)c2)Cc2ccccc2F)cc1	10600
**272**	CHEMBL477936	Cc1ccc(S(=O)(=O)OCC(=O)Nc2ccc(C(=O)O)c(O)c2)cc1	84000

**273**	CHEMBL2431930	N#Cc1cccc(CN(CC(=O)N(Cc2ccc(C3CCCCC3)cc2)c2ccc(C(=O)O)c(O)c2)S(=O)(=O)c2c(F)c(F)c(F)c(F)c2F)c1	19800
**274**	CHEMBL3126485	CN(C)CCCN1C(=O)c2ccc3c4ccc5c6c(ccc(c7ccc(c2c37)C1=O)c64)C(=O)N(CCCN(C)C)C5=O	4500
**275**	CHEMBL3133482	O=C(O)c1cc(N(Cc2ccc(Cl)cc2)C(=O)CN(Cc2ccccc2)S(=O)(=O)c2ccc(-c3ccccc3)cc2)ccc1O	15000
**276**	CHEMBL3133484	O=C(O)c1cc(N(Cc2ccc(C3CCCCC3)cc2)C(=O)CN(Cc2ccccc2)S(=O)(=O)c2ccc(-c3ccccc3)cc2)ccc1O	23300
**277[a]**	CHEMBL3133490	Cc1ccc(S(=O)(=O)N(C)CC(=O)N(Cc2ccc(C3CCCCC3)cc2)c2ccc(O)c(C(=O)O)c2)cc1	45000
**278**	CHEMBL3133491	CN(CC(=O)N(Cc1ccc(C2CCCCC2)cc1)c1ccc(O)c(C(=O)O)c1)S(=O)(=O)c1ccc(-c2ccccc2)cc1	32000
**279**	CHEMBL3133493	CC(C)Cc1ccc(CN(C(=O)CN(C)S(=O)(=O)c2ccc(-c3ccc(Cl)cc3)cc2)c2ccc(O)c(C(=O)O)c2)cc1	20000
**280**	CHEMBL3133487	Cc1ccc(S(=O)(=O)N(C)CC(=O)N(Cc2ccccc2)c2ccc(O)c(C(=O)O)c2)cc1	1000000
**281**	CHEMBL3133486	CC(C)Cc1ccc(CN(C(=O)CN(Cc2ccccc2)S(=O)(=O)c2ccc(-c3ccccc3)cc2)c2ccc(O)c(C(=O)O)c2)cc1	17000
**282**	CHEMBL3133494	CN(CC(=O)N(Cc1ccc(Cl)cc1)c1ccc(O)c(C(=O)O)c1)S(=O)(=O)c1ccc(-c2ccc(Cl)cc2)cc1	43000
**283**	CHEMBL3133472	CN(CC(=O)N(Cc1cccc(-c2ccncc2)c1)c1ccc(O)c(C(=O)O)c1)S(=O)(=O)c1ccc(-c2ccccc2)cc1	35300
**284**	CHEMBL3133488	CN(CC(=O)N(Cc1ccccc1)c1ccc(O)c(C(=O)O)c1)S(=O)(=O)c1ccc(-c2ccccc2)cc1	57000
**285**	CHEMBL3133468	O=C(O)c1cc(N(Cc2ccc(C3CCNCC3)cc2)C(=O)CN(Cc2ccccc2)S(=O)(=O)c2ccc(-c3ccccc3)cc2)ccc1O	300000
**286**	CHEMBL3133454	COc1ccc(CN(C(=O)c2ccc(Oc3ccccc3)cc2)c2ccc(O)c(C(=O)O)c2)cc1	118100
**287**	CHEMBL3133492	CCCCCCCc1ccc(CN(C(=O)CN(C)S(=O)(=O)c2ccc(-c3ccccc3)cc2)c2ccc(O)c(C(=O)O)c2)cc1	22000
**288[a]**	CHEMBL3133483	COc1ccc(CN(C(=O)CN(Cc2ccccc2)S(=O)(=O)c2ccc(-c3ccccc3)cc2)c2ccc(O)c(C(=O)O)c2)cc1	22000
**289**	CHEMBL3133467	CCCCCCCc1ccc(CN(C(=O)CN(C)S(=O)(=O)c2ccc(-c3ccc(F)cc3)cc2)c2ccc(O)c(C(=O)O)c2)cc1	23000
**290**	CHEMBL3133459	O=C(O)c1cc(N(Cc2ccc(C3CCCCC3)cc2)C(=O)c2ccc(Oc3ccccc3)cc2)ccc1O	15000

**291**	CHEMBL3133475	O=C(c1ccc(Oc2ccccc2)cc1)N(Cc1ccc(C2CCCCC2)cc1)c1ccc(CP(=O)(O)O)cc1	28400
**292**	CHEMBL3133473	O=C(c1ccc(Oc2ccccc2)cc1)N(Cc1ccc(C2CCCCC2)cc1)c1ccc(P(=O)(O)O)cc1	42000
**293**[a]	CHEMBL3133485	CCCCCCCc1ccc(CN(C(=O)CN(Cc2ccccc2)S(=O)(=O)c2ccc(-c3ccccc3)cc2)c2ccc(O)c(C(=O)O)c2)cc1	19000
**294**	CHEMBL3133466	COc1ccc(CN(C(=O)CN(C)S(=O)(=O)c2ccc(-c3ccc(Cl)cc3)cc2)c2ccc(O)c(C(=O)O)c2)cc1	50000
**295**	CHEMBL3133469	O=C(O)c1cc(N(Cc2cccc(-c3ccncc3)c2)C(=O)CN(Cc2ccccc2)S(=O)(=O)c2ccc(-c3ccccc3)cc2)ccc1O	33500
**296**	CHEMBL3133489	CN(CC(=O)N(Cc1ccc(Cl)cc1)c1ccc(O)c(C(=O)O)c1)S(=O)(=O)c1ccc(-c2ccccc2)cc1	61000
**297**	CHEMBL3133460	CCCCCCCc1ccc(CN(C(=O)c2ccccc2)c2ccc(O)c(C(=O)O)c2)cc1	32000
**298**	CHEMBL3133458	CCCCCCCc1ccc(CN(C(=O)c2ccc(Oc3ccccc3)cc2)c2ccc(O)c(C(=O)O)c2)cc1	12800
**299**	CHEMBL3133481	Cc1ccc(S(=O)(=O)N(CC(=O)N(Cc2ccc(Cl)cc2)c2ccc(O)c(C(=O)O)c2)Cc2ccccc2)cc1	50000
**300**[a]	CHEMBL3133470	O=C(O)c1cc(N(Cc2ccc(N3CCOCC3)cc2)C(=O)CN(Cc2ccccc2)S(=O)(=O)c2ccc(-c3ccccc3)cc2)ccc1O	94700
**301**[a]	CHEMBL3133461	CCCCCCCc1ccc(CN(C(=O)c2cccc(OC)c2)c2ccc(O)c(C(=O)O)c2)cc1	33300
**302**	CHEMBL3133456	O=C(O)c1cc(N(Cc2ccc(Cl)cc2)C(=O)c2ccc(Oc3ccccc3)cc2)ccc1O	48000
**303**[a]	CHEMBL3133496	O=C(O)c1cc(N(Cc2ccccc2)C(=O)CN(Cc2ccccc2)S(=O)(=O)c2ccc(-c3ccccc3)cc2)ccc1O	22200
**304**	CHEMBL3133476	O=C(c1ccc(Oc2ccccc2)cc1)N(Cc1ccc(C2CCCCC2)cc1)c1cccc(CP(=O)(O)O)c1	18900
**305**	CHEMBL3133495	Cc1ccc(S(=O)(=O)N(CC(=O)N(Cc2ccccc2)c2ccc(O)c(C(=O)O)c2)Cc2ccccc2)cc1	201300
**306**	CHEMBL3133457	O=C(O)c1cc(N(Cc2ccc(Br)cc2)C(=O)c2ccc(Oc3ccccc3)cc2)ccc1O	52000
**307**	CHEMBL1241908	O=C(N/N=C/c1coc2ccccc2c1=O)c1cccnc1	180000
**308**	CHEMBL3218348	O=C(Nc1nonc1-c1ccc(Cl)cc1)c1ccc(C(F)(F)F)cc1	17700
**309**	CHEMBL3600746	COc1ccc(S(=O)(=O)Nc2ccccc2-c2ccc(C#N)cc2)cc1	440
**310**	CHEMBL3600853	COc1ccc(S(=O)(=O)N(C(C)=O)c2ccccc2-c2ccc(C#N)cc2)cc1	320
**311**	CHEMBL3600755	COc1ccc(S(=O)(=O)Nc2ccccc2-c2ccc(F)c(F)c2)cc1	2750
**312**[a]	CHEMBL3600757	COc1ccc(S(=O)(=O)Nc2ccccc2-c2ccc(C#N)c(F)c2)cc1	200

**313**	CHEMBL3600744	COc1ccc(S(=O)(=O)Nc2ccccc2-c2ccc(F)cc2)cc1	4020
**314**	CHEMBL3628691	COc1cc(CNc2nn[nH]n2)cc(Cl)c1OCc1ccc([N+](=O)[O-])cc1	25700
**315[a]**	CHEMBL4227152	C=C(C)C(=O)N(Cc1ccc(Br)cc1)C(=O)/C=C/c1cc(OC)c(OC)c(OC)c1	10000
**316**	CHEMBL465843	COc1cc(/C=C/C(=O)N2CCC=CC2=O)cc(OC)c1OC	7000

Table S2: STAT3 complexes in Protein Data Bank (PDB).

Protein Data bank code[a]	Resolution (Å)	Molecular weight of Co-crystallized ligand	Ligand affinity Ki (nM)	Description	Reference
4ZIA	2.7	not found	not found	Crystal Structure of STAT3 N-terminal domain	31
3CWG	3.05	not found	not found	Unphosphorylated mouse STAT3 core fragment	32
6QHD	2.85	not found	not found	Lysine acetylated and tyrosine phosphorylated STAT3 in a complex with DNA	33
4E68	2.59	not found	not found	Unphosphorylated STAT3B core protein binding to dsDNA	34
5U5S	Solution NMR	188.22 N(6)-Acetyl lysine	not found	Solution structures of Brd2 second bromodomain in complex with stat3 peptide	35
6NJS	2.7	835.79 KQV	14 nM	Stat3 Core in complex with compound SD36	36
6NUQ	3.15	835.79 KQV	14 nM	Stat3 Core in complex with compound SI109	36
1BG1	2.25	not found	not found	Transcription Factor Stat3b/DNA Complex	37
6TLC	2.9	not found	not found	Unphosphorylated human STAT3 in complex with MS3-6 monobody	38

[a]Searching the PDB database for STAT3 complexes identified 32 protein structures, out of which only 9 are STAT3 protein structures (listed in the table above), the rest are STAT3-related cellular signaling proteins and were therefore neglected. Two of the nine STAT3 structures are short fragments and unsuitable for docking purposes (namely, 4ZIA and 5U5S, respectively).

Table S3: Cross correlation (R^2) matrix of the 9 docking- scoring functions used in this experiment [a,b].

	LigScore 1	LigScore 2	PLP1	PLP2	Jain	PMF	PMF04	Cdocker energy	Cdocker interaction energy
LigScore 1	1.00								
LigScore 2	0.53	1.00							
PLP1	0.28	0.71	1.00						
PLP2	0.27	0.70	0.97	1.00					
Jain	0.17	0.52	0.80	0.84	1.00				
PMF	0.19	0.50	0.60	0.59	0.47	1.00			
PMF04	0.16	0.49	0.57	0.57	0.44	0.86	1.00		
Cdocker energy	0.00	0.00	0.00	0.00	0.00	0.00	0.00	1.00	
Cdocker interaction energy	0.05	0.28	0.42	0.45	0.48	0.24	0.24	0.00	1.00

[a] A value of 0 implies that there is no linear dependency between the two variables. While a value of 1 indicates the existence of a perfect linear equation connecting the corresponding scoring functions pair.

[b] Cross correlation equation value exceeding 0.9 implies significant linearity between the two variables.

Table S4: Independent samples T-test statistical analysis and related statistical criteria for evaluating the difference between highly potent compounds (IC_{50} < 1.0 µM) and less potent compounds (IC_{50} ≥ 1.0 µM) within the "active" category in SHAP selected descriptors used for building Hypo-1 and Hypo-2.

SHAP descriptors	Group Statistical criteria[a]				t-test for equality of means (α = 0.05)
	Group[b,c]	Observations (N)	Mean	Standard deviation	*p*-value (2-tailed)
**PLP2**	IC_{50} ≥ 1.0 µM	4045	93.84	34.44	0.000
	IC_{50} < 1.0 µM	5384	96.84	35.413	
**PMF**	IC_{50} ≥ 1.0 µM	4045	75.62	28.61	0.036
	IC_{50} < 1.0 µM	5384	76.90	30.03	
**Cdocker Energy**	IC_{50} ≥ 1.0 µM	4045	$-11.93*10^{13}$	$75.32*10^{14}$	0.318
	IC_{50} < 1.0 µM	5384	$-10.66*10^{11}$	$33.63*10^{12}$	
**Cdocker Interaction Energy**	IC_{50} ≥ 1.0 µM	4045	-65.16	145.25	0.031
	IC_{50} < 1.0 µM	5384	-58.53	148.96	
**SER 613 HN**	IC_{50} ≥ 1.0 µM	4045	0.28	0.45	0.602
	IC50 < 1.0 µM	5384	0.28	0.45	

[a]Docked poses of all active compounds (training and testing) were considered for the analysis

[b] Docked poses corresponding to active docked compounds with IC_{50} ≥ 1.0 µM.

[c] Docked poses corresponding to active docked compounds with IC_{50} < 1.0 µM.

Table S5: Forward and reverse primer sequences for STAT3 target genes analyzed in q-PCR

Gene	Forward primer	Reverse primer
c-myc	GGATTCTCTGCTCTCCTCGAC	CTTCTTGTTCCTCCTCAGAGTC
Bcl-XL	CCAGGGTCTTCCCTACCTCA	GACTGGCCCTTCTAGTCAGC

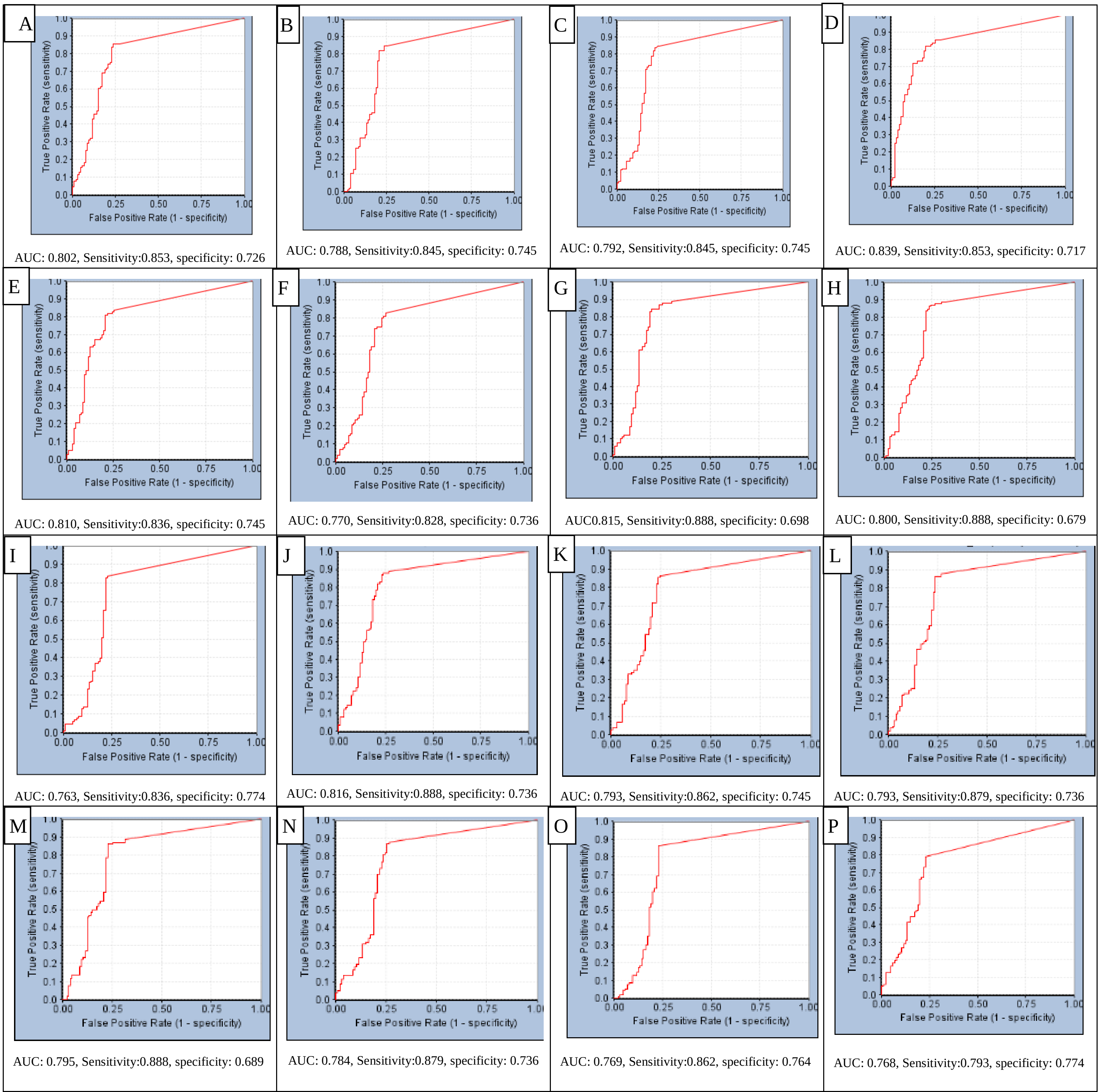

Figure S1: ROC curves of pharmacophores generated from crystallographic complexes 6njs and 6nuq : **A.** Hypo(6njs-04), **B.** Hypo(6njs-05), **C.** Hypo(6njs-06), **D.** Hypo(6njs-08), **E.** Hypo(6njs-09), **F.** Hypo(6njs-10), **G.** Hypo(6nuq-01), **H.** Hypo(6nuq-02), **I.** Hypo(6nuq-03), **J.** Hypo(6nuq-04), **K.** Hypo(6nuq-05), **L.** Hypo(6nuq-06), **M.** Hypo(6nuq-07), **N.** Hypo(6nuq-08), **O.** Hypo(6nuq-09), **P.** Hypo(6nuq-10).

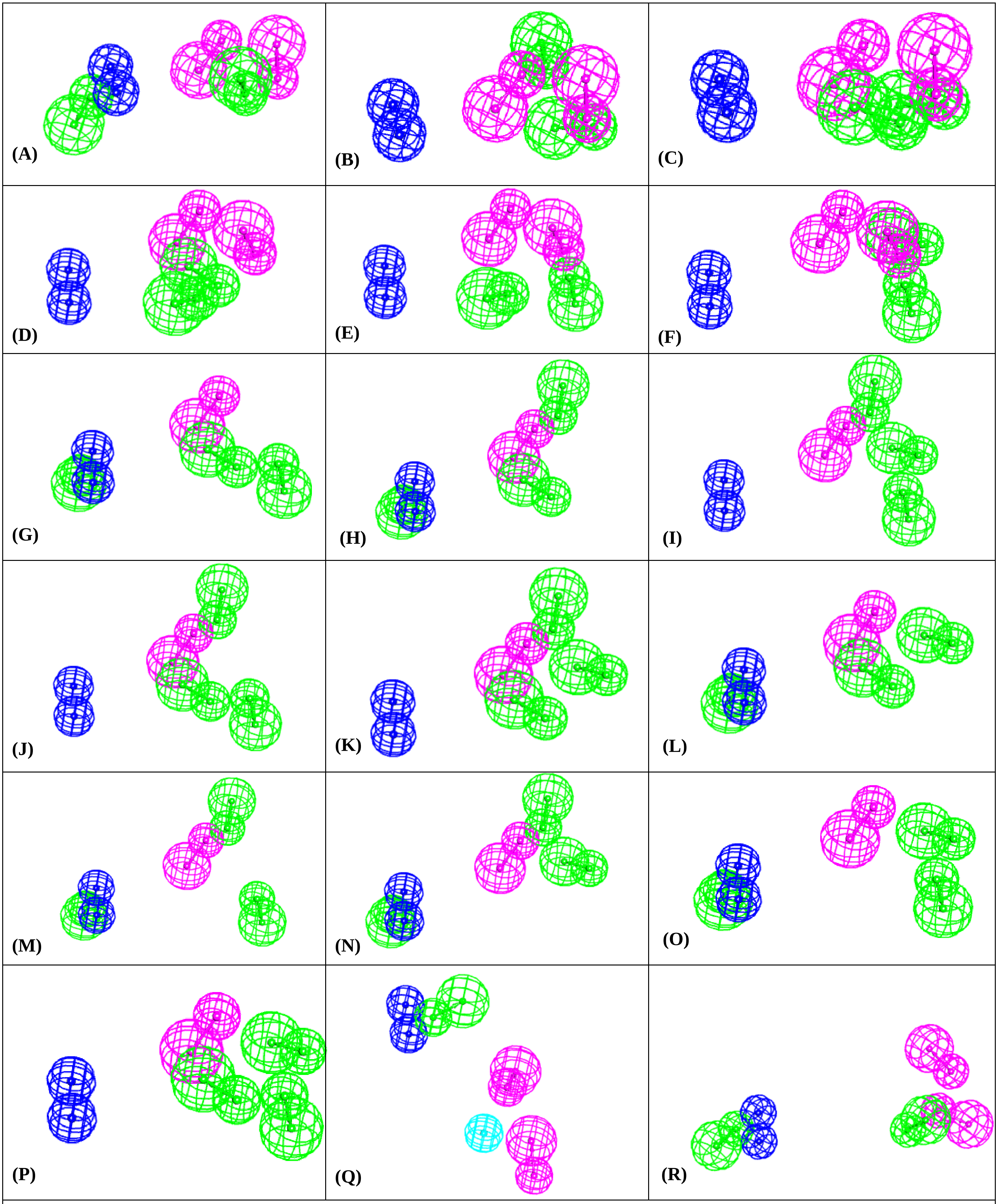

Figure S2: Pharmacophores generated from crystallographic complexes 6njs and 6nuq. **(A)** Hypo(6njs-04), **(B)** Hypo(6njs-05), **(C)** Hypo(6njs-06), **(D)** Hypo(6njs-08), **(E)** Hypo(6njs-09), (F) Hypo(6njs-10), **(G)** Hypo(6nuq-01), **(H)** Hypo(6nuq-02), **(I)** Hypo(6nuq-03), **(J)** Hypo(6nuq-04), **(K)** Hypo(6nuq-05), **(L)** Hypo(6nuq-06), **(M)** Hypo(6nuq-07), **(N)** Hypo(6nuq-08), **(O)** Hypo(6nuq-09), **(P)** Hypo(6nuq-10), **(Q)** Hypo-1 and **(R)** Hypo-2.

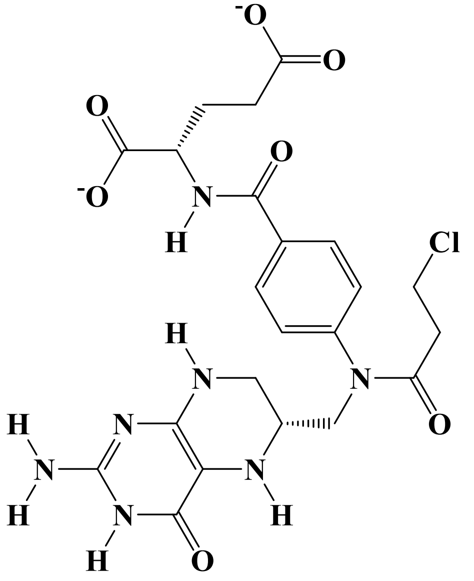

317 (NCI code: 3590)

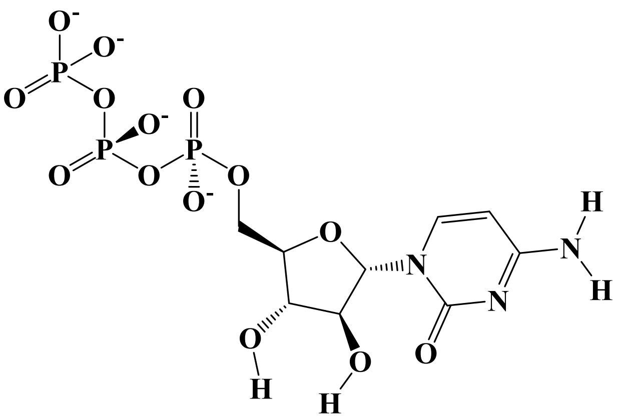

318 (NCI code: 20261)

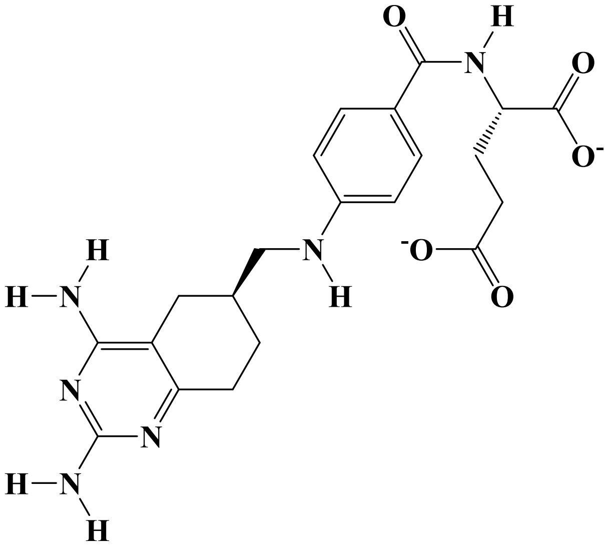

319 (NCI code: 59407)

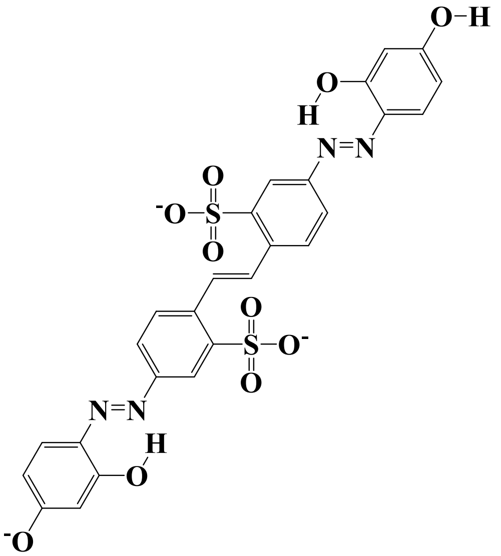

320 (NCI code:65832)

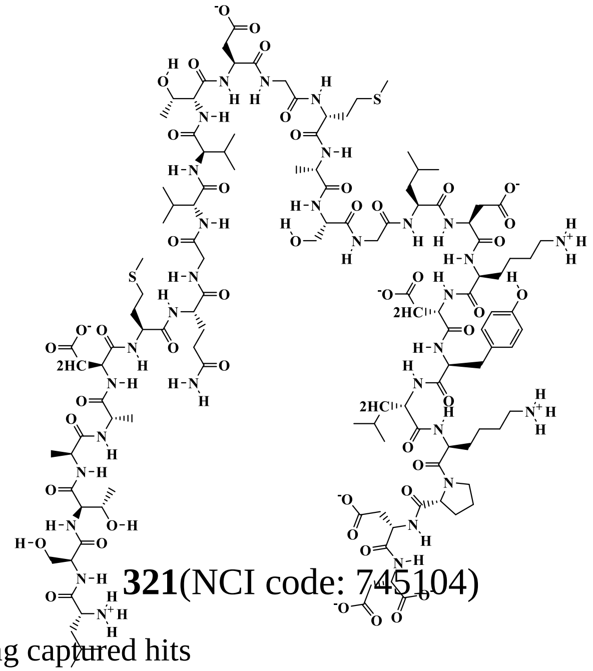

321(NCI code: 745104)

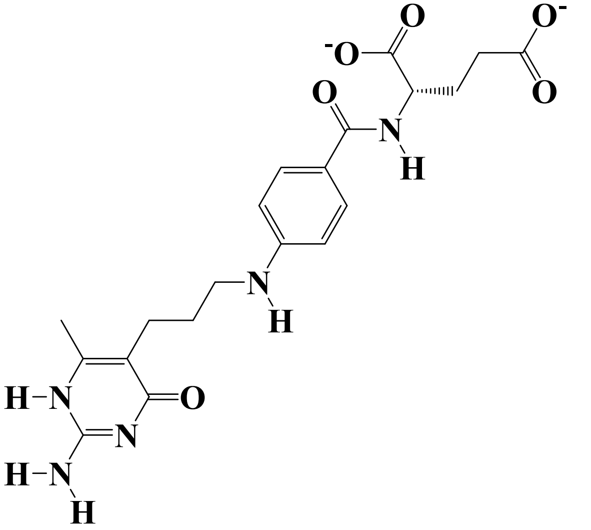

322 (NCI code: 72868)

Figure S3. Chemical structures of high-ranking captured hits

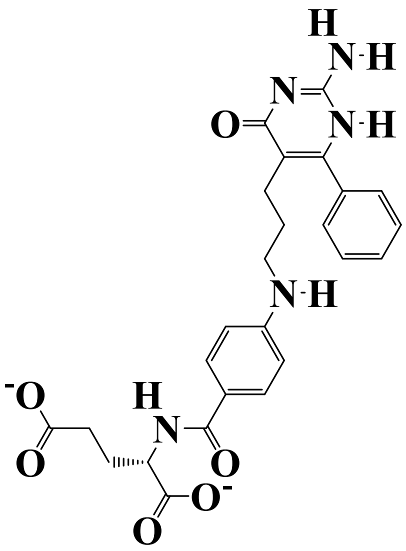

323 (NCI code: 77028)

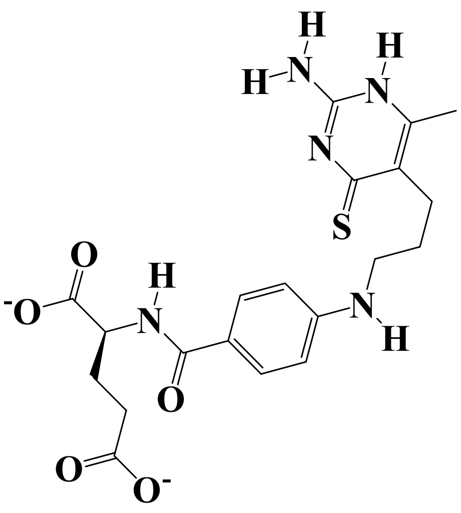

324 (NCI code: 77029)

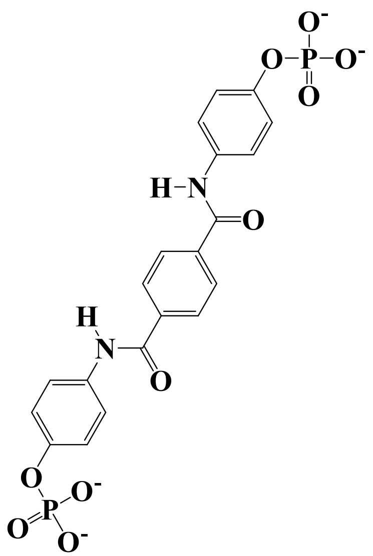

325 (NCI code: 82523)

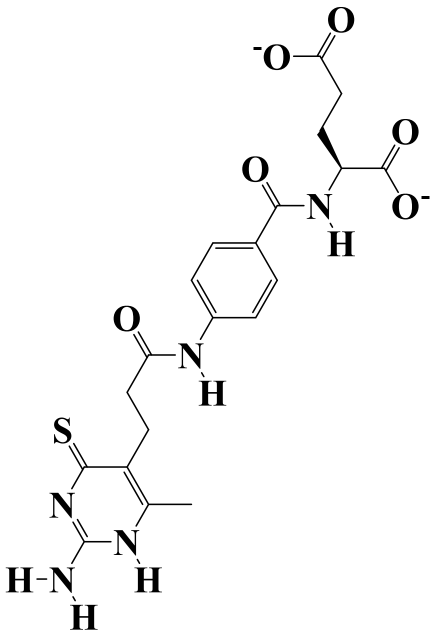

326 (NCI code: 98711)

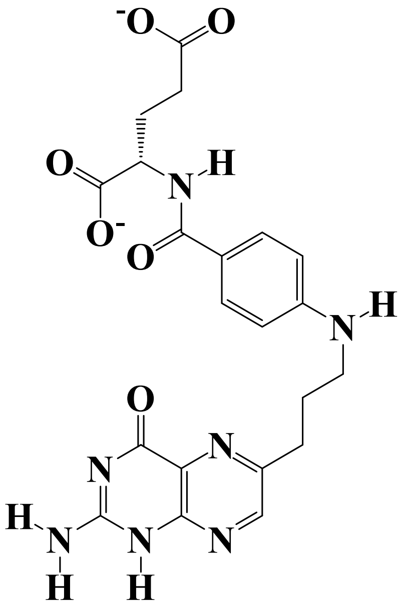

327 (NCI code: 100791)

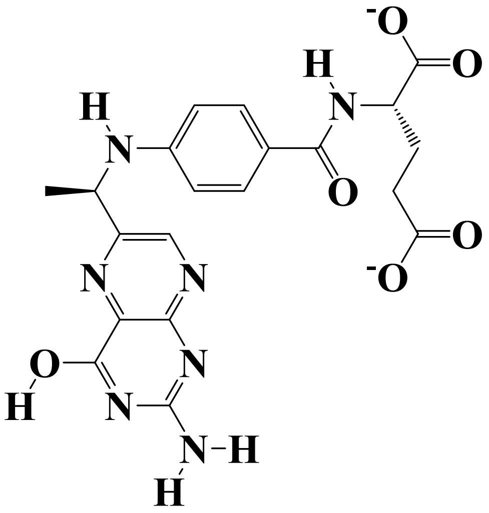

328 (NCI code: 107137)

Figure S3. Chemical structures of high-ranking captured hits

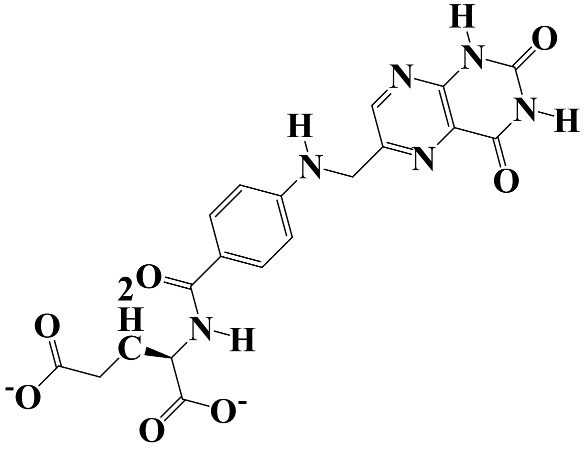

329 (NCI code: 107139)

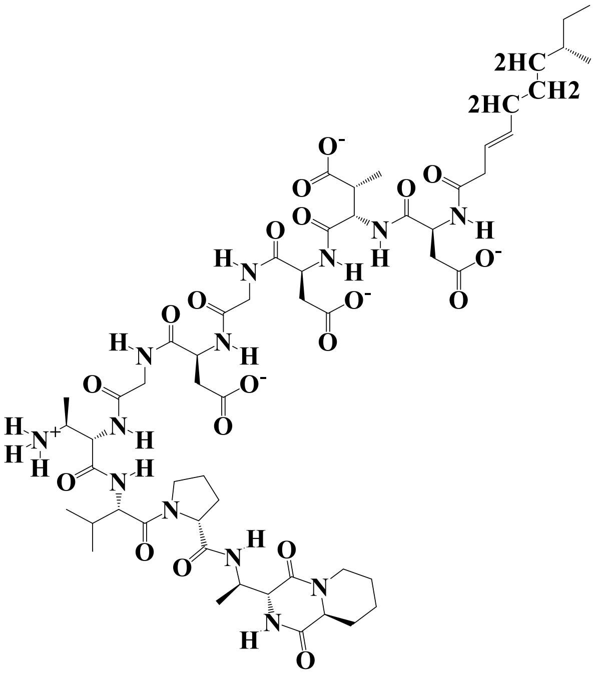

330 (NCI code: 267431)

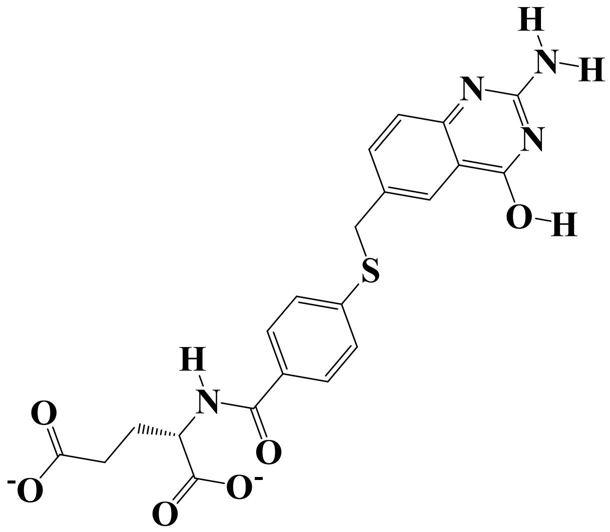

331 (NCI code: 289523)

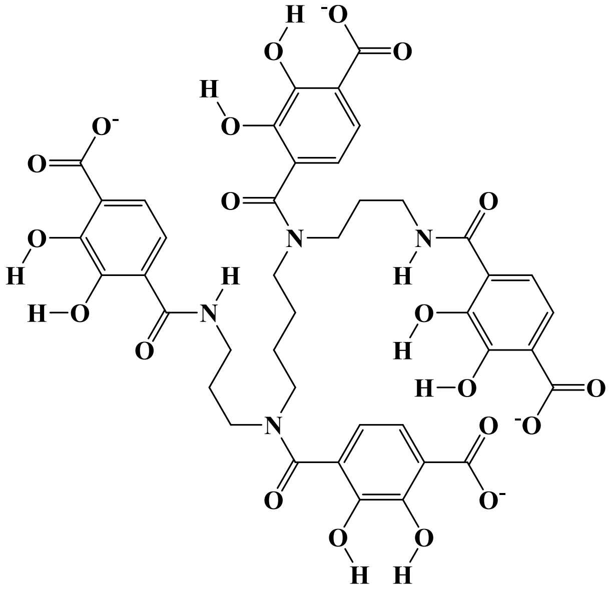

332 (NCI code: 338310)

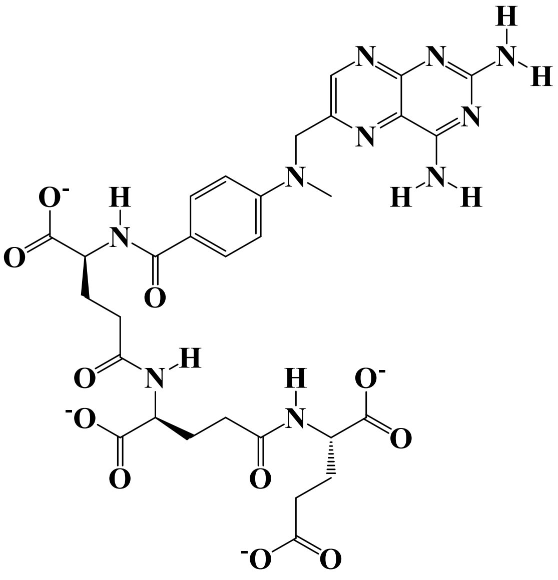

333 (NCI code: 341076)

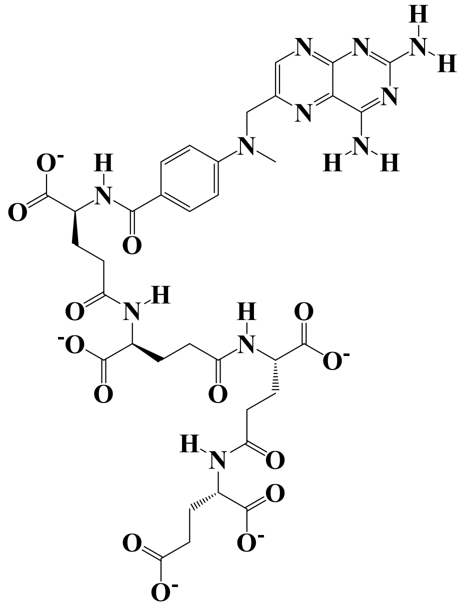

334 (NCI code: 341077)

Figure S3. Chemical structures of high-ranking captured hits

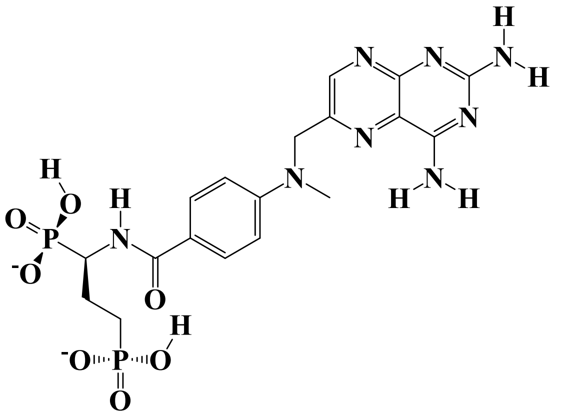

335 (NCI code: 363007)

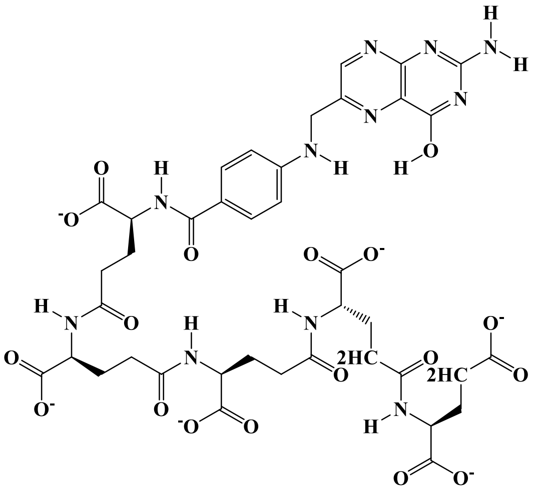

336 (NCI code: 372667)

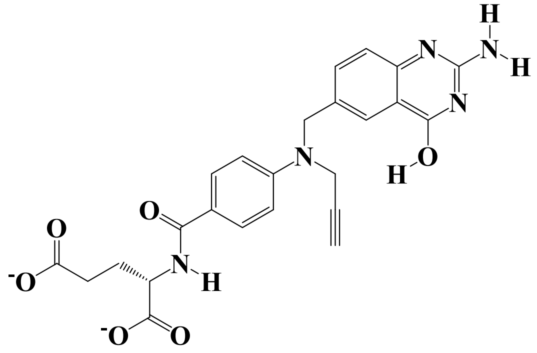

337 (NCI code: 373233)

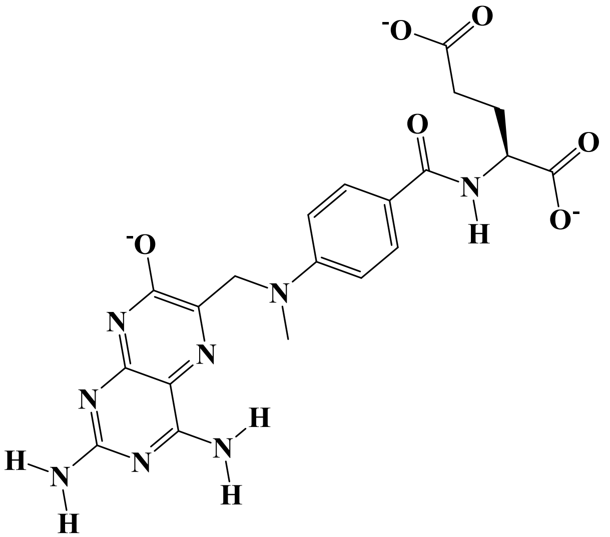

338 (NCI code: 380962)

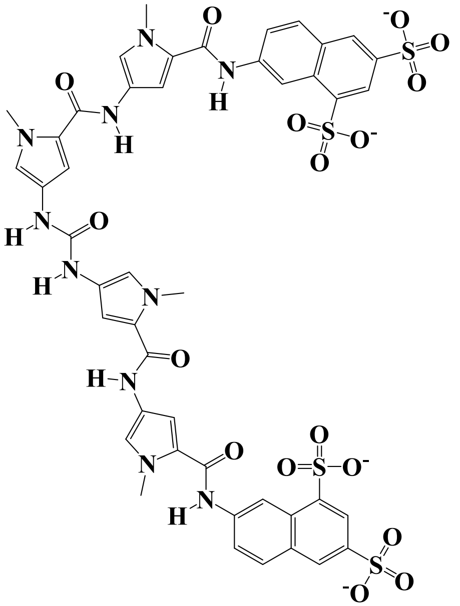

339 (NCI code: 645793)

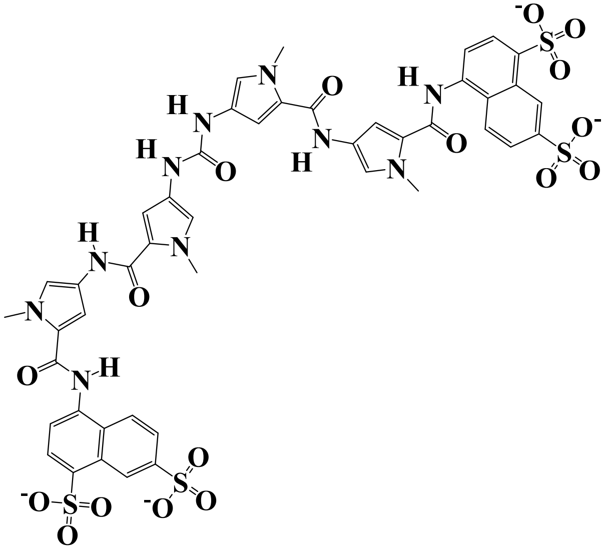

340 (NCI code: 651016)

Figure S3. Chemical structures of high-ranking captured hits

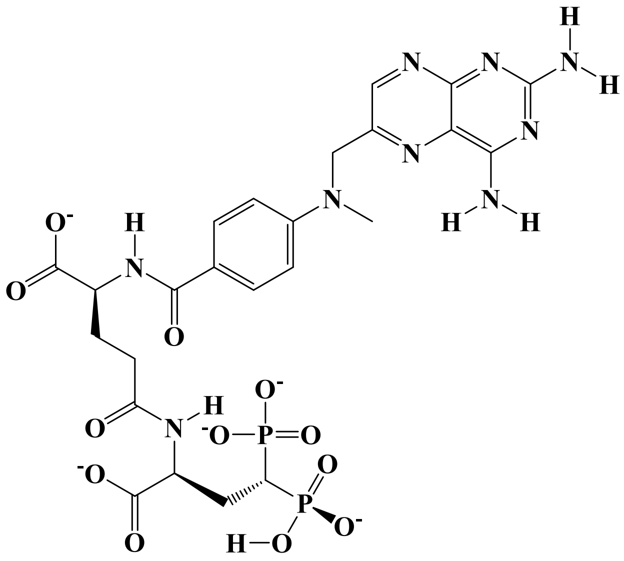

341 (NCI code: 669269)

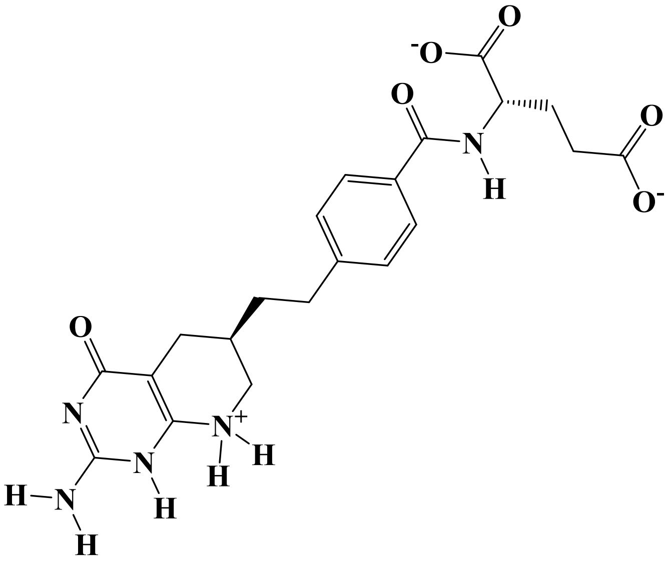

342 (NCI code: 722969)

Figure S3. Chemical structures of high-ranking captured hits
